# N-terminal acetylation shields proteins from degradation and promotes age-dependent motility and longevity

**DOI:** 10.1038/s41467-023-42342-y

**Published:** 2023-10-27

**Authors:** Sylvia Varland, Rui Duarte Silva, Ine Kjosås, Alexandra Faustino, Annelies Bogaert, Maximilian Billmann, Hadi Boukhatmi, Barbara Kellen, Michael Costanzo, Adrian Drazic, Camilla Osberg, Katherine Chan, Xiang Zhang, Amy Hin Yan Tong, Simonetta Andreazza, Juliette J. Lee, Lyudmila Nedyalkova, Matej Ušaj, Alexander J. Whitworth, Brenda J. Andrews, Jason Moffat, Chad L. Myers, Kris Gevaert, Charles Boone, Rui Gonçalo Martinho, Thomas Arnesen

**Affiliations:** 1 Department of Biomedicine, University of Bergen, N-5021 Bergen, Norway; 2 Department of Biological Sciences, University of Bergen, N-5006 Bergen, Norway; 3 The Donnelly Centre for Cellular and Biomolecular Research, University of Toronto, Toronto, ON M5S 3E1 Canada; 4 Algarve Biomedical Center Research Institute, Universidade do Algarve, 8005−139 Faro, Portugal; 5 Faculdade de Medicina e Ciências Biomédicas, Universidade do Algarve, 8005−139 Faro, Portugal; 6 VIB-UGent Center for Medical Biotechnology, B-9052 Ghent, Belgium; 7 Department of Biomolecular Medicine, Ghent University, B-9052 Ghent, Belgium; 8 Department of Computer Science and Engineering, University of Minnesota-Twin Cities, Minneapolis, MN 55455 USA; 9 Institute of Human Genetics, University of Bonn, School of Medicine and University Hospital Bonn, D-53127 Bonn, Germany; 10 Institut de Génétique et Développement de Rennes (IGDR), Université de Rennes 1, CNRS, UMR6290, 35065 Rennes, France; 11 MRC Mitochondrial Biology Unit, University of Cambridge, Cambridge, CB2 0XY UK; 12 Department of Molecular Genetics, University of Toronto, Toronto, ON M5S 3E1 Canada; 13 Bioinformatics and Computational Biology Graduate Program, University of Minnesota-Twin Cities, Minneapolis, MN 55455 USA; 14 RIKEN Centre for Sustainable Resource Science, Wako, Saitama, 351-0106 Japan; 15 Departmento de Ciências Médicas, Universidade de Aveiro, 3810−193 Aveiro, Portugal; 16 iBiMED - Institute of Biomedicine, Universidade de Aveiro, 3810−193 Aveiro, Portugal; 17 Department of Surgery, Haukeland University Hospital, N-5021 Bergen, Norway; 18 Present Address: Program in Genetics & Genome Biology, The Hospital for Sick Children, Toronto, ON M5G 1×8 Canada

**Keywords:** Acetyltransferases, Proteomics, Acetylation, Neddylation, Drosophila

## Abstract

Most eukaryotic proteins are N-terminally acetylated, but the functional impact on a global scale has remained obscure. Using genome-wide CRISPR knockout screens in human cells, we reveal a strong genetic dependency between a major N-terminal acetyltransferase and specific ubiquitin ligases. Biochemical analyses uncover that both the ubiquitin ligase complex UBR4-KCMF1 and the acetyltransferase NatC recognize proteins bearing an unacetylated N-terminal methionine followed by a hydrophobic residue. NatC KO-induced protein degradation and phenotypes are reversed by UBR knockdown, demonstrating the central cellular role of this interplay. We reveal that loss of *Drosophila* NatC is associated with male sterility, reduced longevity, and age-dependent loss of motility due to developmental muscle defects. Remarkably, muscle-specific overexpression of UbcE2M, one of the proteins targeted for NatC KO-mediated degradation, suppresses defects of NatC deletion. In conclusion, NatC-mediated N-terminal acetylation acts as a protective mechanism against protein degradation, which is relevant for increased longevity and motility.

## Abstract (web summary)

The most common protein modification in eukaryotes is N-terminal acetylation, but its functional impact has remained enigmatic. Here, the authors find that a key role for N-terminal acetylation is shielding proteins from ubiquitin ligase-mediated degradation, mediating motility and longevity.

## Introduction

The N-terminal protein landscape is a hotspot for modifications influencing protein interactions and homeostasis^1^. N-terminal acetylation (Nt-acetylation) is undoubtedly one of the most common protein modifications in eukaryotes, with the α-amino group of ~80–90% of human proteins being susceptible to acetylation by N-terminal acetyltransferases (NATs)^2,3^. Acetylation transforms a charged protein N-terminus into a hydrophobic segment, thereby potentially affecting key protein properties such as folding, polymerization, stability, interactions, and localization. Given the prevalence of Nt-acetylation, NATs regulate a wide range of cellular processes, ranging from metabolism, cell proliferation and migration to differentiation and stress response^4^. Dysregulation of Nt-acetylation can cause developmental disorders, by affecting brain and heart development, and contribute to cancer development^4–10^.

The human NAT family comprises five ribosome-associated members (NatA-NatE) that acetylate nascent polypeptides during translation^11^, as well as the Golgi-associated NAA60/NatF^12^ and the actin-specific NAA80/NatH^13,14^ which both act post-translationally. The NATs mainly recognize the first two amino acids at the N-terminus, but local sequence context may affect substrate binding and the degree of acetylation. NatA acetylates small N-terminal residues^2^, which are exposed after the initiator methionine has been removed by methionine aminopeptidases. Proteins harboring a N-terminal methionine can be modified by NatB/C/E/F. In this substrate class, NatB acetylates the α-amino group of methionine followed by an acidic or amidic residue^15^, while NatC, NatE and NatF acetylates the α-amino group of methionine followed by a hydrophobic or amphipathic residue^11,16,17^. Structural studies of NatC have shown that the first four amino acids contribute to substrate recognition^18–21^, and presumably NatC, NatE and NatF act on different substrates classes in vivo.

The evolutionarily conserved NatC complex consists of the catalytic subunit NAA30, the ribosomal anchor NAA35, and the small auxiliary subunit NAA38, all suggested to be required for normal enzymatic activity^20,22–25^. NatC-mediated acetylation increases the affinity of the two NEDD8-conjugating enzymes UBE2M/UBC12 and UBE2F to E3 ligases, promoting cullin neddylation^26,27^. Structural analysis showed that Ac-UBE2M is buried within a hydrophobic pocket of DCN1, which enhances cullin neddylation^27^, and this acetylation-dependent interaction can be antagonized by inhibitors^28^. Acetylation by NatC can be crucial for subcellular targeting, partly by mediating protein interactions. The GTPases ARL8B and ARFRP1 (Arl3 in yeast) rely on acetylation of their N-terminal methionine for correct targeting to the lysosomes and Golgi, respectively^24,29,30^. The latter example is driven by an interaction between the acetylated N-terminus and the membrane protein SYS1. Several studies have also linked NatC activity to development, stress response and longevity^31–34^, whereas the catalytic subunit of NatC, NAA30, was shown to regulate cancer cell viability and tumorigenesis of glioblastoma initiating cells^24,35,36^. Recently, a likely pathogenic *NAA30* variant was suggested to cause global developmental delay and tracheal cleft^9^.

The N-terminus of a protein can, depending on its nature, act as a degradation signal (N-degron) that is recognized by the N-degron pathways and targeted for degradation by the 26 S proteasome or lysosomes (via autophagy). In fact, most cellular proteins can be targeted for degradation by different N-degron pathways^37–40^. The Ac/N-degron pathway targets proteins harboring an acetylated N-terminal residue (mainly Met, Ala, Val, Ser, Thr and Cys) and is mediated by the E3 ubiquitin ligases Doa10 (MARCH6 in mammals) or Not4^41,42^. The Arg/N-degron pathway targets specific unacetylated N-terminal residues, termed type 1 (basic; Arg, Lys and His) and type 2 (bulky hydrophobic; Leu, Phe, Tyr, Trp, or Ile), that are exposed after proteolytic processing^40,43^. The Arg/N-degron pathway also recognizes unacetylated N-terminal methionine when followed by a bulky hydrophobic residue in yeast^44^. While the E3 ubiquitin ligase Ubr1 acts in the yeast Arg/N-degron pathway, in mammals there are at least four N-recognins, UBR1, UBR2, UBR4, and UBR5, whose individual contributions for protein degradation have not been extensively explored^45,46^. Many proteins are conditional N-degron substrates that are only available under certain circumstances. For example, natural Ac/N-degrons can be shielded within protein complexes^47^. Consequently, the N-degron pathways act as important control mechanisms to ensure protein quality and subunit stoichiometry.

Acetylation of protein N-termini underlies several key biological processes. Although NatC acetylates hundreds of different substrates, we know very little about the functional and physiological importance of these acetylation events. While the genes encoding the human NatA and NatB subunits are mostly essential^48–50^, human NatC knockout cell models enabled us to investigate the overall role of Nt-acetylation. In this study, we performed unbiased genome-wide CRISPR knockout screens in human HAP1 cells. We uncover a key role of Nt-acetylation in protecting hydrophobic N-termini against targeted degradation by the Arg/N-degron pathway. Moreover, NatC perturbation appears to regulate vesicle trafficking and organelle morphology. The organismal relevance of NatC was further investigated in *Drosophila melanogaster*. Altogether, our findings reveal how NatC-mediated acetylation promotes healthy proteostasis by regulating protein quality control and impacts motility among elderly and longevity in flies.

## Results

### Human NatC complex shows strong genetic interactions with the Arg/N-degron pathway

**Fig. 1:**
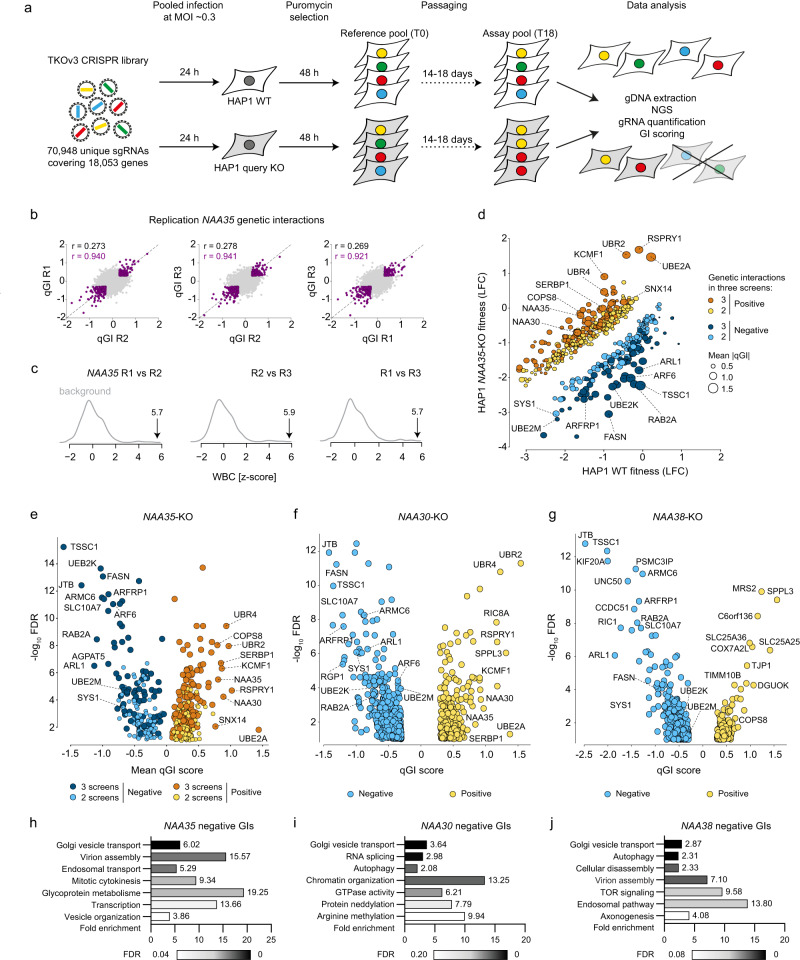
Genome-wide mapping of genetic interactions with human NatC. **a** Schematic of genome-wide CRISPR screens to identify genetic interactions (GI) with NatC. HAP1 WT, *NAA30*-KO, *NAA35*-KO, and *NAA38*-KO cells were transduced with a pooled genome-wide CRISPR knockout library (TKOv3) and selected for viral integration. gRNA regions were PCR-amplified from genomic DNA extracted from cells collected at the start (T0) and endpoint of the screen (T14-18). gRNA abundance was determined by next-generation sequencing (NGS). **b**, **c** Reproducibility of *NAA35* qGI scores. **b** qGI scores were determined by comparing the log_2_-fold change (LFC) for every gene represented in the TKOv3 library in *NAA35*-KO cell line with those observed in a panel of WT control screens. Pearson correlation coefficient (*r*) was calculated using all qGI scores (*r* in black, calculated from all data points) or using a stringent cut-off for the GIs (|qGI|>0.3, FDR < 0.10) in both screens (*r* and datapoints marked in purple). **c** The Pearson correlation coefficients of the qGI scores (two replicated screens) was adjusted to the similarity of a *NAA35*-KO screen to a panel of HAP1-KO screens. The resulting Within vs Between replicate Correlation (WBC) score provides a confidence of reproducibility interpreted as a z-score. **d** Negative and positive GIs of *NAA35*. Scatterplot showing the fitness effect (LFC) of 486 genes in *NAA35-*KO versus WT cells, showing a significant GI in at least two *NAA35* screens (|qGI|>0.3, FDR < 0.10). Negative (blue) and positive (yellow) *NAA35* GIs are shown. Darker color indicates interactions that were called in all three replicate screens. Node size corresponds to strength of the mean absolute GI score (three independent screens). Volcano plots displaying qGI scores and associated significance (log_10_ values) for genes targeted by the TKOv3 library in (**e**) *NAA35-*KO, (**f**) *NAA30*-KO and (**g**) *NAA38*-KO screens. Negative (blue) and positive (yellow) GIs are shown. **h**–**j** Negative GIs of NatC indicate a role in Golgi transport. Pathway enrichment analysis of genes exhibiting a negative GI with (**h**) *NAA35*, (**i**) *NAA30* or (**j**) *NAA38* (identified in at least two *NAA35* screens; |qGI|>0.3, FDR < 0.1). Benjamini–Hochberg adjusted p-values for each gene ontology term is indicated by gray gradient.

Genetic interactions (GIs) occur when the combination of mutations in different genes leads to an unexpected phenotype, considering the effects of the individual gene mutations, and can reveal functional relationships between genes and pathways^51,52^. Mapping GI profiles has thus become a powerful approach for deciphering gene function. To systematically identify GIs for NatC, we performed genome-wide CRISPR knockout (KO) screens in human HAP1 WT and *NAA30*-KO, *NAA35*-KO or *NAA38*-KO cell lines (collectively referred to as NatC KO) (Supplementary Fig. 1) using the TKOv3 guide RNA (gRNA) library targeting ~18,000 protein-coding genes (Fig. 1a). The relative abundance of a specific gRNA between the start and end timepoint of a screen provides an estimate of single mutant fitness in WT cells and double-mutant fitness in KO cells. We have previously developed a quantitative GI (qGI) score that compares the double-mutant fitness effects in a query KO cell line with the single-mutant fitness effects in a panel of HAP1 WT control screens and corrects for various experimental artifacts^53^. In this context, a negative GI occurs when the simultaneous knockout of two genes leads to a more severe cell fitness decrease than expected by considering the individual effects of both genes. In our screen, this is identified by reduced gRNA abundance in the KO cell line relative to the WT control cell line. Conversely, a positive GI occurs when the combined disruption of two genes promotes cell fitness, and results in increased gRNA abundance in the KO cells compared to WT cells.

We performed three independent screens using a *NAA35*-KO query cell line to establish a high-confidence GI dataset for the major NatC auxiliary subunit. The reproducibility of the double-mutant fitness effects (log_2_-fold change, LFC) between the three *NAA35* screens were strongly correlated (pairwise *r* = 0.81) (Supplementary Figs. 2–4). Furthermore, we observed only a modest correlation between the genome-wide qGI scores of the *NAA35* replicate screens (*r* = 0.27–0.28) (Fig. 1b), which was expected given the relative sparsity of GIs^53^. When restricting our correlation analysis to significant GIs in pairs of *NAA35* replicate screens (|qGI|>0.30; FDR < 0.10) the pairwise correlation increased considerably (*r* = 0.92–0.94), suggesting that significant GIs were reproducible. Indeed, the qGI scores measured in the *NAA35* screens were highly reproducible with a Within vs Between Correlation (WBC) score (adjusted z-score) between 5.7 and 5.9 (Fig. 1c)^54^.

We next generated a combined set of *NAA35* GIs by mean-summarizing the qGI scores of the three replicate screens (Fig. 1d, e). Negative GIs with *NAA35* included *TSSC1/EIPR1*, *JTB*, *ARL1*, *RAB2A*, *FASN*, *SLC10A7*, *ARF6* and *ARFRP1* possibly reflecting NatC’s impact on the secretory pathway and organelles^16,24,55^. Indeed, negative *NAA35* GIs were enriched for genes involved in Golgi vesicle transport, virion assembly, and endosomal transport (FDR < 0.20) (Fig. 1h, Supplementary Data 2). The role of NatC in vesicle trafficking is most likely evolutionarily conserved as many of the same NatC GIs were observed in the global yeast genetic network (*ARL1*, *SYS1*, *ARFRP1*, *RIC1/KIAA1432*, *RGP1*, *COG5*, and *COG7*)^51,56^. Arl3/ARFRP1 is a small GTPase that recruits Arl1 and its effectors to the *trans*-Golgi. The targeting of Arl3 to the Golgi requires NatC-mediated acetylation and the membrane protein Sys1^29,30^. COG5 and COG7 are components of the conserved oligomeric Golgi (COG) complex, which acts in *intra*-Golgi protein transport^57^. We also observed that several members of the Rab family of small GTPases (*RAB1A*, *RAB1B*, *RAB2A*, *RAB14*) regulating membrane trafficking^58^ share negative GIs with *NAA35*, emphasizing the role of NatC in intracellular transport. The strongest positive qGI scores were also highly reproducible and included components of the Arg/N-degron pathway UBE2A, UBR2, UBR4, KCMF1, a component of the COP9 signalosome promoting cullin deneddylation COPS8, as well as the NatC genes NAA30 and NAA35 confirming the responsiveness of the screen. Moreover, NAA35 positive GIs were enriched for genes with annotated roles in ribosome biogenesis, RNA processing and translation (Supplementary Data 2), suggesting that loss of NatC might be buffered by translational perturbations.

To better understand the genetic dependencies of the NatC complex, we next performed genome-wide CRISPR KO screens in *NAA30*-KO and *NAA38*-KO cell lines (Fig. 1f, g, Supplementary Data 1). Each query screen was performed once with technical triplicates, and we used the same confidence threshold as for the *NAA35* screens (|qGI|>0.30, FDR < 0.10) (Supplementary Figs. 3 and 4). Notably, both *NAA30* and *NAA38* negative GI profiles revealed genes involved in Golgi vesicle transport (Fig. 1i, j). We next compared the NatC KO screens. Overall, 63 and 65 genes in the *NAA30* screen also significantly interacted with *NAA35* in 2 or 3 screens, respectively (128 genes in total). The *NAA38* GI profile contains 137 and 75 genes that also significantly interacted with *NAA30* and *NAA35* in at least 2 screens, respectively. We observed that the positive GIs were less shared between *NAA30*, *NAA35*, and *NAA38*, most likely caused by the *NAA38*-KO cells having increased proliferation rate compared to the other cell lines (Supplementary Fig. 5). Taken together, the negative GIs of NatC suggest cellular roles related to the secretory pathway and organellar biology, while the positive GIs propose a functional link to the N-degron pathway and proteostasis.

### NAA30, NAA35, and NAA38 are essential for cellular Nt-acetylation and proteostasis of NatC-type substrates

**Fig. 2:**
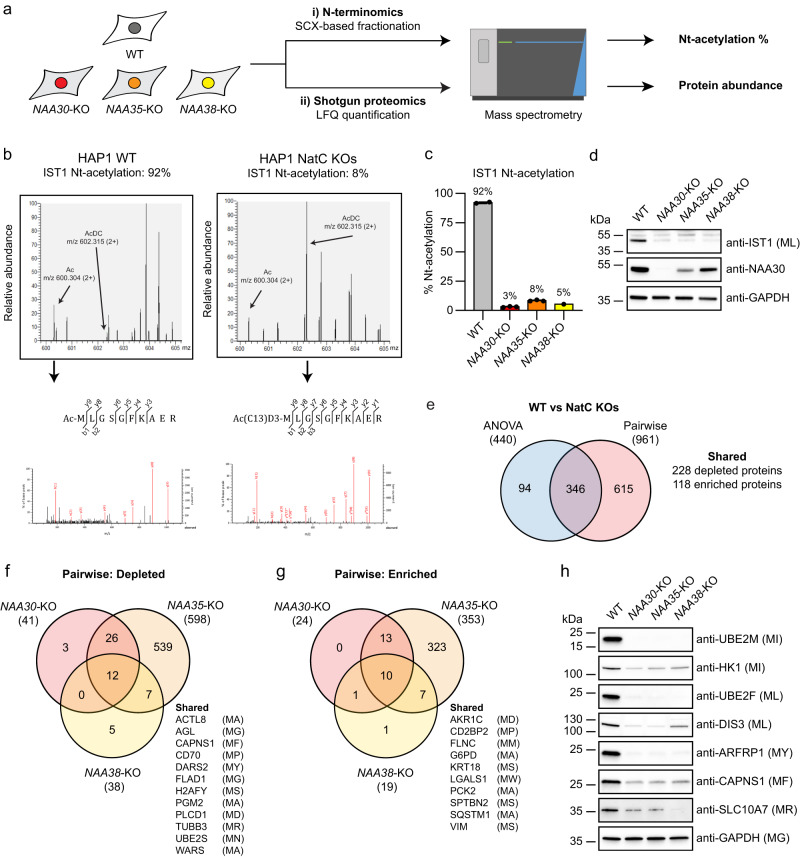
N-terminal acetylation by NatC protects proteins from degradation. **a** HAP1 WT and NatC KO cells were subjected to i) N-terminal proteomics using strong-cation exchange (SCX) enrichment of in vivo Nt-acetylated proteins to determine the degree of Nt-acetylation, and ii) label-free quantification (LFQ) shotgun proteomics to determine changes in protein abundance (four samples per cell line). **b** MS-spectra of the N-terminal peptide of IST1 (P53990) from HAP1 WT and NatC KO cells following trypsin digestion and SCX-based enrichment. **c** Bar graph showing the degree of Nt-acetylation of IST1 in HAP1 WT and NatC KO cells as determined by proteomics. Data are shown as mean ± SD (*n* = 4, the IST1 peptide was not identified in all replicates). **d** Endogenous IST1 protein levels from indicated HAP1 cells determined by immunoblotting. **e** Venn diagram of significantly regulated proteins in HAP1 NatC KO cell lines determined by LFQ proteomics using multiple sample test (one-way ANOVA; FDR = 0.01, S0 = 0) and pairwise comparison analyses (t-test; FDR = 0.01, S0 = 0.1). **f**, **g** Venn diagrams of depleted (**e**) and enriched (**f**) proteins determined by pairwise comparison between HAP1 WT and the individual NatC KO cell lines (t-test; FDR = 0.01; S0 = 0.1). **h** Immunoblot analysis of selected confirmed and putative NatC substrates using whole cell extract from HAP1 WT and NatC KO cells. Immunoblots were performed at least three independent times. Source data are provided as a Source Data file.

To explore the dependencies of the NAA30, NAA35, and NAA38 subunits for NatC-mediated Nt-acetylation in human cells, we performed N-terminal proteomics using the same cell models (Fig. 2a). The N-terminal acetylome profile was achieved by digesting the proteomes with trypsin, followed by in vitro acetylation of unprotected N-termini with heavy acetyl, and finally enrichment of N-terminal peptides by strong cation exchange (SCX)^59^. The degree of in vivo Nt-acetylation was determined using MASCOT Distiller (see Methods). All three subunits were important for Nt-acetylation of different NatC-type substrates, including the protein IST1 harboring a MLGS- starting N-terminus (Fig. 2b, c, Supplementary Data 3). While IST1 was >90% Nt-acetylated in WT cells, it was <10% Nt-acetylated in all three NatC KO cell lines. Also, endogenous IST1 protein levels were significantly reduced in all NatC KO cell lines as compared to WT cells (Fig. 2d). For several other proteins, such as the previously defined NatC substrates UBE2M (MI-)^27^ and ATXN2L (ML-)^16^ and the putative substrates DIS3 (ML-), ADNP (MF-), ADNP2 (MF-) and ITM2A (MV-), the Nt-acetylation levels could only be determined in WT cells and not in NatC KO cells, very likely indicating that these proteins were less abundant (Table 1 and Supplementary Data 3). To further address this issue, we performed label-free quantitative (LFQ) proteomics on HAP1 WT and NatC KO cells. We identified 440 proteins that were differentially expressed between WT and NatC KOs using multiple ANOVA testing (log_2_ transformed LFQ values, FDR = 0.01, S0 = 0) (Supplementary Data 4). A pairwise comparison between the individual query KO cell lines with the WT control cell line identified 961 proteins that were differentially expressed (two-sample *t* test, FDR = 0.01, S0 = 0.1). 346 of the ANOVA significant proteins were also significant in at least one pairwise comparison (Fig. 2e), of which 66 % were depleted while 34% were enriched compared to WT (based on FDR = 0.01 values). Notably, the NatC KO cell lines had different effects on the proteome and most deregulated proteins were identified in the *NAA35*-KO cells. However, most deregulated proteins that were identified in the *NAA30*-KO and *NAA38*-KO cells were also identified in the *NAA35*-KO cells, indicating a shared NatC effect (Fig. 2f, g). In total, 12 depleted and 10 enriched proteins were shared among the NatC KO cells, of which CAPNS1 (MF-), DARS (MY-), and FLNC (MM-) have NatC/E/F-type N-termini (Supplementary Data 4). We further uncovered that several proteins with NatC/E/F type N-termini, including hnRNP F (MLGP-)^18^, were fully acetylated in HAP1 WT and NatC KOs indicating that these are likely NatE (or NatF substrates) or that one of these NATs likely rescue their Nt-acetylation status in the absence of NatC (Supplementary Data 3).

**Table 1:** Human NatC substrates identified in HAP1 cells using SCX-based N-terminal proteomics

Protein	UniProt ID	P1'	P2'	Start	Modification^a^	Detected peptide	Median Nt-acetylation (%)^b^				Protein abundance WT vs NatC KO^c^
							WT	*30*-KO	*35*-KO	*38-*KO	
SSBP3	Q9BWW4	M	F	1	Ac	MFAKGKG..	99.4	-	-	-	nd
					AcDC		-	-	9.1	-	
EBF3	Q9H4W6	M	F	1	Ac	MFGIQEN..	100.0	-	-	-	nd
NUP133	Q8WUM0	M	F	1	Ac	MFPAAP..	99.9	-	-	-	Depleted
ADNP2	Q6IQ32	M	F	1	Ac	MFQIPVE	98.9	-	-	-	nd
ADNP	Q9H2P0	M	F	1	Ac	MFQLPVN..	99.8	-	-	-	nd
RPS7	P62081	M	F	1	Ac	MFSSSAK..	76.9	-	-	-	nd
					AcDC		77.3	25.0	-	22.3	
UBE2M	P61081	M	I	1	Ac	MIKLFSL..	99.0	-	-	-	Depleted
DPYSL5	Q9BPU6	M	L	1	Ac	MLANSA..	98.8	-	-	-	Depleted
IST1	P53990	M	L	1	Ac	MLGSGFK..	92.1	-	-	-	nd
					AcDC		-	3.1	8.1	5.3	
ATXN2L	Q8WWM7	M	L	1	Ac	MLKPQPL..	99.5	-	-	-	Enriched
DIS3	Q9Y2L1	M	L	1	Ac	MLKSKTF..	99.1	-	-	-	Depleted
MAP7D3	Q8IWC1	M	M	1	Ac	MMADGAA..	99.7	-	-	-	nd
ANK2	Q01484	M	M	1	Ac	MMNEDAA..	100.0	-	-	-	Depleted
ITM2A	O43736	M M	V V	1 2	Ac AcDC	MVKIAFN.. VKIAFNT..	99.0 0.1	- 0.7	- -	- 0.1	nd
SSBP4	Q9BWG4	M	Y	1	Ac	MYAKGGK..	99.4				nd
MCRIP2	Q9BUT9	M	Y	1	Ac AcDC	MYTITKG..	99.9 -	- 34.5	- -	37.5 34.5	nd

*nd* not detected.

^a^In vivo (Ac) and in vitro (AcDC) acetylated N-termini.

^b^Median Nt-acetylation percentage in HAP1 WT, *NAA30*-KO, *NAA35*-KO, *NAA38*-KO cells is indicated (*n* = 4, Supplementary Data 3).

^c^Change in protein abundance between WT and at least one NatC KO cell line determined by LFQ proteomics (Supplementary Data 4).

Based on the genetic and proteomics data, we investigated a series of potentially regulated NatC substrates by immunoblot analysis using lysates from WT cells and the different NatC KO cells. All three HAP1 NatC KO cell lines displayed downregulation of the NEDD8-conjugating enzymes UBE2M and UBE2F, DIS3, HK1, CAPNS1, SLC10A7, and the confirmed NatC substrate ARFRP1 (Fig. 2h)^29,55^. We further confirmed reduced protein levels of selected NatC substrates in an independent *NAA30*-KO breast cancer cell line (Supplementary Fig. 6). Altogether, this suggests that these proteins are likely substrates of the trimeric NatC complex and depend on NatC for their stability.

### N-terminal sequence and acetylation status are determinants of protein degradation

**Fig. 3:**
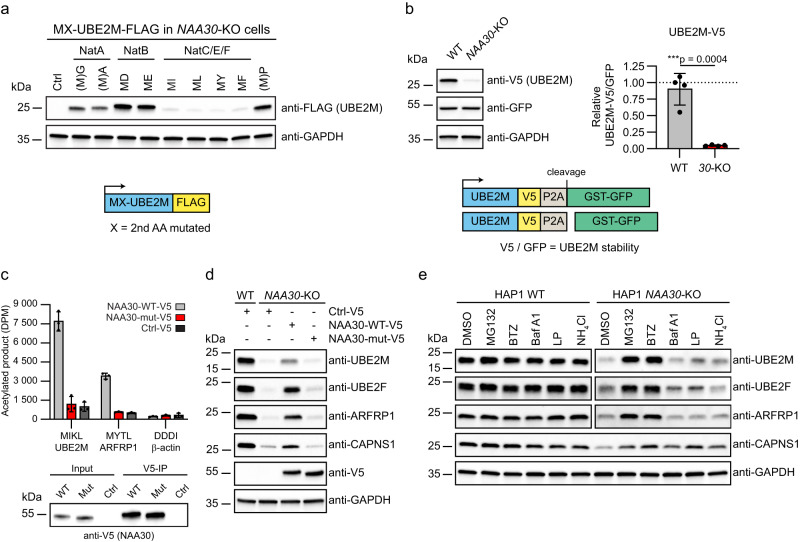
Unacetylated hydrophobic NatC substrates are less stable. **a** N-terminal variants of UBE2M-FLAG were transiently expressed in HAP1 *NAA30*-KO cells and protein levels were determined by immunoblotting (*n* = 3 independent experiments). The native N-terminus of UBE2M starts with MI. **b** HAP1 WT and *NAA30-*KO cells were transfected with the indicated UBE2M-V5-P2A-GST-GFP reporter construct, and protein levels were determined by immunoblot analysis. UBE2M-V5 levels were normalized to GST-GFP and expressed relative to WT sample. Data are shown as mean ± SD of four independent experiments. ***p = 0.0004; two-tailed unpaired *t* test. **c** NAA30-WT-V5 and NAA30-mut-V5 (E321A) was immunoprecipitated from HeLa cell extracts and used in Nt-acetylation assays with [^14^C]-acetyl-CoA and synthetic peptides representing the NatC substrates UBE2M (MIKL) and ARFRP1 (MYTL), and the NAA80/NatH substrate β-actin (DDDI). The experiment was performed three independent times with three technical replicates each. Data from one representative setup is shown as mean ± SD. DPM: disintegrations per minute. **d** NatC regulates the protein level of UBE2M, UBE2F, ARFRP1, and CAPNS1. Immunoblot analysis of HAP1 WT and *NAA30*-KO cells transfected with control V5 plasmid, NAA30-V5 or the catalytically dead mutant NAA30-mut-V5 (*n* = 3 biologically independent samples). **e** HAP1 WT and *NAA30*-KO cells were treated with proteasomal [MG132 and bortezomib (BMZ)] and lysosomal inhibitors [bafilomycin A (BafA), leupeptin (LP) or ammonium chloride (NH_4_Cl)] for 6 h followed by immunoblot analysis using the indicated antibodies (*n* = 3 independent experiments). DMSO served as vehicle control. Source data are provided as a Source Data file.

To define a potential Nt-sequence dependency of the NatC KO induced protein level regulation, we expressed N-terminal variants of UBE2M-FLAG in HAP1 *NAA30*-KO cells and determined protein levels by immunoblotting. In these cells, the UBE2M WT protein (MI-) and other variants with NatC-type N-termini (ML-, MY-, MF-) were expressed at very low levels, while variants with N-termini representing lack of Nt-acetylation and lack of methionine (MP-, which is processed to P-), or NatA (MG-, MA-) or NatB (MD-, ME-) substrate N-termini, were present at higher levels suggesting that these provide a stabilizing effect (Fig. 3a). To exclude any effects at the transcriptional or translational levels, HAP1 WT and *NAA30*-KO cells were transfected with a bicistronic P2A reporter vector encoding UBE2M-V5-P2A-GST-GFP. Normalized UBE2M-V5 protein levels were significantly lower in *NAA30*-KO cells compared to WT cells (Fig. 3b). The observed effects of NatC KO on several proteins are likely driven by the direct lack of NatC-mediated Nt-acetylation. To exclude the involvement of non-catalytic roles of NatC subunits, we generated a catalytically dead mutant of NAA30 based on sequence analysis of evolutionarily conserved amino acid residues crucial for catalysis (Supplementary Fig. 7a)^18,20^. While NAA30-WT was capable of Nt-acetylating both UBE2M and ARFRP1-derived synthetic peptides, as shown by an in vitro acetylation assay, the mutant NAA30-mut (E321A) was not, despite being normally expressed (Fig. 3c). In *NAA30-*KO cells, re-expression of NAA30-WT, but not NAA30-mut was able to partially restore protein levels of UBE2M, UBE2F, ARFRP1 and CAPNS1 (Fig. 3d). This confirms that the observed effects are NAA30-specific and that the catalytic activity of NAA30 is essential for the regulation of these proteins. To confirm that the NatC-mediated protein regulation is at the level of protein degradation, HAP1 WT and *NAA30*-KO cells were treated with proteasomal (MG132 and bortezomib) and lysosomal (bafilomycin A, and leupeptin) inhibitors. Immunoblot analyses of cell lysates clearly demonstrated that UBE2M, UBE2F and ARFRP1 were all degraded via the proteasome in *NAA30*-KO cells while CAPNS1 might be degraded both via proteasomal and lysosomal degradation in *NAA30*-KO cells (Fig. 3e). Altogether, these data strongly suggest that NatC-mediated Nt-acetylation may directly and positively steer protein stability of its substrates.

### N-recognin UBR4-KCMF1 targets non-Nt-acetylated NatC substrates for degradation

**Fig. 4:**
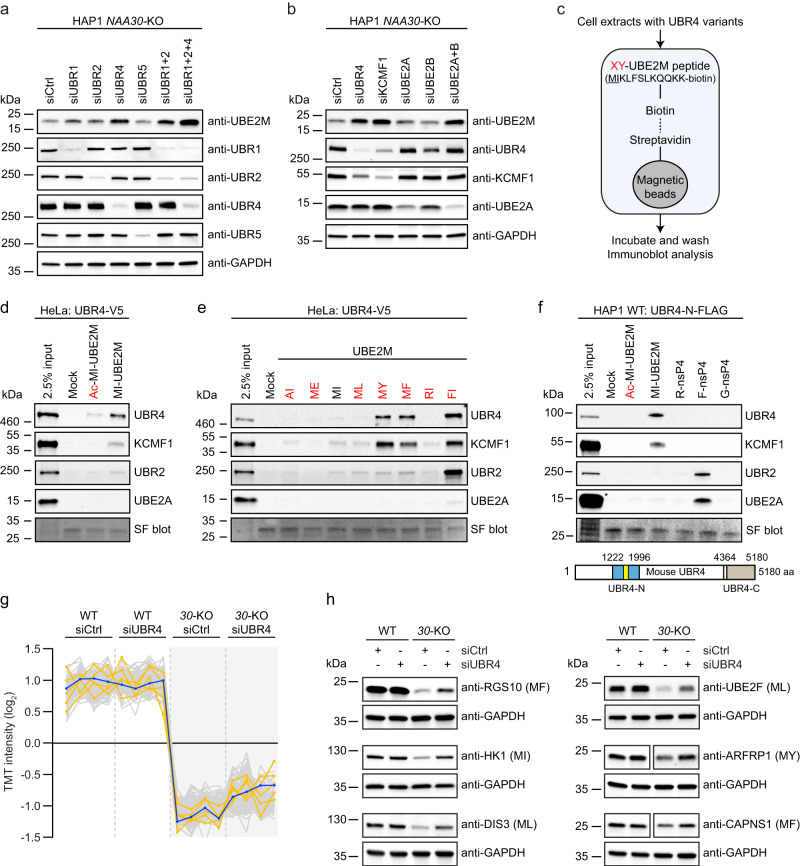
UBR4-KCMF1 targets unacetylated N-terminal methionine followed by a hydrophobic residue. **a**, **b** The protein level of endogenous UBE2M in HAP1 WT and *NAA30*-KO cells transfected with the indicated siRNAs for 72 h was assessed by immunoblotting (*n* = 4 biologically independent samples). **c** Schematic representation of protein capture by peptide pulldown. A set of 11-mer peptides derived from the N-terminal sequence of UBE2M were C-terminally labeled with K-biotin (MIKLFSLKQQK(K-biotin)) and conjugated to streptavidin magnetic beads. The first two residues were replaced to represent different N-termini (XY-UBE2M). Biotinylated UBE2M peptides were incubated with cell extracts and the pulled-down proteins were identified by immunoblot analysis. **d** In vitro peptide pulldown assay of UBR4-V5 transiently expressed in HeLa cells using acetylated and non-Nt-acetylated UBE2M peptide. **e** XY-UBE2M peptide pulldown assay with UBE2M peptides bearing different N-terminal amino acids and UBR4-V5 expressed in HeLa cells. **f** In vitro peptide pulldown assay of UBR4-N-FLAG expressed in HAP1 WT cells using acetylated and non-Nt-acetylated UBE2M peptide and X-nsP4 controls peptides. The UBR4-N construct contains the UBR-box (yellow), which is the substrate recognition domain of the UBR proteins. *indicates saturated UBE2A band. **g** HAP1 WT and *NAA30-*KO cells were transfected with siCtrl or siUBR4 for 72 h and protein abundance was determined by tandem mass tag (TMT)-based quantitative proteomics (see Supplementary Data 5**;** *n* = 4 biologically independent samples). Intensity profile plot showing protein levels of the top 100 proteins with abundance profiles most similar to UBE2M (blue trace) (one-way ANOVA, permutation-based FDR = 0.01, S0 = 0). The intensity profiles of RGS10, ARLB, CAPSN1, DIS3, and HK2 are highlighted in orange. **h** UBR4 knockdown stabilizes the protein levels of non-Nt-acetylated RGS10, HK1, DIS3, UBE2F, ARFRP1 and CAPNS1 in *NAA30*-KO cells. HAP1 WT and *NAA30*-KO cells were transfected with siCtrl or siUBR4 for 72 h followed by immunoblotting using the indicated antibodies (**d**–**f** and **h**; *n* = 3 independent experiments). Source data are provided as a Source Data file.

To elucidate how non-Nt-acetylated NatC substrates are targeted for degradation, we perturbed various UBR-box E3 ligases, called N-recognins, which recognizes different types of protein N-termini^45,46^ and examined the endogenous UBE2M protein levels by immunoblotting. Knockdown of UBR4, and to some extent also knockdown of UBR1 and UBR2, increased the protein levels of UBE2M in *NAA30*-KO cells (Fig. 4a). A combination of UBR1 and UBR2 knockdown, and in particular a triple knockdown of UBR1, UBR2 and UBR4 strongly raised UBE2M protein levels. This combined effect is consistent with the finding that these UBRs have partially overlapping targets^39,46^ The E3 ligase UBR4 physically interacts with the E3 KCMF1 and the E2 UBE2A (RAD6)^60^, suggesting that they form a ubiquitin ligase complex. Both UBR4, KCMF1 and UBE2A were identified as positive GIs of NatC (Fig. 1d). To assess whether the UBE2A-KCMF1-UBR4 complex is responsible for targeting non-Nt-acetylated UBE2M for degradation, we separately knocked down KCMF1 as well as UBE2A and its human paralogue UBE2B. Indeed, UBE2M protein levels were restored when depleting human cells for UBR4, KCMF1 or by the simultaneous knockdown of UBE2A and UBE2B (Fig. 4b). In agreement with the lack of Nt-acetylation as a determinant for UBR4-KCMF1-mediated UBE2M degradation, we were not able to observe any changes in UBE2M protein level when knocking down UBR4 or KCMF1 in HAP1 WT cells where NatC Nt-acetylation is intact (Supplementary Fig. 8a). Moreover, the knockdown experiments revealed mutually interdependent protein levels of UBR4 and KCMF1. Finally, the protein levels of the top positive GIs were also analyzed, to rule out differential activity in NatC KO cells relative to WT cells, and except from the putative NatC substrate RSPRY1 (MI-) none of these appeared to be markedly shifted in NatC KO cells (Supplementary Fig. 8b).

To directly assess the Nt-acetylation dependency of UBR4-KCMF1-mediated protein targeting, we performed a peptide pulldown assay using a 11-mer peptide derived from the N-terminal sequence of UBE2M and HeLa cells transiently transfected with full-length UBR4-V5 (Fig. 4c). The non-Nt-acetylated UBE2M (MI-UBE2M) pulled out UBR4-V5, KCMF1 and UBR2 from cell lysates to a larger extent than Nt-acetylated UBE2M (Ac-MI-UBE2M) (Fig. 4d). Also, all the non-Nt-acetylated UBE2M peptides with different NatC-type N-terminal sequences (MI/ML/MY/MF-UBE2M) pulled out UBR4-V5, KCMF1 and UBR2, while UBE2M peptides with a NatA-type (AI-UBE2M) or NatB-type N-terminus (ME-UBE2M) were impaired in this capacity (Fig. 4e). Control UBE2M peptides bearing the classical Arg/N-degron N-terminal residues Arg (type 1) (RI-UBE2M) or Phe (type 2) (FI-UBE2M)^45^ weakly and strongly captured these cellular E2-E3 components, respectively.

The UBR box is the substrate recognition domain of UBR1 and UBR2, and recognizes type 1 N-degrons (basic), while an additional segment, termed the N-domain, is required for type 2 (bulky hydrophobic) recognition^46^. The UBR box of UBR4 most likely assumes the same fold as UBR1 and UBR2, but lacks a critical coordination residue and may therefore require additional structural elements for substrate recognition^61^. To further define the UBE2M-UBR4 interaction, we tested whether an N-terminal segment of mouse UBR4 containing the UBR box (UBR4-N) had the ability to bind to non-Nt-acetylated UBE2M peptide. We found that UBR4-N transiently expressed in HAP1 WT cells was specifically pulled out together with KCMF1 by non-Nt-acetylated UBE2M, but not by Nt-acetylated UBE2M (Fig. 4f). In this case, the Phe-starting control peptide (F-nsP4), derived from a Sindbis virus polymerase^45,62^, pulled out UBR2, but not UBR4-N and KCMF1, while Arg- and Gly-starting control peptides (R-nsP4 and G-nsP4) did not pull out any of these components of the N-degron pathway. Furthermore, none of the peptides were able to pull out a C-terminal segment of UBR4 (data not shown) considered to be sufficient for ubiquitin ligation^63,64^. Taken together, our data suggests that N-termini of NatC-type substrates (MI, ML, MY, MF) are recognized by UBR4, KCMF1 and UBR2 when present in their non-Nt-acetylated state.

### Loss of UBR4 protects unacetylated hydrophobic N-termini from degradation

We have already shown that the stability of several proteins depends on the NatC complex and its catalytic activity (Figs. 2–3), and that *UBR4* knockdown can reverse this effect for UBE2M in *NAA30*-KO cells (Fig. 4a). To assess the potential interplay between NatC and UBR4-mediated protein degradation on a global level, we performed *UBR4* siRNA knockdown in HAP1 WT and in *NAA30*-KO cells followed by TMT-based quantitative proteomics. Several proteins were regulated depending on NatC and/or UBR4 status (Supplementary Fig. 8c and Supplementary Data 5). We identified proteins with abundance profiles similar to UBE2M: displaying reduced protein levels in *NAA30-*KO as compared to WT cells and additionally increased protein levels in siUBR4-treated compared to siCtrl-treated *NAA30*-KO cells (Fig. 4g) (FDR = 0.01, S0 = 0.1). Regulator of G protein signaling (RGS) proteins play crucial regulatory roles in G-protein-mediated signal transduction, and RGS2, RGS4, RGS5 and RGS16 are known in vivo substrates of the Arg/N-degron pathway^65–67^. Interestingly, we found that RGS10 (MF-) has the most similar abundance profile compared to UBE2M, indicating that yet another RGS protein might be targeted by the N-degron pathway. We also identified the confirmed NatC substrate ARL8B^24,68^ and several other proteins thought to be regulated by NatC, such as CAPNS1, HK1/2, and DIS3. These putative NatC substrates proteins were independently verified by immunoblot analyses to be negatively regulated by *NAA30-*KO and positively regulated by *UBR4*-knockdown (Fig. 4h). Altogether, our data suggests that many NatC substrates are shielded from UBR4-targeted degradation via Nt-acetylation.

### The principal molecular role for NatC-mediated Nt-acetylation is blocking protein degradation occurring via the N-degron pathway

**Fig. 5:**
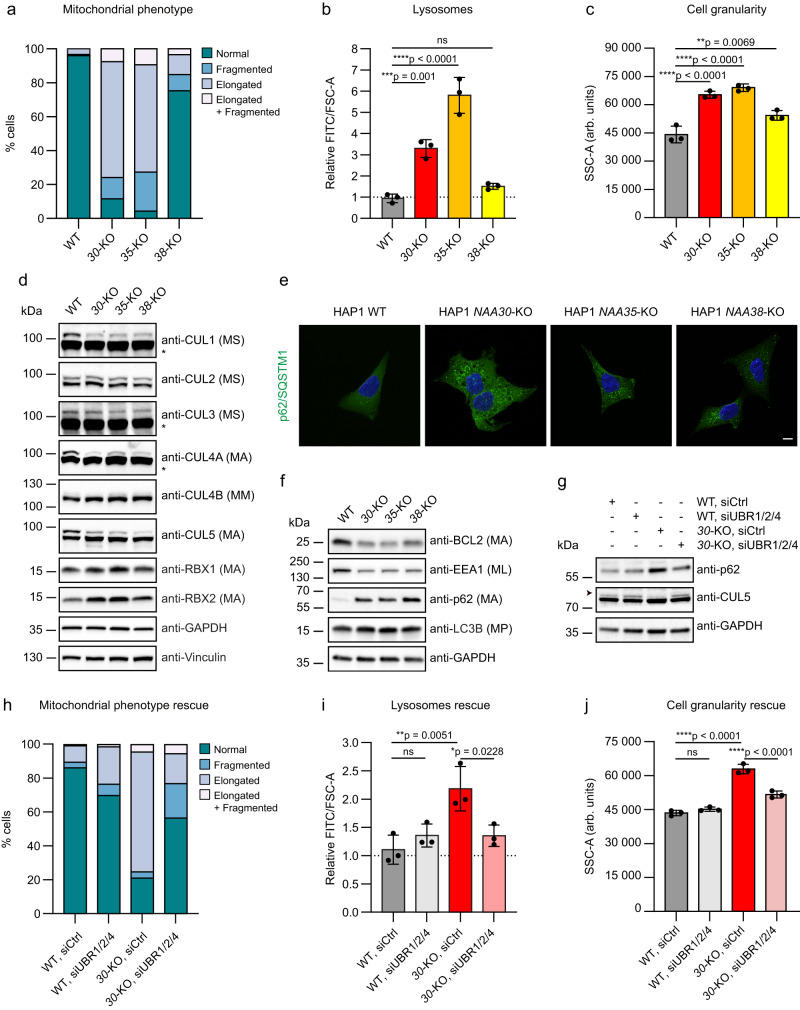
UBR-dependent NatC knockout phenotypes include abnormal mitochondrial morphology, increased lysosomal content and cell granularity. **a** NatC KO cells have abnormal mitochondrial morphology. HAP1 cells were stained with anti-COX IV and analyzed by immunofluorescence (IF). Cells were grouped into four bins based on mitochondrial morphology: normal, fragmented, elongated, and elongated + fragmented (*n* = 100 per cell line). **b** *NAA30*-KO and *NAA35*-KO cells have increased lysosomal content. Lysosomes were stained with LysoView 488 and analyzed by flow cytometry. Median FITC values were normalized to cell size (FSC-A) and expressed relative to WT sample. Data are shown as mean ± SD (*n* = 3 independent experiments). ***p = 0.001, ****p < 0.0001; one-way ANOVA with Dunnett’s correction. **c** NatC KO cells display increased granularity. Median side scatter area (SSC-A) indicating cell granularity or internal complexity was determined by flow cytometry. Data are shown as mean ± SD (*n* = 3 independent experiments). **p = 0.0069, ****p < 0.0001; one-way ANOVA with Dunnett’s correction. **d** NatC regulates protein neddylation. Immunoblot analysis of neddylation pathway components using total cell extract (*n* = 3 biologically independent samples). All proteins have NatA-type N-termini, except CUL4B which has a NatC/E-type N-terminus (MM-). The upper cullin band represents the neddylated form and * indicates saturated bands. **e** Increased p62/SQSTM1 levels in NatC KO cells. HAP1 cells were stained with anti-p62 and analyzed by IF. Scale bar, 10 μm. (see Supplementary Fig. 10 for quantification): **f** NatC affects components of the autophagy pathway. Immunoblot analysis of autophagy markers using the indicated HAP1 cell extracts. EEA1 (ML-) has a NatC-type N-terminus while BCL2, p62 and LC3B has NatA-type N-termini (*n* = 3 biologically independent samples). **g**–**j** HAP1 WT and *NAA30*-KO cells were transfected with siCtrl or siUBR1/UBR2/UBR4 for 72 h (*n* = 3 independent experiments). **g** Immunoblot analysis of endogenous p62 and CUL5 protein levels. Arrowhead indicates NEDD8-CUL5. **h** Mitochondrial morphology was assessed by IF like in a. **i** Lysosome levels and **j** cell granularity (SSC-A) was determined by flow cytometry as in b and c, respectively (**f**, **g**). **i**, **j** Data are shown as mean ± SD (*n* = 3 independent experiments). *p = 0.0228, **p = 0.0051, ****p < 0.0001; one-way ANOVA with Šídák’s correction. ns not significant. Source data are provided as a Source Data file.

Our finding that several components of the Arg/N-degron pathway are strong positive GIs of the NatC subunits (Fig. 1d–f), suggests that removal of this protein degradation system may eliminate NatC KO-mediated negative effects on cell viability. Furthermore, our data show that several cellular proteins are regulated, in a Nt-acetylation and sequence dependent manner by NatC and N-recognins (Figs. 2 and 3, Supplementary Data 3-4). Based on (i) Nt-acetylome analyses, (ii) the substrate specificity of human NatC, and (iii) proteome-wide estimations, up to 20% of the human proteome might be Nt-acetylated by NatC^3,11,16^. This plethora of NatC substrates, many of which might need Nt-acetylation for stability, may imply that multiple cellular functions and pathways are regulated by the NatC complex. Previous studies have shown that the NatC complex has an important role in ensuring correct mitochondrial and Golgi function^16,23,55^. Indeed, we found that NatC KO cells displayed notable abnormalities in organelles and in the secretory pathway: including aberrant mitochondrial morphology (Fig. 5a and Supplementary Fig. 9a) and increased lysosomal content (Fig. 5b). Organellar abnormalities were reflected in increased cellular granularity for all NatC KO cell lines (Fig. 5c) and increased cellular dehydrogenase activity (Supplementary Fig. 9b). Furthermore, we found that NatC-mediated Nt-acetylation is important for UBE2M/UBE2F-mediated cullin neddylation. CUL1, CUL4A and CUL5 were less neddylated in all NatC KO cell lines (Fig. 5d) (the upper band represents neddylated cullins). In addition, the NatC KO cells had increased levels of the NEDD8 ligase RBX2/RNF7, but the same effect was not observed for RBX1. Our proteomics analysis suggested that the autophagy receptor p62/SQSTM1 was upregulated in NatC KO cells (Supplementary Data 4), which was corroborated at the single-cell level by immunofluorescence analysis (Fig. 5e and quantified in Supplementary Fig. 10). Finally, we found by immunoblotting that the autophagy regulator BCL2 and the early endosome marker EEA1 were downregulated, while p62 was upregulated but without an apparent activation of autophagy via LC3B detection (Fig. 5f). The effect on EEA1 and p62 was confirmed in an independent *NAA30*-KO breast cancer cell line (Supplementary Fig. 6). It has previously been shown that p62 can be increased independent of autophagy for instance related to mitochondrial stress and mitochondria clustering independent of mitophagy^69,70^. We next wanted to define whether NatC’s ability to shield proteins from N-degron mediated protein degradation represents a general cellular NatC function. We depleted *NAA30*-KO cells for UBR1, UBR2, and UBR4 due to their overlapping effect on NatC substrates (Fig. 4a). UBR knockdown fully or partially restored all tested cellular NatC KO phenotypes including increased p62, decreased CUL5 neddylation (Fig. 5g), abnormal mitochondria morphology (Figu. 5 h), elevated presence of lysosomes (Fig. 5i), and increased cellular granularity (Fig. 5J). In summary, the combination of genetics data (Fig. 1), cellular data (Figs. 2–4), biochemical data (Fig. 4), and phenotype data (Fig. 5) show that the major molecular role of (NatC-mediated) Nt-acetylation is shielding proteins from the N-degron pathway.

### Identification of the *Drosophila melanogaster* NatC complex

We further investigated the in vivo function of NatC using fruit fly *Drosophila melanogaster*, which provides an integrative model where genetics and behavioral tests can be combined. First, we performed a bidirectional BLAST search to identify the catalytic subunit of *Drosophila* NatC. We identified the protein sequence encoded by the gene *CG11412* as the most similar to human NAA30 and yeast Naa30 (Mak3), having an identity of 72.7% and 52.3%, respectively (Supplementary Fig. 11a). Supporting our analysis, *CG11412* was recently annotated by FlyBase as N(alpha)-acetyltransferase 30A (Naa30A). To confirm that Naa30A is a functional orthologue of human NAA30 and yeast Naa30, we expressed Myc-tagged Naa30A in *Drosophila* embryos, using the UAS/Gal4 system and the nanos-Gal4 driver^71–73^. By performing immunoprecipitation with an anti-Myc antibody coupled to mass spectrometry analysis, we identified the *Drosophila* orthologues of human and yeast NAA35/Mak10 (CG4065) and NAA38/Mak31 (CG31950) as interacting proteins of Naa30A (Supplementary Fig. 11b, c, Supplementary Data 6). In yeast, Golgi targeting of Arl3 requires NatC-mediated Nt-acetylation^30,74^. Interestingly, and further supporting functional conservation, ectopic expression of *Drosophila* Naa30A rescued the Arl3 localization defect in yeast *naa30*-deletion cells (Supplementary Fig. 11d). Altogether, our results suggest that NatC composition and substrate specificity are likely conserved between yeast, fly, and humans.

### *Drosophila Naa30A* deletion flies are viable

To study NatC function in flies, we generated a genomic *Naa30A* deletion using p-element-mediated imprecise excision. We thus created a *Naa30A* mutant animal where almost all the coding sequence of *Naa30A* gene was deleted (Supplementary Fig. 12a). This resulted in the deletion of the first 302 amino acids of Naa30A, with an additional frameshift mutation within the remaining coding sequence and a stop codon in the newly generated position 20. Surprisingly, deletion of *Naa30A* did not significantly impair adult viability. However, and suggesting underlying phenotypes, *Naa30A* deletion flies had a striking “held out” wing phenotype, characterized by the abnormal positioning of the wings, which were noticeably held further apart from the body axis in adult animals (Supplementary Fig. 12b). This phenotype is typical of animals with muscular defects^75^. We concluded that although NatC is likely to have hundreds of substrates, *Naa30A* is not essential for adult viability. This is possibly due to partial redundancy with other related paralogue proteins, like Naa30B and CG31730, or even with NatE and NatF, which have previously been suggested to share overlapping substrate specificity^76,77^.

### *Drosophila* NatC is required for normal longevity and to prevent age-dependent motility loss

**Fig. 6:**
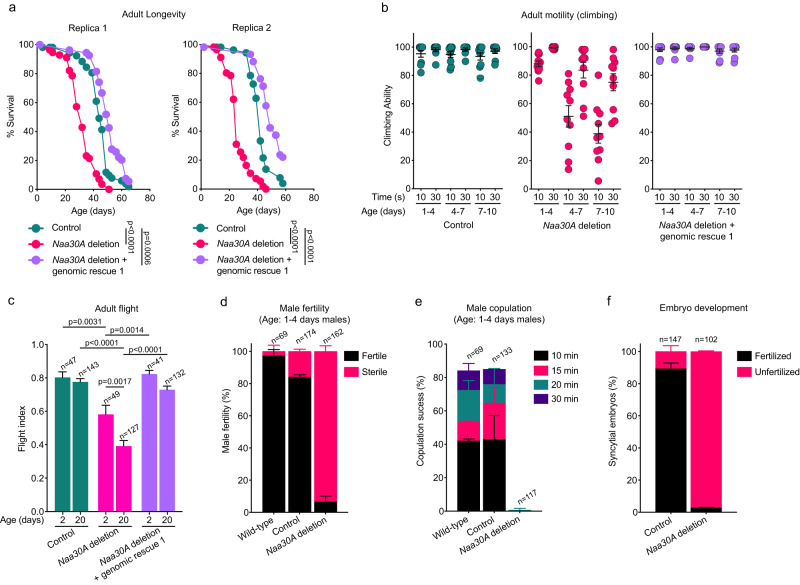
*Drosophila* Naa30A is required for normal longevity, fertility, and age-dependent motility. **a** Loss of Naa30A significantly reduces adult male longevity. Survival curves of control males, *Naa30A* deletion males, and *Naa30A* deletion males carrying the *Naa30A* genomic rescue 1 (two biological replicas). Statistical significance was assessed using the log-rank (Mantel–Cox) test, *n* > 50. **b** *Naa30A* deletion males show an accelerated age-dependent loss of motility. Males are from two independent biological replicas and age is indicated. Each data point represents the average climbing ability 10 males after 10 technical replicates, mean ± SEM is indicated. *Naa30A* deletion males exhibited a significant reduction in their climbing ability (1–4 days old males vs 4–7- or 7–10-days old males; *p* < 0.0001; one-way ANOVA with Šídák’s multiple comparisons test). **c** *Naa30A* deletion males show reduced flight ability. Flight ability of control males, *Naa30A* deletion males, and *Naa30A* deletion males carrying *Naa30A* genomic rescue 1, at the indicated age. Results are the mean ± SEM and were obtained with males collected from at least two independent crosses; n represents the total number of males tested. Statistical significance was assessed using the one-way ANOVA with Šídák’s multiple comparisons test. **d**–**f** *Naa30A* deletion males show reduced fertility and significant copulation defects. **d** Male fertility of wild-type males, control males and *Naa30A* deletion males (all 1–4 days old). Results are the mean ± SD and were obtained with males collected from 2 to 4 independent crosses; *n* represents the total number of males tested. **e** Copulation success was measured by the percentage of wild-type, control and *Naa30A* deletion males that were able to initiate copulation with 5–7 days old wild-type adult female virgins within the indicated time intervals. All males were 1–4 days old. Results are the mean ± SD of 2 independent crosses; *n* represents the total number of males tested. **f** Percentage of developing embryos (syncytial nuclear divisions or later stages of development) from wild-type (OR) females crossed with 1–4 days old control males or *Naa30A* deletion males. Results are the mean ± SD of two independent experiments; *n* represents the total number of embryos scored. All indicated ages are after pupae eclosion. Source data are provided as a Source Data file.

To further investigate the function of Naa30A, we performed longevity assays (see Methods). We found a significant longevity reduction of the *Naa30A* deletion males (Fig. 6a) (replica 1: median survival of control is 44 days after pupae eclosion vs 32 days of *Naa30A* deletion; replica 2: median survival of control is 42 days after pupae eclosion vs 25 days of *Naa30A* deletion). Importantly, the longevity defects of *Naa30A* deletion males could be fully rescued with a *Naa30A* genomic construct (genomic rescue 1) proving specificity (replica 1: median survival is 51 days after pupae eclosion; replica 2 median survival is 49 days) (Fig. 6a).

Beyond a significant reduction of longevity, we also noticed that *Naa30A* deletion males were apparently less active than control flies. To investigate this, we performed a climbing behavior assay. *Drosophila* negative geotaxis locomotor behavior is well established and has been used to study, for example, aging and distinct degenerative disorders^78–80^. While for control males climbing ability remain unchanged within the timeframe of this experiment, *Naa30A* deletion males showed a premature reduction of their climbing ability as they aged (Fig. 6b, Supplementary Fig. 12c, and Supplementary Movie 1). The reduced climbing ability of *Naa30A* deletion males could be fully rescued by *Naa30A* genomic rescue constructs inserted into two different sites of the genome (genomic rescue 1 and genomic rescue 2). Consistent with the “held out” adult wing phenotype of *Naa30A* deletion males, we also observed an age-dependent reduction of adult flight ability (Fig. 6c). The flight ability defects could also be fully rescued with a *Naa30A* genomic rescue (Fig. 6c).

A similar defect in adult longevity and loss of motility was also observed in homozygous *Naa30A* deletion females (Supplementary Fig. 13d, e). While the phenotypes may be more pronounced in females, the presence of these phenotypes clearly indicates that the role of NatC in longevity and mobility is not sex-specific. Altogether this suggests that NatC plays a crucial role in adult longevity and preventing age-dependent loss of motility in both males and females.

### *Drosophila* NatC is required for male copulation and fertility

Given that our results suggested underlying muscle defects induced by *Naa30A* deletion, we investigated other motility phenotypes beyond climbing and flight ability. We observed that *Naa30A* deletion males were essentially sterile, even when mated without male competition with receptive aged virgin females (Fig. 6d). To examine if defects in male copulation could be the underlying cause of *Naa30A* deletion male sterility, we measured copulation success and found that *Naa30A* deletion males did not copulate even in the presence of receptive aged virgin females (Fig. 6e). Consistent with this copulation defects, the eggs laid by females mated with *Naa30A* deletion males did not initiate development and appeared unfertilized (Fig. 6f).

The use of aged virgin females (5–7 days old), in the absence of male competition (e.g., single male crosses), mitigates the contribution of potential courtship defects for male sterility. Moreover, examination of testis morphology and sperm mobility in both control and *Naa30A* deletion males revealed no detectable abnormalities in spermatogenesis (Supplementary Fig. 13c, Supplementary Movies 2 and 3). It is worth noting that mouse mutants for Arg/N-degron pathway components have been reported to exhibit spermatogenesis defects^40,81,82^. However, in *Drosophila*, a closely related testis-specific paralog, Naa30B, has been annotated, which may explain the absence of spermatogenesis defects in this study. We therefore concluded that Naa30A is necessary for male fertility, potentially due to an essential function in the musculature of the male genitalia and terminalia, which are directly involved in the process of copulation.

### *Drosophila* NatC is seemingly not rate-limiting for mitochondrial function

NatC perturbation is associated with significant mitochondrial network defects in human cells (Fig. 5a and Supplementary Fig. 9)^16^. Since such defects could easily explain the motility and longevity phenotypes of *Drosophila Naa30A* deletion males, we decided to investigate if mitochondria were normal in the indirect flight muscles (IFM). We failed to detect obvious morphological defects of the mitochondrial network within muscles from *Naa30A* deletion males (Supplementary Fig. 13a). However, we detected a small reduction in the total levels of ATP in the absence of Naa30A (Supplementary Fig. 13b). Altogether, this suggests that although mitochondrial defects can potentially be a contributing factor to the observed *Naa30A* deletion phenotypes, they are not likely to be their main cause.

### *Drosophila* NatC is required to maintain muscle proteostasis

**Fig. 7:**
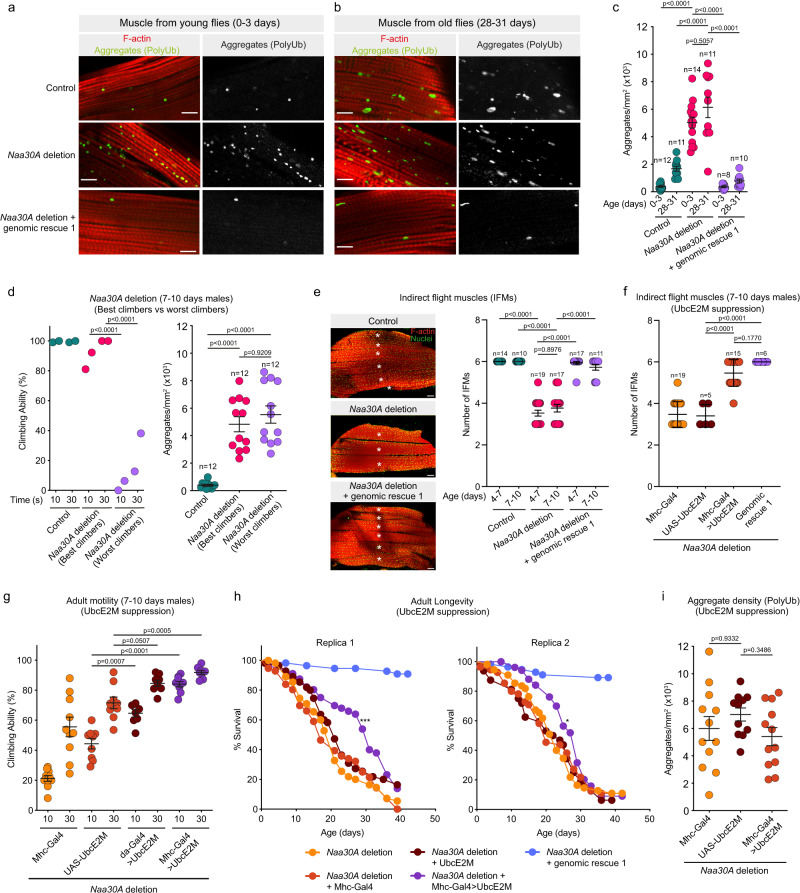
Overexpression of UbcE2M in the muscles suppresses the longevity and motility defects of *Drosophila Naa30A* deletion. **a**, **b** Accumulation of aggregate-like polyubiquitinated protein structures in muscles. Indirect flight muscles (IFM) from (**a**) young (0–3 days) and (**b**) old (28–31 days) males with the indicated genotypes. F-actin (red) and polyubiquitin (green). Scale bar, 10 μm. **c** Aggregate density (aggregate/mm^2^) as shown in **a** and **b**. Results are mean ± SEM. Each data point represents the aggregate density observed in a muscle fiber isolated from a male individual. **d** No correlation between the motility defects and aggregate density. Left panel: each data point represents the average climbing ability after 10 technical replicates of ~10 males (7–10 days). Two groups for each genotype were analyzed, mean ± SEM is indicated. Right panel: aggregate density (aggregate/mm^2^) in muscle fibers isolated from the males used in the climbing assay (left panel). Each data point represents the aggregate density observed in a muscle fiber isolated from a male individual. **e** Developmental defects of flight muscles. Left panel: IFMs stained for F-actin (red) and nuclei (green). Scale bar, 50 μm. *marks individual IFM. Right panel: Number of IFMs at the indicated ages. Data show the number of IFM per hemithorax. Mean ± SEM is indicated. **f** Developmental defects are rescued by UbcE2M overexpression in muscles. Number of IFMs in males in the indicated genotypes. Data show IFM per hemithorax and are dissected from 7 to 10 days old males. Mean ± SEM is indicated. **g** Overexpression of UbcE2M in the muscles suppresses the motility defects. Data show the average climbing ability after 10 technical replicates of a group of 10 males with 7–10 days, mean ± SEM is indicated. **h** Overexpression of UbcE2M in the muscles partially suppresses the adult longevity defects in *Naa30A* deletion. *p = 0.0455, ***p = 0.0005, log-rank test, *n* > 50). **i** UbcE2M overexpression does not suppress the accumulation of aggregate-like structures. Aggregate density (aggregate/mm^2^) observed in muscle fibers isolated from 0 to 3 days old *Naa30A* deletion males. Results are mean ± SEM and each data point represents a muscle fiber from a male individual. **c**–**g**, **i** Statistical significance was assessed using the one-way ANOVA with Šídák’s multiple comparisons and male specimens collected from two independent crosses. All indicated ages are after pupae eclosion. Source data are provided as a Source Data file.

Age-dependent loss of muscle function has been associated with proteostasis defects and the accumulation of protein aggregates in *Drosophila* and humans^83,84^. As expected, we observed an age-dependent increase in aggregate-like structures in control muscles using an antibody specific for mono- and polyubiquitinylated conjugates (Fig. 7a–c). Remarkably, analysis of young and old *Naa30A* deletion males showed a dramatic increase in the accumulation of polyubiquitinylated aggregates (Fig. 7a–c), clearly suggesting an important role of Naa30A in maintaining muscle proteostasis. However, since the total amount of protein aggregates were already high in young flies’ muscles, and their amount did not significantly increase with age (Fig. 7c), we reasoned that they were not likely to be directly related with the age-dependent motility defects of *Naa30A* deletion males. An alternative explanation would be that although young muscles have high levels of protein aggregates, older muscles are significantly more susceptible to aggregate accumulation.

To better understand this conundrum, we decided to compare the best and worse climbers within similarly aged *Naa30A* deletion males. For this purpose, we collected 200 *Naa30A* deletion males at 7–10 days of age after pupae eclosion. From these, we evaluated their climbing ability and selected the 9 males with best climbing ability (best climbers) and the 11 males with worst climbing ability (worst climbers). If protein aggregate accumulation is the cause of the observed age-dependent motility defects, then the best climbers should have less aggregates when compared to the worst climbers of a similar age and identical genetic background. We failed to detect any significant difference in the amount of protein aggregates within the muscles of the best and worst climbers of *Naa30A* deletion males (Fig. 7d). This suggests that although protein aggregate accumulation is suggestive of muscle proteostasis defects, these aggregates are however not causal for the motility defects of *Naa30A* deletion.

### *Drosophila* NatC protects from muscle developmental defects and age-dependent loss of muscle attachments

Given that the presence of protein aggregates is unlikely to be the underlying cause for the observed *Naa30A* deletion mutants’ motility defects, we decided to investigate if the age-dependent loss of motility resulted instead of developmental defects of the muscles. We analyzed the *Drosophila* IFM organization. Consistently, we found that *Naa30A* deletion males showed a significant reduction in the number of dorsal longitudinal IFM (Fig. 7e). This phenotype could be fully rescued by a *Naa30A* genomic rescue (Fig. 7e). The reduced number of adult dorsal longitudinal IFMs suggests defects in the splitting of the three sets of larval muscle fibers during metamorphosis, an event required to form the six dorsal longitudinal IFM fibers per hemisegment in adults^85^.

Additionally, we noticed an abnormal attachment of IFM muscles in *Naa30A* deletion males (Supplementary Fig. 13g), and this condition worsened with age (Supplementary Fig. 13h). This attachment phenotype frequently correlated with a reduced expression of Myocyte enhancer factor 2 (Mef2) (Supplementary Fig. 13g), an important myogenic transcription factor required for development of the muscles^86^^.^, and was fully rescued by a *Naa30A* genomic rescue construct (Supplementary Fig. 13h). Taken together, these findings fully support the hypothesis that Naa30A has a critical function for muscle development and avoidance of age-dependent loss of muscle attachments.

### Overexpression of UbcE2M suppresses the developmental and attachment defects of the muscles of *Naa30A* deletion mutants

Based on our observations that Nt-acetylation safeguard multiple human NatC substrates from degradation, (Fig. 3), we hypothesized that the developmental and attachment defects observed in the muscles of *Drosophila Naa30A* mutants were linked to NatC’s ability to shield a group of proteins from degradation. Among the most relevant NatC substrates found in this study are the NEDD8-conjugating enzymes UBE2M and UBE2F, which control neddylation of several Cullins^87^. Human UBE2M and UBE2F have partially overlapping roles^88^, while in *Drosophila* there is apparently only one homolog, UbcE2M, fulfilling their functions. Interestingly, the N-terminal sequence of *Drosophila* UbcE2M closely resembles the human UBE2M (Supplementary Fig. 7b), which suggests that it is very likely to be a NatC substrate. Moreover, there is an overall 75% identity and 84% similarity between both proteins.

To investigate if *Drosophila* UbcE2M is related to *Naa30A* deletion muscle developmental defects, we overexpressed UbcE2M using the UAS/Gal4 system^71^ and the Mhc-Gal4 driver^89^ for specific expression in the skeletal muscle. We observed that muscle-specific overexpression of UbcE2M was sufficient to fully rescue the number of dorsal longitudinal IFM after deletion of *Naa30A* (Fig. 7f and Supplementary Fig. 13f). Additionally, we also observed that in 7–10 days old flies, UbcE2M overexpression partially rescued the muscle attachment defects of *Naa30A* mutants (47.4% in *Naa30A* deletion males vs 13.3% in *Naa30A* deletion mutants with muscle specific overexpression of UbcE2M) (Supplementary Fig. 13i). Altogether, these results suggest that *Naa30A* and UbcE2M have critical functions for muscle proteostasis, correct development of the muscles, and avoidance of age-related loss of muscle attachment.

### Overexpression of UbcE2M in the muscles suppresses the longevity and motility defects of *Drosophila Naa30A* deletion

Since the developmental and age-dependent attachment defects of the muscles could be rescued by overexpression of *Drosophila* UbcE2M, we decided to investigate if the longevity and mobility phenotypes of the *Naa30A* deletion mutant could be similarly suppressed. Supporting the central role of UbcE2M in *Naa30A* mutant phenotypes, we observed that muscle-specific overexpression (using the Mhc-Gal4 driver) or ubiquitous overexpression (using the Da-Gal4 driver^90^) of UbcE2M was sufficient to rescue the climbing defects of *Naa30A* deletion males (Fig. 7g). Nevertheless, the accumulation of protein aggregates within the muscles was not reduced by UbcE2M overexpression (Fig. 7i), which fully supports our previous conclusion that these aggregates are not causal for the motility defects of *Naa30A* deletion males. Interestingly, muscle-specific overexpression of UbcE2M partially rescued the longevity defects of *Naa30A* deletion males (Fig. 7h), which highlights the importance of *Naa30A*-dependent muscle proteostasis and UbcE2M for longevity and age-dependent motility.

## Discussion

Modifications have major impacts on the N-terminal landscape of proteins, with some being mutually exclusive while others play in concert to influence key biological functions. Nt-acetylation catalyzed by the NATs is one of the most common protein modifications in eukaryotes, whose general role remains enigmatic. Mutations in the NATs can cause rare disorders, resulting in developmental delay, intellectual disability, and congenital heart defects^5–10^. The molecular framework and consequently the mechanistic basis for how the NATs manifest in cellular and organismal phenotypes remains, however, largely unknown. One of the major challenges is to identify which critical proteins NATs act on and mechanistically define why acetylation is important for the proper functioning of these proteins.

Here, we have performed unbiased genome-wide CRISPR KO screens to uncover genetic vulnerabilities related to Nt-acetylation. Our data revealed a strong interaction between the NatC complex and components of the Arg/N-degron pathway, highlighting a cellular system where Nt-acetylation protects against protein degradation. This study thus sheds new light on the intricate interplay between protein Nt-acetylation and the N-degron pathways, where the acetyl group can both promote or prevent selective protein degradation. For instance, Nt-acetylation may conditionally target proteins for degradation by the Ac/N-degron pathway, thereby playing a key role in protein quality control by removing improperly folded proteins and ensuring subunit stoichiometry^41,47,67^. In contrast, non-Nt-acetylated proteins, including those starting with methionine, may be degraded via the Arg/N-degron pathway in yeast^44^. A comprehensive mapping of yeast N-degrons indicated that the hydrophobic character is the predominant feature of N-degrons, not Nt-acetylation^91^. A direct and general impact on protein stability was also not observed when comprehensively assessing NatA and NatB deletion yeast strains, instead distinct cellular functions for the two major NATs were found towards gene regulation/genome integrity and protein folding, respectively^92^. A recent multiplexed proteomics investigation uncovered reduced ribosomal thermostability and increased turnover of ribosomal proteins in yeast lacking NatA, suggesting a positive impact of Nt-acetylation on protein stability possibly via protein-protein interactions and folding^93^. The complexity is further stressed by yeast NatA deletion impacting proteostasis via several different routes involving the Hsp90 chaperone and its client proteins^42^, increased proteasomal activity via Rpn4 regulation, as well as increased activity of several ubiquitin ligases^94^. In human cells, a fraction of non-Nt-acetylated NatA substrates can be targeted by IAP E3 ubiquitin ligases for proteasomal degradation^95^. Additionally, the E3 ubiquitin ligases ZER1-CRL2 and ZYG11B-CRL2 may also recognize a variety of non-Nt-acetylated NatA substrates^96^. Finally, NatA was shown to affect global protein turnover in *Arabidopsis*, via currently unknown N-recognin(s)^97^. In the current study, we not only show that unacetylated methionine-hydrophobic-starting N-termini are physically recognized by the Arg/N-degron pathway but find that the cellular phenotypes induced by NatC KO can be fully reversed by removal of specific N-recognins, revealing a principal role for Nt-acetylation in shielding proteins from degradation in human cells.

One of the strongest positive GIs identified in our *NAA30* and *NAA35* screens was the N-recognin UBR4^46^. Unlike other N-recognins, UBR4 appears to lack a classical ubiquitylation domain, suggesting that its modus operandi might deviate from that of canonical N-recognins and possibly requires other components of the N-degron pathway^45^. Indeed, UBR4 form a complex with the E3 ligase KCMF1^97–99^ and the E2 enzyme UBE2A (RAD6A)^60^, and both share a strong positive GI with *NAA30* and *NAA35*. The E3 enzyme KCMF1/UBR4 can cooperate with the E2 enzymes UBE2A (RAD6A) and UBE2D3 to regulate proteasomal and/or lysosomal mediated protein degradation and ER-associated degradation of membrane embedded substrates (ERAD-M), respectively^60,100–103^. In this study, we showed that non-Nt-acetylated NatC substrates are targeted primarily for proteasomal degradation by the UBE2A-KCMF1-UBR4 ubiquitin complex.

**Fig. 8:**
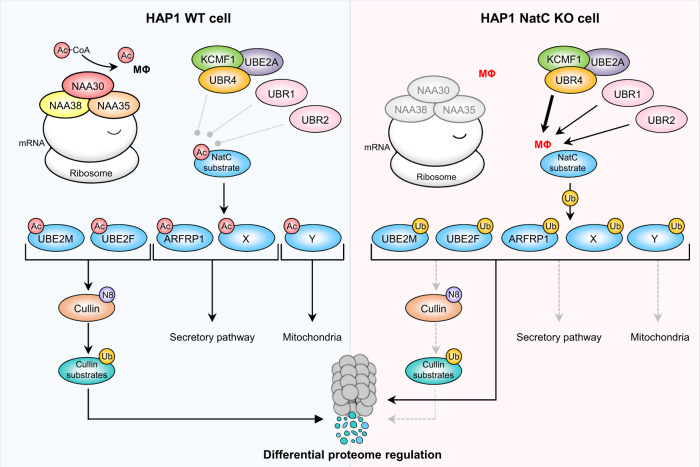
N-terminal acetylation by NatC shields proteins from degradation by preventing N-recognin UBR4-KCMF1 targeting. (**Left**) The NatC complex co-translationally acetylates proteins harboring a hydrophobic residue in the second position (MΦ-). Following Nt-acetylation, the NEDD8 E2 ligases Ac-UBE2M and Ac-UBE2F promote cullin neddylation (N8), resulting in ubiquitylation (Ub) and proteasomal degradation of targeted cullin substrates, Ac-ARFRP1 is targeted to the Golgi where it plays a role in the secretory pathway, while the hypothetical proteins Ac-X and Ac-Y are thought to affect the secretory pathway and mitochondria, respectively. (**Right**) Loss of NatC exposes unacetylated MΦ-starting N-termini which serves as N-degrons that can be recognized by a set of N-recognins leading to proteasomal and, in some cases, lysosomal degradation. Non-Nt-acetylated NatC substrates are primarily targeted by the Arg/N-recognin UBR4-KCMF1 and to some extent via UBR1 and UBR2. Targeted degradation of non-Nt-acetylated NatC substrates leads to decreased cullin neddylation, increased mitochondrial elongation and fragmentation, and is thought to affect intracellular trafficking.

The NatC-UBR sensitive phenotypes are most likely steered by several NatC substrates impacting vesicle trafficking, mitochondria, endosomes, and lysosomes (Fig. 8). Here, we studied selected confirmed and putative NatC substrates. Calpain small subunit 1 (CAPNS1, MF-) regulates the activity of calpain 1 and calpain 2 (CAPN1/2), two non-lysosomal cysteine proteases^104^. We found that both CAPNS1 and CAPN1 (MS-) were downregulated in NatC KO cells (Supplementary Data 4), indicating a role for NatC-mediated acetylation in subunit quality control^47^ where loss of CAPNS1 results in decreased CAPN1 protein levels. ARFRP1 (MY-) is a small GTPase regulating *trans*-Golgi trafficking and it is essential for glucose and lipid metabolism^105,106^. Both human ARFRP1 and the yeast orthologue Arl3 share negative GI with the NatC genes, supporting an evolutionarily conserved role for NatC in the ARL3-ARL1-SYS1 axis^29,30,74^. The two NEDD8-conjugating enzymes UBE2M (MI-) and UBE2F (ML-) have distinct substrate specificities. UBE2M interacts with the RING E3 ligase RBX1 while UBE2F pairs with RBX2 to regulate neddylation of CUL1-4 and CUL5, respectively^87^. Cullin neddylation is mediated through a dual E3 mechanism involving a co-E3 ligase called DCN1, which accelerates UBE2M/UBE2F binding^107–109^. Nt-acetylation acts as an avidity enhancer by facilitating the burial of their N-termini into a hydrophobic pocket of DCN1^26–28^. The key role of Ac-UBE2M and Ac-UBE2F is stressed by the fact that muscle developmental, longevity and motility defects of the *Drosophila Naa30A* deletion males could partially be complemented by overexpression of UbcE2M, the only *Drosophila* UBE2M/UBE2F homolog (Fig. 7). Considering that UBR knockdown increased UBE2M/UBE2F protein levels and restored neddylation of CUL5 in *NAA30*-KO cells (Fig. 5), the degradation of UBE2M/UBE2F may be a crucial molecular event following lack of its Nt-acetylation, not merely the impaired UBE2M-DCN1 complex. These two events are likely connected: non-Nt-acetylated UBE2M will be more dissociated from its partner DCN1 thereby exposing its free N-terminus to the UBRs which then target UBE2M for degradation. Still, the finding that UBR knockdown restores cullin neddylation in *NAA30*-KO cells (Fig. 5) suggests that UBE2M/UBE2F still form active neddylation complexes even in the absence of Nt-acetylation. Interestingly, UBE2M and UBE2F crosstalk with each other, where UBE2M can promote ubiquitination and degradation of UBE2F under both normal and stressed conditions^110^. Nt-acetylation is an irreversible modification, and its permanency suggests a role in proteostasis rather than signal transduction. In yeast, global Nt-acetylation levels are generally unaffected by prolonged starvation^111^, but fluctuations in cellular acetyl-CoA levels may affect Nt-acetylation and apoptotic fate in human cancer cells^112^. Further research is necessary to fully understand the effects NatC and Nt-acetylation have on protein neddylation under normal and stressed conditions.

Decline of human and *Drosophila* muscle function precedes most age-related changes and has been associated with defective proteostasis and the progressive accumulation of protein aggregates^84,113–115^. The contribution of proteostatic defects to the loss of muscular function remains, however, mechanistically poorly understood. Here, we showed that *Drosophila* NatC is required for longevity, motility, and normal development of muscles, as deletion of *Naa30A* is associated with decreased lifespan, lower climbing ability, male copulation defects, a reduced number of adult IFM fibers and age-dependent muscle attachment defects (Figs. 6 and 7 and Supplementary Fig. 13g–i).

We propose that Naa30A is required for the correct development of the muscles during larvae development. In the absence of Naa30A, muscle development and muscle attachment is abnormal. This results in muscle weakness and an exacerbation of the age-dependent loss of muscle function and reduced motility due to muscle detachment. Supporting our hypothesis that one of the critical organismal functions of *Drosophila* NatC is the regulation of protein stability, *Naa30A* deletion leads to a dramatic increase in the accumulation of protein aggregates in the muscles, whereas muscle-specific overexpression of UbcE2M suppressed the decreased longevity and motility defects induced by this deletion (Fig. 7). Although the precise molecular mechanisms by which Naa30A and UbcE2M regulate muscle development are still unknown, the observation that the levels of Mef2 transcription factor are downregulated in the detached muscle fibers suggest a tantalizing hypothesis where *Naa30A* mutant phenotypes are partially mediated by misregulation of this transcription factor, whose function is critical for muscle development^116,117^. Finally, and strongly suggesting that NatC’s effect on the cellular repertoire of the UBR4-KCMF1 complex is highly conserved among metazoans, *Drosophila* Ubr4/Poe also regulates muscle function and lifespan, with reduced adult flying and climbing activity^63,100^.

Loss of Ubr4 induces muscle hypertrophy, through decreased ubiquitination and degradation of a core set of target proteins^63,100^. Consistently, muscle-specific knockdown of Ubr4 increased the number of poly-ubiquitinated protein aggregates, as Ubr4 directly associates with and increases the proteolytic activity of the proteasome. It was therefore suggested that these muscle defects most likely arose from reduced proteasome-mediated proteolysis and not via the autophagy-lysosome system^100^. However, the relationship between protein aggregate accumulation and loss of muscular function remains unclear, as most available evidence is correlative without a clear link to causality. The fact that we failed to detect a clear correlation between the amount of protein aggregates and the degree of climbing fitness among *Naa30A* deletion flies with similar age suggests no phenotypic causality. Furthermore, and significantly, overexpression of UbcE2M in the muscles was sufficient to rescue muscle development and adult motility, without reducing protein aggregate accumulation in the muscles. This clearly demonstrates that although NatC regulation of the UBR4-KCMF1 substrates is critical to muscular function, the functional relevance of protein aggregate accumulation, at least in this context, is less clear. Altogether, these results suggest that *Drosophila Naa30A* deletion phenotypes can be explained by increased degradation of a subset of UBR4 target proteins due to a lack of NatC-mediated acetylation.

In conclusion, using an unbiased and global genetic screen, we uncovered a general function for NatC-mediated Nt-acetylation in protecting proteins from degradation in human cells. Our molecular investigations defined the ubiquitin ligases UBR4-KCMF1 and partially UBR1/UBR2 as responsible for degrading a major class of human proteins when lacking Nt-acetylation. The role of NatC-mediated protection of enzymes involved in cullin neddylation is evident both in human cells and in *Drosophila*. The impact of these pathways on longevity and motility in aged individuals underscores the vital role of protein Nt-acetylation.

## Methods

### Cell lines and culturing conditions

The near haploid human HAP1 WT cell line (clone C631; sex: male; RRID:CVCL_Y019) and the HAP1 gene-KO cell lines *NAA30*-KO (HZGHC006637c010) and *NAA35*-KO (HZGHC006636c011) were obtained from Horizon Genomics GmbH (Vienna, Austria). HAP1 *NAA38*-KO (KO2 in original ref) and an unmodified HAP1 control cell line that underwent CRISPR treatment (herein referred to as WT2) were a generous gift from Dr. Scott Dixon, Stanford University^31^. Gene disruption in the HAP1 KO cell lines were confirmed by Sanger sequencing of PCR products of the edited genomic region. HAP1 cells were maintained in IMDM (Gibco) supplemented with 10% FBS and 1% penicillin-streptomycin (both from Sigma-Aldrich). The human cervix carcinoma cell line HeLa (clone CCL-2; sex: female; RRID:CVCL 0030) and human breast cancer cell line MDA-MB-231 (sex: female; RRID:CVCL_0062) were obtained from ATCC. Both cell lines were maintained in high glucose DMEM supplemented with 10% FBS, 4 mM L-glutamine and 1% penicillin-streptomycin (all from Sigma-Aldrich). Cells were detached using Trypsin (Gibco) and maintained at 37 °C in a humidified atmosphere with 5% CO_2_. Cells were routinely tested for mycoplasma using the MycoAlert Detection Kit (Lonza) or by NucBlue DAPI staining (Invitrogen). For CRISPR screening HAP1 cells with lowest possible passage number were used. For all other experiments, HAP1 cell lines were passaged and sorted until diploid status was confirmed by an Accuri C6 flow cytometer (BD) using propidium iodide staining before they were used in experiments^118^. The HeLa cell line has not been authenticated and was only used for transient transfection of NAA30-V5 for immunoprecipitation and acetylation assays and UBR4-V5 for peptide pulldown assays.

### CRISPR/Cas9-mediated *NAA30* gene disruption in human MDA-MB-231 cell line

CRISPR/Cas9-mediated *NAA30* disruption was performed in monoclonal MDA-MB-231 cells using pSpCas9(BB)−2A-GFP (Addgene, #48138)^119^. GuideRNA (gRNA) targeting exon 2 of human *NAA30* was designed using the CHOPCHOP v3 web tool (https://chopchop.cbu.uib.no/)^120^, cloned into pSpCas9(BB)−2A-GFP using the BbsI restriction site and verified by Sanger sequencing. MDA-MB-231 cells were transfected with a plasmid targeting *NAA30* or a non-targeting control using Lipofectamine 2000 (Invitrogen) according to the manufacturer’s instructions (see Supplementary Data 7 for sgRNA sequences). Transfected cells were single cell sorted based on the transiently expressed GFP marker using a Sony SH800 cell sorter (Sony Biotechnology) into 96-well plates containing high glucose DMEM with 10% FBS. Following several weeks of expansion, a *NAA30*-KO clone was verified by PCR sequencing from genomic DNA followed by immunoblot analysis using anti-NAA30 (Sigma Aldrich, # HPA057824). A non-targeting clone served as a post-transfection control cell line (here referred to as MDA-MB-231 WT).

### Genome-wide CRISPR screens

The human Toronto knockout v3 (TKOv3) genome-scale CRISPR library (Addgene #90294) was used to perform pooled CRISPR knockout screens^121^. The library is composed of 70,948 guides targeting 18,053 protein-coding genes (4 guides/gene). The screens were performed essentially as described in^53^. Briefly, 3 million HAP1 cells were seeded in 15-cm plates with 20 mL of IMDM medium containing 8 μg/mL polybrene (Sigma-Aldrich). A minimum of 90 million target cells were transduced with TKOv3 lentivirus at an MOI of 0.3, such that each sgRNA was represented in about 200–300 cells after selection (>200-fold coverage of the library). After 24 h, the TKOv3 transduced cells were selected for viral integration with 25 mL IMDM medium containing 1 μg/mL puromycin (Sigma-Aldrich) for 48 h. Then, the cells were harvested, pooled, and split into three replicates of at least 15 million cells maintaining a 200-fold coverage of the library. 20–30 million cells were collected for genomic DNA extraction after puromycin selection (T0 reference), and at every passage thereafter. Representation of the TKOv3 sgRNA library was maintained at >200-fold at each step of the screening workflow for each replicate, and cell passaging continued for up to 15 population doublings. The HAP1 *NAA35*-KO screens were performed three separate times with three replicates each, whereas the *NAA30* and *NAA35* screens were performed once with three replicates each.

Genomic DNA was extracted from the cell pellets using the Wizard Genomics DNA Purification Kit (Promega) and the concentration was determined using the Qubit dsDNA Broad Range Assay kit (Invitrogen). Sequencing libraries were prepared from 50 μg gDNA (200-fold library representation for haploid genome) via a 2-step nested PCR strategy with NEBNext Ultra II Q5 Polymerase (NEB) using primers that include Illumina TruSeq adapters with i5 and i7 indices. Barcoded libraries were gel-purified using PureLink Quick Gel Extraction kit (ThermoFisher), sequenced on an Illumina HiSeq2500 (RRID:SCR_016383) using single-read sequencing, and where completed with standard sequencing primers for dual-indexing with HiSeq SBS Kit v4 reagents as described in^53^. All primer sequences can be found in Supplementary Data 7. The CRISPR screen sequencing data have been deposited in NCBI’s Gene Expression Omnibus^122^ and are accessible through GEO Series accession number GSE221447.

### Genetic interaction analysis

Screens were analyzed using methods described in^53^. Briefly, demultiplexed FASTQ files were first trimmed by locating constant sequence anchors and extracting 20-bp guide sequences preceding the anchor sequence. Pre-processed single-end reads were aligned to TKOv3 reference library sequences using Bowtie (v0.12.8) allowing up to two mismatches and one exact alignment (specific parameters: -v2 -m1 -p4 --sam-nohead). Successfully aligned reads were counted and combined into a matrix with guide annotations. Differential effects between HAP1 *NAA30*-KO, *NAA35*-KO, *NAA38*-KO and HAP1 WT screens were estimated using the approach described in Aregger et al. in the methods section entitled ‘Scoring of quantitative GIs with the qGI score'^53^. Briefly, read counts for all samples were normalized to ‘counts per million’ (cpm) by dividing each read count by the sum of all read counts in the sample then multiplying by one million and adding a pseudo count of 0.5. Guides with fewer than 40 or greater than 10,000 absolute read counts at T0 were removed. Log_2_ fold-changes (LFCs) between normalized guide abundance at each sequenced timepoint and the starting timepoint (T0) of each screen were computed. LFC values for each guide were averaged across the technical replicates, and guide-level residual LFCs between each screen and the corresponding control screen were computed. To correct for potential non-linear relationships between the treatment and control data, a second-degree LOESS curve with a span equal to 40% of the residual LFCs was fit before subtracting its predicted values from the residual LFCs. To penalize potential false positive large residual LFCs due to low T0 readcounts, residual LFCs were adjusted for their dependency on T0 readcount levels. Gene-level *p*-values reflecting the difference gene knockout and WT screens were calculated from LOESS-normalized residual LFCs with moderated t-testing as implemented in the limma R package^123^. Multiple hypothesis testing was corrected for using the Benjamini–Hochberg method. Gene-level differential effects, the genetic interaction (GI) scores, were computed by taking the mean residual LFC for all genes with two or more remaining guides post-filtering based on T0 read counts. The Pearson correlation coefficients of the qGI scores measured in two replicated screens were adjusted to the similarity of a *NAA35*-KO screen to a panel of HAP1-KO screens. The resulting Within vs Between replicate Correlation (WBC) score provides a confidence of reproducibility that can be interpreted as a z-score^54^. The qGI scores can be found in Supplementary Data 1.

### HAP1 cell harvesting for proteomics analysis

HAP1 WT, *NAA30*-KO, *NAA35*-KO, and *NAA38*-KO cells were grown in 10-cm dishes to 70–80% confluency. Cells were washed twice in ice-cold DPBS buffer (Gibco), detached by scraping in ice-cold DBPS with 1 × cOmplete protease inhibitor (Roche), and collected by centrifugation 16,000 × *g*, 15 s, 4 °C (twice). Cell pellets were flash frozen in liquid nitrogen and stored at −80 °C until further processing. For each cell line, four samples per proteome study were used. The N-terminal acetylation status was determined by positional proteomics using strong cation exchange (SCX) enrichment, while protein abundance was determined by label-free quantitative (LFQ) shotgun proteomics. Detailed information is given below.

### N-terminal acetylation proteomics

As protein N-terminal acetylation (either in vivo or in vitro) neutralizes the N-terminal positive charge, the charge of N-terminal peptides is altered compared to that of internal peptides. This altered biophysical property can be used to enrich N-terminal peptides by using low pH strong cation exchange (SCX) chromatography. SCX enrichment of N-terminal peptides from HAP1 control and NatC KO cells was performed essentially as previously described^59^. Cell pellets were thawed on ice, resuspended in 1.5 mL of ice-cold CHAPS lysis buffer (50 mM sodium phosphate pH 7.5, 100 mM NaCl, 0.8% (wt/vol) CHAPS in water, and 1 × cOmplete EDTA-free protease inhibitor cocktail (Roche)) and incubated on ice for 30 min. Samples were cleared by centrifugation at 16.000 × *g* for 15 min at 4 °C. Protein concentrations were determined using Bradford reagent (Bio-Rad) and 1 mg of proteins was collected. To this, guanidinium chloride (GuHCl) was added to a final concentration of 2 M. Subsequently, proteins were reduced and alkylated for 15 min at 37 °C in the dark and at a pH of 7.9 with iodoacetamide (30 mM f.c.) and TCEP-HCl (15 mM f.c.). Samples were subsequently desalted on Illustra NAP-10 columns (GE Healthcare, Cat # 17085402) and eluted in 50 mM sodium phosphate pH 8 containing 1.33 M GuHCl, after which the volume was reduced from 1.5 mL to 1 mL by vacuum drying (to reach a final concentration of 2 M GuHCl). To enable the assignment of in vivo N-terminal acetylation events (Ac), all primary protein amines were blocked at the protein level making use of a N-hydroxysuccinimide ester of acetic acid encoded with stable heavy isotopes (i.e. an NHS ester of ^13^C_1_D_3_-acetate^124^). In this way, it is possible to distinguish between in vivo (Ac) and in vitro (AcDC) acetylated N-termini and to calculate the degree of in vivo Nt-acetylation as described below. Samples were acetylated with 10 mM (final concentration) of AcDC for 1 h at 30 °C. The NHS ester was added once more, and incubation proceeded for an additional hour at 30 °C. The excess of acetylation reagent was quenched with 40 mM glycine (2-fold molar excess) and incubated for 10 min at room temperature (RT) followed by the addition of 100 mM hydroxylamine and an incubation for an additional 10 min at RT. Samples were then desalted on a Illustra NAP-10 column (GE Healthcare) and eluted in 10 mM ammonium bicarbonate buffer (pH 7.6). Before trypsin digestion, samples were boiled for 5 min and cooled for 10 min on ice. Trypsin (Promega, Cat # V5111) was added in a trypsin/protein ration of 1/50 (w/w) and incubated ON at 37 °C. Samples were then vacuum-dried.

Dried peptides were re-dissolved in 212 µL of pyro-glu buffer (16 mM NaCl, 0.5 mM EDTA, 3 mM cysteamine and 50 μM aprotinin (Roche)). Purified pGAPase was activated by adding 1 μL of 800 mM NaCl, 1 μL of 50 mM EDTA (pH 8.0) and 11 μL of 50 mM cysteamine, and was incubated for 10 min at 37 °C. Activated pGAPase and Q-cyclase (both parts of the Tagzyme kit, Qiagen, Cat # 34342) were added to the peptides and incubated for 1 h at 37 °C.

Samples were diluted to 1 mL with SCX buffer A and pH was adjusted to 2.98–3.02. Peptide concentrations were measured on Lunatic microfluidic device (Unchained Labs) and 700 μg of peptide material was loaded onto the dual mode SCX cartridge according to the following protocol: a) wet with acetonitrile (1 mL); b) wash with Milli-Q water (1 mL); c) equilibrate with SCX buffer A (2 mL); d) load sample (1 mL); e) wash with SCX buffer A (1 mL); f) elute with SCX buffer B (5 mL) and 1 mL of SCX buffer B containing 5 mM NaCl, and this 6 mL was collected; g) adjust the pH of the elution to about 6.0. Peptides were then dried and re-dissolved in 1 mL in loading buffer (0.1% TFA and 2% ACN). Methionines were oxidized by 0.06% H_2_O_2_ (final concentration) for 30 min at 30 °C, followed by SampliQ SPE C18 desalting and clean-up (Agilent Technologies, Cat # 5982-1135). Peptides were eluted in elution buffer (60% acetonitrile (ACN), 0.1% TFA), vacuum-dried and stored at −20 °C. SCX buffer A: mix buffer A1 (60 mg NaH_2_PO_4_ + 50 ml H_2_O) with buffer A2 (60 mg H_3_PO_4_ + 50 mL H_2_O) to a pH of 3.00. SCX buffer B: mix buffer B1 (57.6 mg NaH_2_PO_4_ + 15 mL H_2_O + 35 mL acetonitrile) with buffer B2 (57.6 mg H_3_PO_4_ + 15 mL H_2_O + 35 mL acetonitrile) to a pH of 3.00.

Purified peptides were re-dissolved in 22 μL loading solvent A (0.1% TFA in water/ACN (98:2, v/v)) and the peptide concentration was measured on the Lunatic microfluidic device. 2 μg of peptides were injected for LC-MS/MS analysis on an Ultimate 3000 RSLCnano system in-line connected to an Orbitrap Fusion Lumos mass spectrometer (Thermo). Trapping was performed at 10 μL/min for 4 min in loading solvent A on a 20 mm trapping column (made in-house, 100 μm internal diameter (I.D.), 5 μm beads, ReproSil-Pur Basic-C18-HD (Dr. Maisch, Germany). The peptides were separated on a 200 cm µPAC column (C18-endcapped functionality, 300 μm wide channels, 5 μm porous-shell pillars, inter pillar distance of 2.5 μm and a depth of 20 μm; PharmaFluidics, Belgium). The column temperature was kept constant at 50 °C. Peptides were eluted by a linear gradient reaching 55% MS solvent B (0.1% formic acid (FA) in water/acetonitrile (2:8, v/v)) after 115 min, 99% MS solvent B at 120 min, followed by a 10-min wash at 99% MS solvent B and re-equilibration with MS solvent A (0.1% FA in water). The first 15 min the flow rate was set to 750 nL/min after which it was kept constant at 300 nL/min. The mass spectrometer was operated in data-dependent top speed mode with a cycle time of 3 s, automatically switching between MS and MS/MS acquisition. Full-scan MS spectra (300–1500 m/z) were acquired at a resolution of 120,000 in the Orbitrap analyzer after accumulation to an AGC value of 200,000 with a maximum injection time of 250 ms. The most intense ions above a threshold value of 5000 and a charge state ranging from 2 to 7, subjected to a dynamic exclusion of 60 s, were isolated in the quadrupole for fragmentation in the ion routing multipole at a normalized collision energy of 34% after accumulation of precursor ions at a target value of 12,000 with a maximum of 40 ms with an isolation width of 1.2 Th. The fragments were analyzed in the Ion Trap Analyzer at normal scan rate.

Mascot generic files were created using the Mascot Distiller software (version 2.7.1.0, Matrix Science). Peak lists were searched with the Mascot search engine using the Mascot Daemon interface (version 2.6.2, Matrix Science) against the SwissProt database restricted to human proteins (*Homo sapiens* database release version of January 2020). Spectra were searched twice, one search to quantify the N-terminal acetylated peptides and another search to identify all the peptides including the ones that are not N-terminally acetylated. Both peptide searches were set with semi-ArgC/P enzyme (/P indicates arginine can also be cleaved when followed by a proline), allowing 1 missed cleavage; heavy acetylation (^13^C_1_D_3_) of lysine residues, carbamidomethylation of cysteine residues and oxidation of methionine were set as fixed modifications. Mass tolerance of the precursor ions was set to 10 ppm (with Mascot’s C13 option set to 1) and of fragment ions to 0.5 Da. The instrument was set to ESI-TRAP. In the first search, to allow identification and quantification of N-terminal acetylated peptides, a quantitation method with two different components was made, defining light and heavy acetylation of peptide N-termini as respectively light and heavy exclusive modification groups. Only peptides that were ranked first and scored above the threshold score set at 99% confidence were withheld. In a second search, peptides without N-terminal acetylation were identified using a similar search parameter set, including the cyclization of N-terminal glutamine residues to pyroglutamate (pyro-Glu) residues as a variable modification. Identified peptides were quantified using Mascot Distiller Toolbox (version 2.7.1.0, Matrix Science) in the precursor mode. For processing of all MS data, the ms_lims software platform was used^125^. Peptides shorter than 8 amino acid residues were removed for downstream analysis. To further check the N-terminal data, all quantified acetylated peptides were extracted from ms_lims. After extraction, peptides were filtered on confident spectrum (“TRUE”), tryptic cleavage and start position (one or two) to retrieve the true N-terminal peptides. The degree of in vivo acetylation was calculated separately using the light/heavy (Ac/AcDC) ratio reported by the Mascot Distiller Toolbox and by the equation Ac% = ratio (Ac/AcDC)/(ratio (Ac/AcDC) + 1). In those cases where N-terminal peptides were identified and quantified multiple times, the median and standard deviation of the acetylation degrees of the individual peptides was calculated. The mass spectrometry proteomics data have been deposited to the ProteomeXchange Consortium via the PRIDE partner repository^126^ with the dataset identifier PXD034992. Processed data are presented in Supplementary Data 3.

### Label-free quantitative shotgun proteomics

Cell pellets were dissolved in 0.5 mL urea lysis buffer (8 M urea pH 8.0, 20 mM HEPES). The cells were then sonicated 3 × 15 s (with 1 min incubations on ice) and centrifuged at 16,000 × *g* for 15 min at RT. The protein concentrations of the samples were determined by the Bradford assay (Bio-Rad). Proteome samples were prepared in quadruplicate for each condition. From each replicate, equal protein amounts (1 mg) were used for further analysis. Proteins were reduced with 5 mM DTT f.c. for 30 min at 55 °C and alkylated with 10 mM f.c. iodoacetamide at RT in the dark for 15 min. The samples were diluted with 20 mM HEPES at pH 8.0 to achieve a urea concentration of 4 M and then the proteins were digested with 1/100 (w/w) endoLysC (Lysyl Endopeptidase, Wako, Cat # 121-05063) for 1.5 h at 37 °C. All samples were further diluted with 20 mM HEPES at pH 8.0 to a final urea concentration of 2 M, and the proteins were digested with 1/100 (w/w) trypsin (Promega, Cat # V5111) overnight at 37 °C. Digested samples were acidified with TFA (to a final concentration of 1% TFA), which allows precipitation of undigested proteins. Samples were incubated on ice for 15 min, centrifuged at 16,000 × *g* for 15 min at RT, and the supernatant was transferred to a new tube. The peptides (half of the sample was used, the other half was stored) were then purified on a SampliQ SPE C18 cartridge (Agilent Technologies, Cat # 5982-1111.). The eluted peptides were dried and stored at −20 °C before MS analysis.

MS/MS analysis was performed similarly as described for the enrichment of N-terminal peptides (acetylation proteomics) with some minor changes outlined below. Peptides were separated on an Ultimate 3000 RSLCnano system in-line connected to an Orbitrap Fusion Lumos mass spectrometer (Thermo Scientific) with a linear gradient reaching 55% MS solvent B after 145 min and 99% MS solvent B at 150 min, followed by a 10-min wash at 99% MS solvent B and re-equilibration with MS solvent A. All other settings were kept as described above.

The generated MS/MS spectra were processed with MaxQuant (version 2.0.1.0)^127^ using the Andromeda search engine with default search settings, including a false discovery rate set at 1% on both the peptide and protein level. Spectra were searched against the sequences of the human proteins in the Swiss-Prot database (release May 2021). The enzyme specificity was set at trypsin/P, allowing for two missed cleavages. Variable modifications were set to oxidation of methionine residues and N-terminal protein acetylation. Carbamidomethylation of cysteine residues was put as fixed modification. In the settings of advanced identifications, matching between runs was enabled (with standard settings). All other settings were kept as standard. Proteins were quantified by the MaxLFQ algorithm integrated in the MaxQuant software. A minimum of two ratio counts and both unique and razor peptides were considered for protein quantification. The iBAQ function was also used. Further data analysis was performed with the Perseus software (version 1.6.15.0)^128^ after loading the protein groups file from MaxQuant. Proteins only identified by site, reverse database hits and potential contaminants were removed, and replicate samples were grouped. Proteins with less than three valid values in at least one group were removed, and missing values were imputed from a normal distribution around the detection limit. Then, a multiple sample t-test was performed (one-way ANOVA, FDR = 0.01 and S0 = 0) after which the z-scores of the significant proteins of each sample were calculated and further analyzed by hierarchical clustering (Euclidean with standard Perseus settings). Pairwise comparisons were also performed by a t-test (FDR = 0.01 and S0 = 0.1) between the different KO cell lines and control samples. The mass spectrometry proteomics data have been deposited to the ProteomeXchange Consortium via the PRIDE partner repository^126^ with the dataset identifier PXD034104. Processed data are presented in Supplementary Data 4.

### Plasmids

The plasmid pCMV6-UBE2M-Myc-DDK (DDK is the same as FLAG) was obtained from OriGene Technologies (Rockville, Maryland, USA, # RC208946). pcDNA3.1-NAA30-V5 was previously described and was constructed using cDNA from a human cell line^24^. For both plasmids, point mutations were introduced using the Q5 Site-Directed Mutagenesis Kit (New England Biolabs) according to the manufacturer’s instructions. Mutations were confirmed by Sanger sequencing. pSpCas9(BB)−2A-GFP (PX458) was a gift from Dr. Feng Zhang (Addgene plasmid #48138; RRID:Addgene_48138). *NAA30* sgRNA and a non-targeting control was cloned into PX458 as described^119^. The bicistronic pUC-UBE2M-V5-P2A-GST-GFP plasmid was custom-made by VectorBuilder (Chicago, Illinois, USA) and expresses the indicated UBE2M P2A construct from a *CMV* promoter (see Source Data File for full plasmid sequence). pcDNA6.2-hUBR4-V5-Lumio plasmid was a kind gift from Dr. Yong Tae Kwon, Seoul National University College of Medicine, and contains a 15.9-kb human *UBR4* open reading frame^46^. The plasmids pCDH-EF1-UBR4N-FLAG-T2A-copGFP and pCDH-EF1-UBR4C-FLAG-T2A-copGFP were a kind gift from Dr. Fabio Demontis and Dr. Liam C. Hunt, St. Jude Children’s Research Hospital^63^. These plasmid expresses FLAG-tagged truncated variants of mouse *UBR4*, where UBR4-N corresponds to a N-terminal region which includes the UBR box (p.1222-1996) while UBR4-C corresponds to the C-terminal UBR4 domain (p.4364-5180). *Drosophila Naa30A* was amplified with the introduction of a N-terminal HA-tag from the cDNA clone LD45352 (DGRC Stock 3896; RRID:DGRC_3896) and then subcloned into pBEVY-U^129^ downstream of the *ADH1* promoter using the *XmaI* and *EcoRI* sites, yielding plasmid pBEVY-U-HA-DmNAA30. pBEVY-U-HA-hNAA30 was previously described and expresses human NAA30 from the *ADH1* promoter^74^. All primers and plasmids can be found in Supplementary Data 7 and Supplementary Data 8, respectively.

### Immunoblot analysis

Cells were washed with cold PBS (Gibco), scraped on ice, and collected at 16,000 × *g* for 15 s at 4 °C. Cell pellets were lysed in IPH lysis buffer (50 mM Tris-HCl pH 8.0, 150 mM NaCl, 5 mM EDTA, 0.5% NP-40) supplemented with 1 × cOmplete EDTA-free protease inhibitor cocktail (Roche) for 30 min on ice. Cell lysates were cleared by centrifugation at 16,000 × *g* for 5 min at 4 °C and protein concentration was determined using the Pierce BCA protein assay kit (Thermo Scientific). Cell lysates were mixed with Laemmli sample buffer (Alfa Aesar) and denatured at 95 °C for 5 min. Next, 20–30 μg of total protein was resolved on 8–16% TGX stain-free gels and transferred onto nitrocellulose membrane using the Trans-Blot Turbo RTA transfer kit and transfer system set to 7 min transfer protocol (all from Bio-Rad). Stain-free gels and blots were imaged using the Gel Doc EZ imaging system (Bio-Rad) when applicable. For the UBR4 blots, proteins were typically resolved on 7.5% TGX stain-free gels and transferred onto nitrocellulose membrane (GE Healthcare, Amersham Protran) using wet-transfer at 100 V for 60 min. All blots were blocked for 1 h at RT in 5% non-fat dry-milk (AppliChem) resuspended in 1 × TBS-T (20 mM Tris pH 7.5, 150 mM NaCl, 0.05% (v/v) Tween-20). Subsequently, the blots were incubated with primary antibody diluted in 1% blocking buffer overnight at 4 °C followed by washing and 2 h incubation with HRP-conjugated secondary antibody diluted in 3% blocking buffer. Protein bands were visualized using SuperSignal West Pico chemiluminescent substrate (Thermo Scientific) and the chemiluminescent signals were captured with a ChemiDoc XRS+ imaging system coupled with Image Lab Software version 6.0.1 (both from Bio-Rad). Densitrometry analysis was performed using Image Lab 6.0.1. All immunoblots were performed at least three independent times with biologically/experimentally independent samples. Uncropped immunoblots are provided in Source Data File.

The following antibodies were used: anti-ARFRP1 (Sigma-Aldrich, HPA04702, 1:1000), anti-BCL2 (Proteintech, 12789-1-AP, 1:500), anti-CAPNS1 (Thermo Scientific, PA5-82266, 1:1000), anti-CUL1 (Cell Signaling, 4995, 1:1000), anti-CUL2 (Abcam, ab166917, 1:1000), anti-CUL3 (Cell Signaling, 2759, 1:1000), anti-CUL4A (Cell Signaling, 2699, 1:1000), anti-CUL4B (Proteintech, 12916-1-AP, 1:1000), anti-CUL5 (Abcam, ab264284, 1:1000), anti-DIS3 (Thermo Scientific, PA5-78427, 1:1000), anti-EEA1 (Santa Cruz, sc-33585, 1:1000), anti-FLAG (Sigma, clone M2, F3165, 1:3000), anti-GAPDH (Santa Cruz, sc-47724, 1:10000), anti-GFP (Roche, clones 7.1 and 13, 11814460001, 1:5000), anti-HK1 (Thermo Scientific, MA5-14789, 1:2000), anti-IST1 (GeneTex, GTX101972, 1:1000), anti-KCMF1 (Sigma-Aldrich, HPA030383, 1:1000), anti-LC3B (Thermo Scientific, PA1-46286, 1:1000), anti-NAA30 (Sigma-Aldrich, HPA057824, 1:1000), anti-p62/SQSTM1 (Santa Cruz, sc-28359, 1:300-1:1000), anti-RBX1 (Abcam, ab221548, 1:1000), anti-RBX2/RNF7 (Abcam, ab181986, 1:1000), anti-RGS10 (Abcam, ab154172, 1:1000), anti-RSPRY1 (Thermo Scientific, PA5-32048, 1:1000), anti-SLC10A7 (Sigma-Aldrich, SAB2102163, 1:1000), anti-UBE2A/UBE2B (Abcam, ab31917, 1:2000), anti-UBE2F (Abcam, ab185234, 1:1000), anti-UBE2M (Abcam, ab109507, 1:1000), anti-UBR1 (Bethyl Laboratories, A302-988A-M, 1:1000), anti-UBR2 (Abcam, ab217069, 1:2000), anti-UBR4 (Abcam, ab86738, 1:1000), anti-UBR5 (Cell Signaling, 65344, 1:1000), anti-V5 (Invitrogen, R960CUS, 1:5000-20,000), anti-vinculin (Abcam, ab129009, 1:10000), HRP-linked-sheep-anti-Mouse (Cytiva, NA931, 1:3000-1:20000), and HRP-linked-donkey-anti-Rabbit (Cytiva, NA934, 1:1000-1:10000).

### N-terminal variants of UBE2M-FLAG

To investigate the stability of N-terminal variants of UBE2M, HAP1 *NAA30*-KO cells were transiently transfected with a set of Met-X-UBE2M-FLAG constructs (pCMV6-UBE2M-Myc-DDK) where the second residue was glycine, alanine, (NatA-type substrates), aspartic acid, glutamic acid, (NatB-type substrates), isoleucine (native), leucine, tyrosine, phenylalanine (NatC/E/F-type substrates), or proline (stabilizing control). HAP1 *NAA30*-KO cells were seeded at a density of 2.5 × 10^6^ cells per 10-cm dish and incubated overnight. The medium was replaced, and the cells were transfected for 24 h with a mixture of 6 μg plasmid DNA, 24 μL X-tremeGENE 9 DNA transfection reagent (Roche) and 500 μL Opti-MEM media (Gibco), according to the manufacturer’s instructions. Protein levels were determined by immunoblotting using anti-FLAG clone M2 (Sigma, F3165, 1:3000). The experiment was performed three independent times.

### UBE2M reporter assay

To exclude any effects at the transcriptional or protein synthesis levels, we transfected HAP1 WT and *NAA30*-KO cells with a bicistronic P2A reporter vector allowing expression of both UBE2M-V5 and GST-GFP from a *CMV* promoter. HAP1 cells were seeded the day before and incubated overnight. HAP1 WT and *NAA30*-KO cells were transfected with 1.25 μg and 6.0 μg pUC-UBE2M-V5-P2A-GST-GFP, respectively. WT cells were co-transfected with an empty plasmid of similar size to obtain equal amounts of DNA in the transfection mixture. Transfections were performed using X-tremeGENE 9 DNA Transfection Reagent (Roche) (3:1 ratio of reagent to DNA) according to the manufacturer’s instructions. After 24 h, transfected cells were harvested and processed for immunoblotting. The transfections were performed four independent times. The immunoblot were probed three consecutive times with anti-V5 (1:5000), anti-GFP (1:5000), and finally anti-GAPDH (1:10000). Densitometry analysis was performed using Image Lab 6.0.1. Background-adjusted signal intensities of UBE2M-V5 were normalized to GST-GFP and expressed relative to WT. Data are shown as mean ± SD of four biologically independent experiments. Significance was determined using two-tailed unpaired *t* test.

### Immunoprecipitation for acetylation assays

HeLa cells were seeded at a density of 1.5 × 10^6^ cell per 10-cm dish and incubated overnight. The medium was replaced, and the cells were transfected with a mixture of 4 μg pcDNA3.1-NAA30-WT-V5, pcDNA3.1-NAA30-E321A-V5 or pcDNA3.1-LacZ-V5 (negative control), 12 μL X-tremeGENE 9 DNA Transfection Reagent (Roche) and 500 μL Opti-MEM (Gibco) according to the manufacturer’s instructions. After 24 h, the transfected cells were washed twice and scraped in cold PBS (Gibco) and collected by centrifugation at 1200 × *g* for 5 min at 4 °C. Cell pellets were lysed in 20 μL/mg IPH lysis buffer (50 mM Tris-HCl pH 8.0, 150 mM NaCl, 5 mM EDTA, 0.5% NP-40) with 1 × cOmplete EDTA-free protease inhibitor cocktail (Roche) on ice for 30 min. Cell debris was removed by centrifugation at 16,000 × *g* for 5 min at 4 °C and the supernatants were transferred to low-protein binding tube. ~40 μL cell lysate was saved for immunoblot analysis. For immunoprecipitation (IP), the cell lysates were incubated with 2 μg anti-V5 antibody per dish (Invitrogen, Cat # R960CUS) for 3 h at 4 °C on a rotating wheel. Then, 20 μL of pre-washed Dynabeads Protein G per dish (Invitrogen) was added to each IP sample, which were incubated overnight at 4 °C to retrieve the immunocomplexes. The beads were washed twice in IPH lysis buffer, once in acetylation buffer (50 mM HEPES pH 8.5, 100 mM NaCl, 1 mM EDTA) and resuspended in 40 μL acetylation buffer per dish. The IP samples were used in N-terminal acetylation assays (see below) and immunoblotting. One transfected 10-cm dish was typically used per peptide in the acetylation assay. The immunoblots were probed with anti-V5 (Invitrogen, #R960CUS, 1:20000 dilution).

### In vitro N-terminal acetylation assay

The acetyltransferase activity of NAA30-WT-V5 and NAA30-E321A-V5 were determined using an in vitro N-terminal acetylation assay based on radiolabeled acetyl-CoA as previously described with minor modifications^130^. Briefly, 10 μL immunoprecipitated enzyme were mixed with 300 μM synthetic peptide and 50 μM [^14^C]-acetyl-CoA (PerkinElmer) in acetylation buffer (50 mM HEPES pH 8.5, 100 mM NaCl, 1 mM EDTA) to a final volume of 25 μL. The reactions were incubated for 1 h at 37 °C using a Thermoshaker at 1400 rpm. After incubation, the beads were isolated using a magnetic rack and the reactions were quenched by spotting 20 μL of the reaction mixture onto P81 phosphocellulose paper (Millipore, 10 mm × 10 mm). The filters were washed 3 × 5 min with 10 mM HEPES (pH 7.4), air dried on paper, and immersed in 5 mL Ultima Gold F scintillation mixture (PerkinElmer). Radioactivity was detected by a Tri-Carb 2900TR liquid scintillation analyzer (PerkinElmer). Disintegrations per minute (DPM) for each reaction, representing the product formation, were normalized to the amount of immunoprecipitated NAA30-V5 quantified from immunoblots. The experiment was performed three independent times with three technical replicates each. Reaction mixtures with immunoprecipitated V5-control plasmid were used as negative controls to assess background signal. Data are shown as mean ± SD from one representative experiment.

The peptides were custom-made by Innovagen AB (Lund, Sweden) or BioGenes (Berlin, Germany) to a purity of ≥95% and were dissolved in Ultrapure distilled water (Invitrogen). The 24-mer peptides contain seven unique amino acids at their N-terminus followed by the same 17 C-terminal residues (RWGRPVGRRRRPVRVYP). The C-terminal portion is derived from adrenocorticotropic hormone (ACTH) peptide sequence, but the lysines have been replaced with arginines to prevent possible lysine acetylation from interfering with the activity measurements. The following synthetic peptides were used: UBE2M UniProtKB: P61081 ([NH2] MIKLFSL RWGRPVGRRRRPVRVYP [COOH]), ARFRP1 UniProtKB: Q13795 ([NH2] MYTLLSG RWGRPVGRRRRPVRVYP [COOH]), UNC50 UniProtKB: Q53HI1 ([NH2] MLPSTSV RWGRPVGRRRRPVRVYP [COOH]), and β-actin UniProtKB: P60709 ([NH2] DDDIAAL RWGRPVGRRRRPVRVYP [COOH]) (see Supplementary Data 7).

### HAP1 NAA30 rescue assay

HAP1 cells were seeded out at a density of 2.5 × 10^6^ cells per 10-cm dish and incubated overnight. HAP1 WT cells were transfected with 5 μg empty V5-vector, and HAP1 *NAA30*-KO cells were transfected with 5 μg of empty V5-vector, 4 μg pcDNA3.1-NAA30-WT-V5 + 1 μg of empty V5-vector, or 5 μg pcDNA3.1-NAA30-E321A-V5. Transfections were performed using X-tremeGENE 9 DNA Transfection Reagent (Roche) (3:1 ratio of reagent to DNA) according to the manufacturer’s instructions. After 24 h, transfected cells were harvested and processed for immunoblotting. The experiment was performed three independent times.

### Inhibition of proteasomal and lysosomal protein degradation

HAP1 WT and *NAA30*-KO cells were seeded at a density of 2.5 × 10^6^ cells per 10-cm dish. After 24 h, the cells were treated with inhibitors of proteasomal (MG132 and bortezomib) and lysosomal (bafilomycin A, leupeptin and ammonium chloride) protein degradation for 6 h at 37 °C. Cells were washed twice and scraped in ice-cold PBS (Gibco), and the resulting cell pellets were stored at −80 °C until immunoblot processing. The final drugs concentrations were as follows: 10 μM MG132, 10 nM bortezomib (both from Calbiochem), 200 nM bafilomycin A, (MedChem Express), 100 μg/mL leupeptin, and 10 mM ammonium chloride (both from Sigma-Aldrich). DMSO (Sigma-Aldrich) was used as vehicle control. The experiment was performed three independent times.

### siRNA transfection

HAP1 WT and *NAA30*-KO cells were seeded at a density of 200,000 cells per 6-cm dish for proteomic analysis, 3-500,000 cells per 10-cm dish for immunoblotting, and 60,000 cells/well in a 6-well plate for flow analysis. After 24 h the medium was replaced, and the cells were transfected with 20 nM of ON-TARGETplus siRNA SMART pools using DharmaFECT1 (all from Horizon Discovery) according to the manufacturer’s instructions. A non-targeting siRNA pool (Cat # D-001810-10-05) was used as control treatment. The cells were harvested 72 h post-transfection. For lysosomal staining, the transfected cells were incubated with fresh medium containing 1 × LysoView 488 for 1 h at 37 °C and then processed for flow analysis. The experiments were performed at least three independent times.

The following ON-TARGETplus siRNA SMART pools were used: KCMF1 (56888) Cat # L-017900-01-0050, UBE2A (7319) Cat # L-009424-00-0005, UBE2B (7320) Cat # L-009930-00-0005, UBR1 (197131) Cat # L-010691-00-0005, UBR2 (23304) Cat # L-006954-00-0005, UBR4 (23352) Cat # L-014021-01-0005, and UBR5 (51366) Cat # L-007189-00-0005.

### XY-UBE2M peptide pulldown assay

HeLa cells were transfected with pcDNA6.2-hUBR4-V5-Lumio (7.5 × 10^5^ cells/10-cm, 4 μg plasmid DNA, 48 h) and HAP1 WT cells were transfected with pCDH-EF1-UBR4N-FLAG-T2A-copGFP or pCDH-EF1-UBR4C-FLAG-T2A-copGFP (2.5 × 10^6^ cell/10-cm, 6 μg plasmid DNA, 24 h) using X-tremeGENE 9 DNA Transfection Reagent (Roche) (3:1 ratio of reagent to DNA) according to the manufacturer’s instructions. The cells were washed and scraped in cold PBS, collected by centrifugation at 1000 × *g* for 5 min, and stored at −80 °C until further processing.

For the peptide binding assays we used a set of biotinylated 11-mer peptides derived from the N-terminal sequence of UBE2M (MIKLFSLKQQK(K-biotin)) bearing NatA type (AI-), NatB type (ME-), NatC/E/F type (MI/ML/MY/MF-), Arg/N-degron type 1 (RI-), or Arg/N-degron type 2 (FI-) N-terminal residues. Alternatively, we used a set of 11-mer Sindbid nsP4-derived peptides (X-IFSTIEGRTY(K-biotin)) where the N-terminal X residues was Arg, Phe or Gly^131^. The peptides were cross-linked to streptavidin-conjugated magnetic beads (Thermo Scientific) using 20 μg peptide to 20 μL beads diluted in ~500 μL 1 × TBST 0.01% (25 mM Tris pH 7.5, 150 mM NaCl, and 0.01% v/v Tween 20). The peptide-bead mixtures were incubated on a rotating wheel (20 rpm) for 2–3 h at 4 °C, and subsequently washed twice in TBST 0.01% and once in binding buffer (20 mM, HEPES pH 7.9, 200 mM KCl, 0.05% (v/v) Tween 20, 10% glycerol).

The cell pellets were resuspended in cold hypotonic buffer (10 mM HEPES pH 7.9, 10 mM KCl, and 1.5 mM MgCl_2_) supplemented with cOmplete protease inhibitor (Roche) and incubated on ice for 30 min. The cell suspensions were then subjected to five freeze-thaw cycles in liquid nitrogen followed by centrifugation at 16,000 × *g* for 5 min at 4 °C. Protein concentration was determined using the Pierce BCA protein assay kit (Thermo Scientific). The cell lysates were diluted 1:10 in binding buffer (20 mM, HEPES pH 7.9, 200 mM KCl, 0.05% (v/v) Tween 20, 10% glycerol) with protease inhibitor. Next, 300–500 μg of total protein was transferred to the peptide-conjugated beads and the mixtures were gently rotated overnight at 4 °C. The beads were washed three times with binding buffer, resuspended in 50 μL 1 × SDS sample buffer (Alfa Aesar) and incubated at 95 °C for 5 min, followed by SDS-PAGE and immunoblot analysis. The peptides were custom-made by Innovagen AB, Lund, Sweden to a purity of >95%, and was dissolved in UltraPure distilled water (Invitrogen). All peptides can be found in Supplementary Data 7. The experiments were performed at least three independent times.

### MS-based protein quantification of siUBR4 samples

HAP1 WT and *NAA30*-KO cells were transfected with 20 nM of ON-TARGETplus UBR4 siRNA SMART pool (Cat # L-014021-01-0005) or non-targeting control pool (Cat # D-001810-10-05) using DharmaFECT1 (all from Horizon Discovery) and harvested 72 h post-transfection. The cells were washed twice in ice-cold DPBS buffer (Gibco), detached by scraping in cold DBPS with 1 × cOmplete protease inhibitor (Roche) and collected by centrifugation 16,000 × *g* for 15 s at 4 °C. The centrifugation step was repeated once to remove as much DPBS as possible. The resulting cell pellets were flash frozen in liquid nitrogen and stored at −80 °C until further processing. There were a total of four samples with four replicates each (16 tubes in total).

Samples were prepared using the S-Trap protocol according to the manufacturer’s instructions (ProtiFi, Cat # C02-mini-40). Cells were lysed in 100 μL S-trap lysis buffer (5% SDS in 50 mM TEAB, pH 8.5) on ice. The cells were then sonicated 3 × 15 s (with 1 min incubations on ice) each and centrifuged at 16,000 × *g* for 10 min at 4 °C. The supernatant was collected, and the protein concentration was determined using the Pierce BCA protein assay kit (Thermo Scientific). From each sample, 100 μg of protein material was retrieved and the volume was adjusted with S-trap lysis buffer to 46 µL. Subsequently, proteins were reduced by addition of DTT (15 mM f.c.) and incubation for 15 min at 55 °C, followed by alkylation with iodoacetamide (30 mM f.c.) for 15 min in the dark. Next, 5 μL of 12% phosphoric acid (to a f.c. of 1.1%) was added. The pH of the samples was checked if this was <1. 350 µL of protein binding/wash buffer (90% methanol, 100 mM TEAB, pH 7.55) was added to the samples before loading them onto the S-Trap column. Columns were centrifuged for 30 s at 4000 × *g* at RT and the flow-through was discarded. The column was washed three times by addition of 400 µL of binding/wash buffer and centrifuged for 30 s at 4000 × *g*. The samples were centrifuged a final time for 1 min at 4000 × *g* to completely remove the remaining binding/wash buffer. To each column, 125 μL digestion buffer (trypsin in 50 mM TEAB in a 1/100 (w/w)) was added and incubated ON at 37 °C. Peptides were eluted in three steps: first, 80 μL elution buffer 1 (50 mM TEAB in ddH_2_O) was added and samples were centrifuged for 1 min at 4000 × *g*. Followed by an addition of 80 μL of elution buffer 2 (0.1% FA in ddH_2_O) and a second centrifugation step. Finally, 80 μL of elution buffer 3 (50% ACN, 0.1% FA in ddH_2_O) was added and samples were centrifuged a third time. Elution fractions were pooled and vacuum dried.

Samples were re-dissolved in 100 µL 100 mM TEAB with 10% ACN pH 8.5 and the peptide concentration was measured on Lunatic microfluidic device (Unchained Labs). For each sample, 50 µg of peptide material was retrieved, and the volume was adjusted to 100 µL. TMTpro 16-plex labels (Thermo Fisher Scientific, Cat # A44521) were thawed at RT, suspended in 20 µL ACN and vortexed vigorously. Each sample was labeled by adding 10 µL label and incubating for 1 h at RT (600 rpm) (see Supplementary Data 5 for an overview of sample labeling).

The reaction was quenched with 1:20 v:v 5% hydroxylamine (NH_2_OH) (f.c. 0.25%) for 15 min at RT (600 rpm) and the 16 fractions were ultimately combined. 100 µg of peptide material was retrieved and vacuum dried. The labeled peptides were re-dissolved in 100 µL 0.1% TFA and 1 µL 100% TFA was added to lower the pH before sample clean-up. Samples were desalted using OMIX C18 tips (Agilent Technologies, Cat # A57003100) according to the manufacturer’s instructions and vacuum dried. Peptides were re-dissolved in 100 µL solvent A (0.1% TFA in water/ACN (98:2, v/v)) and injected for fractionation by RP-HPLC (Agilent series 1200) connected to a Probot fractionator (LC Packings). Peptides were first loaded in solvent A on a 4 cm pre-column (made in-house, 250 µm internal diameter (ID), 5 µm C18 beads, Dr. Maisch) for 10 min at 25 µL/min and then separated on a 15 cm analytical column (made in-house, 250 µm ID, 3 µm C18 beads, Dr Maisch). Elution was done using a linear gradient from 100% RP-HPLC solvent A (10 mM ammonium acetate (pH 5.5) in water/ACN (98:2, v/v)) to 100% RP-HPLC solvent B (70% ACN, 10 mM ammonium acetate (pH 5.5)) in 100 min at a constant flow rate of 3 µL/min. Fractions were collected every min between 20 and 96 min and pooled every 12 min to generate a total of 12 samples for LC-MS/MS analysis. All 12 fractions were dried under vacuum in HPLC inserts and stored at −20 °C until further use.

MS/MS analysis was performed similarly as described for the enrichment of N-terminal peptides (acetylation proteomics) with some minor changes outlined below. Peptides were separated on an Ultimate 3000 RSLCnano system in-line connected to an Orbitrap Fusion Lumos mass spectrometer (Thermo Scientific) with a linear gradient reaching 55% MS solvent B after 85 min, 99% MS solvent B at 90 min, followed by a 10-min wash at 99% MS solvent B and re-equilibration with MS solvent A. Full-scan MS spectra (300–1500 m/z) were acquired at a resolution of 120,000 in the Orbitrap analyzer after accumulation to an AGC value of 400,000 with a maximum injection time of 50 ms. The most intense ions above a threshold value of 50,000 and a charge state ranging from 2 to 7, subjected to a dynamic exclusion of 60 s, were isolated in the quadrupole for fragmentation in the ion routing multipole at a normalized collision energy of 37% after accumulation of precursor ions at a target value of 100,000 with a maximum of 86 ms with an isolation width of 0.9 Th. The fragments were analyzed in the in the Orbitrap with a resolution of 50,000.

The generated MS/MS spectra were processed with MaxQuant (version 1.6.17.0) using the Andromeda search engine with default search settings, including a false discovery rate set at 1% on both the peptide and protein level. Spectra were searched against the sequences of the human proteins in the Swiss-Prot database (release January 2021). The enzyme specificity was set at trypsin/P, allowing for two missed cleavages. Variable modifications were set to oxidation of methionine residues and N-terminal protein acetylation. Carbamidomethylation of cysteine residues was put as fixed modification. To cope with the spectra that suffer from co-fragmentation, the precursor ion fraction (PIF) option was set to 75%. MS2-based quantification using TMTpro16plex labels was chosen as quantification method and a minimum ratio count of 2 peptides (both unique and razor) was required for quantification. Further data analysis was performed with the Perseus software (version 1.6.15.0) after loading the protein groups file from MaxQuant (load reporter ion intensities corrected per label). With Perseus, reverse proteins, proteins that are only identified by site and contaminants were removed. Samples were normalized by subtraction of the median per sample. Proteins with less than three valid values in at least one group were removed and missing values were imputed from a normal distribution around the detection limit. Then, a multiple sample t-test (one-way ANOVA, permutation-based FDR = 0.01, S0 = 0) was performed to detect enrichments in the different samples. Also, t-tests (FDR = 0.01 and S0 = 0.1) were performed between the different cell types. The mass spectrometry proteomics data have been deposited to the ProteomeXchange Consortium via the PRIDE partner repository^126^ with the dataset identifier PXD034410. Processed data are presented in Supplementary Data 5.

### WST-1 assay

Cell viability was determined using a colorimetric assay, based on the cleavage of WST-1 (Roche) to formazan dye by cellular mitochondrial dehydrogenases in viable cells. HAP1 cells were seeded in 96-well plates with a density of 5000 cells/well. After 24 h the cells were incubated with fresh medium supplemented with 10% (v/v) WST-1 reagent for 3 h at 37 °C. The absorbance of formazan dye was measured at 450 nm using a Tecan microplate reader. Samples with culture medium plus WST-1 and without cells were used as background control and were subtracted from the sample absorbance. Background subtracted values were expressed relative to WT. Data are shown as mean ± SD of three independent experiments with four technical replicates each. Significance was determined using one-way ANOVA with Dunnett’s correction.

### Flow cytometry analysis

Cell granularity, size, and lysosomal content were determined by flow cytometry analysis. HAP1 cells were seeded in 6-well plates with a density of 150,000 cells/well or 60,000 cells/well for siRNA treatment and incubated for 24 h or 96 h, respectively. For lysosomal analysis, cells were stained with LysoView 488 (Biotium) for 1 h at 37 °C according to the manufacturer’s protocol. Cells were detached using TrypLE Express (Gibco), washed with PBS, and resuspended in FACS buffer (PBS with 5% FBS). Cells were quantified on a LSRFortessa flow cytometer equipped with FACSDiva software version 9.0.1 (both from BD Biociences). Samples were run with a fluidics speed set to slow, giving an event rate of 200-400 events/sec. About 50,000 events were collected using a sequential gating strategy: 1) side scatter area (SSC-A) vs forward scatter area (FSC-A) were used to separate live cells from debris, and 2) forward scatter height (FSC-H) vs forward scatter area (FSC-A) was used to separate single cells from aggregates after gating the strategy was employed. Granularity and cell size was determined by the light scatter properties of the cells, using median SSC-A and median FSC-A. Lysosomal content was assessed by detecting fluorescence at 530/30 nm (median FITC). Gating strategy for lysosome staining with the LysoView 488 stain is described in Supplementary Fig. 14. The flow cytometry results were analyzed using FlowJo version 10.8.1 (BD Biosciences). Data are shown as mean ± SD of three independent experiments. Significance was determined using one-way ANOVA with Dunnett’s correction (NatC KO) or Šidák correction (siRNA).

### Immunofluorescence of human cells

HAP1 cells were seeded on 12-mm glass coverslips (Paul Marienfeld GmbH) in 24-well plate (20,000-35,000 cells/well) and incubated overnight. Cells were fixed with 4% (w/v) paraformaldehyde in PBS buffer supplemented with 4% (w/v) sucrose for 15 min, washed with PBS, permeabilized with 0.1% Triton X-100 for 10 min, washed in PBS, and blocked with blocking solution (8% BSA and 2% goat serum in PBS) for 1 h. Cells were incubated with rabbit anti-COX IV (Cell Signaling, 4850, 1:200) or mouse anti-p62/SQSTM1 (Santa Cruz, sc-28359, 1:200) diluted in blocking solution for 1 h at RT in the dark. Subsequently, cells were washed with PBS and incubated with Alexa Fluor 594 goat anti-rabbit (Jackson ImmunoResearch, 111-586-003, 1:100) or Alexa Fluor 488 goat anti-mouse (Jackson ImmunoResearch, 115-547-003, 1:100) overnight at 4 °C in the dark. Cells were washed four times in PBS for a total of 1 h followed by a final wash in water before the coverslips were mounted on a drop of ProLong antifade mountant with NucBlue stain (Invitrogen). Cells stained for COX IV were examined with a Zeiss Axiovert 200 M widefield fluorescence microscope equipped with a AxioCam HRm camera and a Plan-Neofluar 100×/1.30 Ph3 oil-immersion objective (Carl Zeiss, Germany). Images were processed using ImageJ/Fiji version 2.1.0/1.53c^132^. Around 200 cells per cell line were examined for mitochondrial morphology and categorized as either normal, fragmented, elongated or elongated + fragmented. Cells stained with anti-p62/SQSTM1 were examined using an Andor Dragonfly 500 confocal spinning disk fluorescence microscope (Oxford instruments) equipped with an iXon 888 Life EMCCD camera and a 100×/1.49 oil-immersion objective. All images were acquired with a laser gain of 300 and 50 ms exposure. The signal intensity of the p62 images (Alexa Fluor 488) was measured as total intensity per unit volume (intensity/μm^3^). The volume was determined by volume rendering the 3D stack images obtained utilizing an additional fluorophore from the same image (Alexa Fluor 594). Imaris Version 9.8.0 was used for image processing and the surface application was used for volume rendering. Ten images per cell line were used to quantify the p62 signal intensity in each cell line.

HAP1 cells intended for siRNA treatment were seeded on ibiTreat μ-slide 4-well (ibidi GmbH) and incubated overnight. Then, cells were transfected with UBR1, UBR2 and UBR4 siRNA (siUBRs) or non-targeting control siRNA (siCtrl) as described earlier (see siRNA transfection). Due to long incubation time from seeding to fixation (72 h post-transfection, 96 h post-seeding) cells were singularized by trypsinization (Gibco) 48 h post-transfection to increase spreading of the cells. Cells were prepared for immunofluorescent imaging as described earlier, using primary antibody rabbit anti-COX IV (Cell Signaling, 4850, 1:200) and Alexa 594-conjugated secondary antibody (Jackson ImmunoResearch, 111-586-003, 1:100) together with Rhodamine Phalloidin (Invitrogen, R415, 1:50). Samples were mounted by applying 4-5 drops of ibidi mounting medium with DAPI (ibidi GmbH) to each well. Cells were examined using an Andor Dragonfly 500 confocal microscopy (Oxford instruments) equipped with an iXon 888 Life EMCCD camera and a 100×/1.49 oil-immersion objective. Images were processed using IMARIS version 9.7.2. Cells were examined for mitochondrial morphology as earlier. Data are shown as mean ± SD of three independent experiments. Significance was determined using one-way ANOVA with Šídák’s correction. Around 200 cells per sample were examined.

### Fly work and crosses

Flies were raised under standard procedures. All strains used in this study are listed in Supplementary Data 8. All males used in this study resulted from different *Drosophila* crosses. Control males resulted from crosses between y^1^ females with OR males or with FM0/DP (1;Y) y^+^ males. *Naa30A* deletion males resulted from crosses between y^1^, *Naa30A*^*Δ*74^/FM0 females or w, y^1^, *Naa30A*^Δ74^/FM0 females with OR males, or between y^1^, *Naa30A*^Δ74^/FM0 females with FM0/DP (1;Y) y^+^ males. *Naa30A* deletion + genomic rescue resulted from crosses between y^1^, *Naa30A*^Δ74^/FM0 females or w^67*c*23^, y^1^, *Naa30A*^Δ74^/FM0 females with w^1118^;; gNaa30A-myc males. *Naa30A* deletion with Mhc-Gal4 males resulted from crosses between w^67*c*23^, y^1^, *Naa30A*^Δ74^/FM0 females with w;; Mhc-Gal4 males. *Naa30A* deletion with UAS-UbcE2M males resulted from crosses between w^67*c*23^, y^1^, *Naa30A*^Δ74^/FM0 females with w;; UAS-UbcE2M-3xHA males. *Naa30A* deletion males ubiquitously expressing UbcE2M resulted from crosses between w^67*c*23^, y^1^, *Naa30A*^*Δ74*^/FM0;; UAS-UbcE2M/TM3,sb females with w;; Da-Gal4 males. *Naa30A* deletion males expressing UbcE2M in muscles resulted from crosses between w^67*c*23^, y^1^, *Naa30A*^*Δ74*^/FM0;; UAS-UbcE2M/TM3,sb females with w;; Mhc-Gal4 males.

### Generation of mutants and transgenic flies

*Drosophila Naa30A* deletion was generated by imprecise excision of a P element, inserted in the 5’ UTR of the *Drosophila Naa30A* gene (CG11412). Briefly, the y^1^, P(EPgy2)Naa30A^EY10202^,w^67*c*23^ (BL16976) was crossed with PΔ2-399B, Sb/TM3, Ser transposase line. F1 male flies were balanced by crossing them with female virgins Df(1)pn38/FM0. F1 female flies, resulting from this second cross, were chosen as candidate deletions (white eye) and single-female crosses with males Df(1)pn38/FM0 were performed for stock balancing. Deletion sizes were determined by PCR and sequencing, using a forward primer 1 kb upstream (CAAGGAAAGTGGAGGAAGTGC) and a reverse primer 1.5 kb downstream (GGTATGTATCCCTCGCCAATG) of the 5’ end of CG11412. Nearly 100 lines were tested and the y1, *Naa30A*^*Δ74*^, w^67*c*23^ was selected. Removal of the w recessive marker to create the stock y1, *Naa30A*^*Δ74*^, was performed by recombination with the wild-type X chromosome from the Oregon-R (OR) strain.

For generation of *Drosophila* strains carrying a genomic fragment with the Naa30A gene, a fragment containing the genomic sequence of *Naa30A* franked by 1 kb pairs upstream and downstream of the 5’UTR and 3’UTR was synthesized by Genescript (Piscataway, NJ, USA). This fragment was sequenced and cloned into pCaSpeR2 in the PstI and EcoRI restriction sites to create the pCaSpeR2-gNaa30. Microinjection of pCaSpeR2-gNaa30 and selection of transfected strains was performed by BestGene (Chino Hills, CA, USA).

### *Drosophila* immunostaining and image analysis

Adult indirect flight muscles were prepared and stained as described in^133^. Briefly, dissected hemithoraxes were fixed for 30 min with 4% formaldehyde in PBST (PBS with 0.2% Triton X-100). Samples were then rinsed 4 times with PBST and blocked for 30 min at room temperature with 5% BSA in PBST. Primary antibodies used were mouse anti-mono- and polyubiquitinylated conjugates (Enzo Life Sciences, BML-PW8810, Clone FK2, 1:250) and rabbit anti-Mef2 (Gift from Dr. Eileen Furlong, 1:200) and were diluted in 5% BSA in PBST. Samples were then rinsed 4 times with PBST and incubated with secondary antibody and/or stains diluted in 5% BSA in PBST for 3 h at room temperature. The secondary antibodies used for visualization of ubiquitin conjugates and Mef2 were anti-mouse Alexa Fluor 488 (Thermo Fischer, A-11029, 1:1000) and the anti-donkey Alexa Fluor 555 (Thermo Fisher, A-31572, 1:200), respectively. For F-actin visualization, Alexa-conjugated Phalloidin (Thermo Fisher, A12379, 1:200) or TRITC-conjugated Phalloidin (Sigma-Aldrich, P1951, 1:200) was used. Samples were then rinsed 4 times and mounted in Vectashield antifade mounting medium (Vector Labs) and imaged using a Leica SP8 confocal microscope or a Zeiss LSM710 confocal microscope. Protein aggregates were counted using Fiji cell counter plugin^132^ and the number of aggregates were represented as the number of aggregates per area of muscle.

### Longevity assay

A total of at least 50 males per genotype were used in this assay. Males were kept at a density of 6–8 flies per vial distributed by 8 tubes kept in an incubator at 25 °C. Flies were transferred to fresh vials every 2^nd^ day, and viability was recorded at the time of vial transfer.

### Climbing assay

The negative geotaxis assays were used to identify adult climbing defects. Flies were collected 0–3 days following eclosion and separated into batches of 10 in vials containing standard food media and were allowed to recover from the CO_2_ anesthesia for at least 24 h. For the climbing assay, the flies from one vial were transferred to a test tube with 22 mm diameter with a line drawn at 8 cm from the bottom. Flies were tapped to the bottom to induce an innate climbing response. The number of flies that climbed 8 cm in 10 s and in 30 s was recorded. For each batch of 10 flies, the assay was repeated 10 times, allowing for 1 min rest period between each trial. Two independent biological replicas were tested per genotype with approximately 5 batches of 10 males for each replica.

### Classifying fly climbing ability

To select *Naa30A* deletion males with good climbing ability (best climbers) and poor climbing ability (worst climbers), 200 *Naa30A* deletion males with 0–3 days after pupae eclosion were collected, separated into batches of 20 flies in food vials and transferred to fresh vials every 2 days until reaching the age of 7–10 days. The flies from each vial were transferred to test tube used for the climbing assay. Flies were tapped to the bottom to induce an innate climbing response, and the 9 males with best climbing ability (best climbing) and the 11 males with worst climbing ability were selected.

### Copulation assay

The day before the assay, a single male (0–3 days old) and a single virgin wild-type (OR) female (4–6 days old) were introduced into a different food vial where they recovered from the CO_2_ anesthesia. After 24 h the male and female were pooled and the number of males that were able to initiate copula within the first 10 min, 15 min, 20 min, or 30 min were recorded. Two independent biological replicas were tested per genotype. To avoid interference of the yellow mutation in the copulation success all males were carrying the yellow gene in the Y chromosome.

### Male fertility

For measuring male fertility, a single male with 1–4 days were placed with five 5–7 days old wild-type (Oregon-R (OR)) females into vials containing standard food media. Flies were allowed to mate for 2 days and then transferred to a fresh vial and allowed to mate for another 2 days. A male was classified as fertile when emerging larvae were observed in any of those tubes after an additional 2-day incubation. Two independent biological replicas were tested per genotype.

### Flight assay

Flight assay was performed as previously described in^134^. Briefly, an acetate sheet vertically divided into five different regions was coated with vacuum grease and inserted into a 1 L graduated cylinder and 2- or 20-days old males (after pupae eclosion) were dispensed into the apparatus by gently tapping vials containing 20 males into a funnel placed on top of the graduated cylinder. Flies became stuck to the sheet where they landed. The sheet was removed, and the number of flies was counted in each of the five regions. The flight index was calculated as the weighted average of the region into which the flies landed. At least 100 flies of each genotype were tested.

### Reporting summary

Further information on research design is available in the Nature Portfolio Reporting Summary linked to this article.

### Supplementary information

Supplementary Information

Reporting Summary

Description of Additional Supplementary Files

Supplementary Data 1

Supplementary Data 2

Supplementary Data 3

Supplementary Data 4

Supplementary Data 5

Supplementary Data 6

Supplementary Data 7

Supplementary Data 8

Supplementary Movie 1

Supplementary Movie 2

Supplementary Movie 3

### Source data

Source Data

## Data Availability

The CRISPR screen sequencing data have been deposited in NCBI’s Gene Expression Omnibus (Edgar et al., 2002) and are accessible through GEO Series accession number GSE221447. Mass spectrometry proteomics data have been deposited to the ProteomeXchange Consortium (http://proteomecentral.proteomexchange.org) via the PRIDE partner repository (Perez-Riverol et al., 2019) with the following dataset identifiers: N-terminal acetylome analysis of HAP1 WT and NatC KO cells PXD034992, LFQ shotgun proteomic analysis of HAP1 WT and NatC KO cells PXD034104, and TMT analysis of HAP1 WT siCtrl, WT siUBR4, *NAA30*-KO siCtrl, and *NAA30*-KO siUBR4 PXD034410. Processed data are presented in Table 1 and provided in Supplementary Data 3-5. Proteomics analysis of immunoprecipitated Naa30A-myc from *Drosophila* embryos is presented in Supplementary Data 6. Original immunoblot images and other source data are provided in Source Data File. Source data are provided with this paper.
