# Supplementary material for: N-terminal acetylation shields proteins from degradation and promotes age-dependent motility and longevity
Source: Nat Commun. 2023 Oct 27;14:6774. doi: 10.1038/s41467-023-42342-y (PMC10611716; doi:10.1038/s41467-023-42342-y)
Supplement: Supplementary file 1 — Supplementary Information [file 41467_2023_42342_MOESM1_ESM.pdf]

Supplementary Information

N-terminal acetylation shields proteins from degradation and promotes age-dependent motility and longevity

Sylvia Varland[1,2,3,19], Rui Duarte Silva[4,5,19], Ine Kjosås[1], Alexandra Faustino[4], Annelies Bogaert[6,7], Maximilian Billmann[8,9], Hadi Boukhatmi[10], Barbara Kellen[4], Michael Costanzo[3], Adrian Drazic[1], Camilla Osberg[1], Katherine Chan[3], Xiang Zhang[8], Amy Hin Yan Tong[3], Simonetta Andreazza[11], Juliette J. Lee[11], Lyudmila Nedyalkova[3], Matej Ušaj[3], Alexander J. Whitworth[11], Brenda J. Andrews[3,12], Jason Moffat[3,12,13], Chad L. Myers[8,14], Kris Gevaert[6,7], Charles Boone[3,12,15], Rui Gonçalo Martinho[4,16,17,20], and Thomas Arnesen[1,2,18,20]

Author affiliations

[1]Department of Biomedicine, University of Bergen, N-5021 Bergen, Norway

[2]Department of Biological Sciences, University of Bergen, N-5006 Bergen, Norway

[3]The Donnelly Centre for Cellular and Biomolecular Research, University of Toronto, Toronto, ON M5S 3E1, Canada

[4]Algarve Biomedical Center Research Institute, Universidade do Algarve, 8005-139 Faro, Portugal

[5]Faculdade de Medicina e Ciências Biomédicas, Universidade do Algarve, 8005-139 Faro, Portugal.

[6]VIB-UGent Center for Medical Biotechnology, B-9052 Ghent, Belgium

[7]Department of Biomolecular Medicine, Ghent University, B-9052 Ghent, Belgium

[8]Department of Computer Science and Engineering, University of Minnesota-Twin Cities, Minneapolis, MN 55455, USA

[9]Institute of Human Genetics, University of Bonn, School of Medicine and University Hospital Bonn, D-53127 Bonn, Germany

[10]Institut de Génétique et Développement de Rennes (IGDR), Université de Rennes 1, CNRS, UMR6290, 35065 Rennes, France

[11]MRC Mitochondrial Biology Unit, University of Cambridge, Cambridge, CB2 0XY, UK

[12]Department of Molecular Genetics, University of Toronto, Toronto, ON M5S 3E1, Canada

[13]Present address: Program in Genetics & Genome Biology, The Hospital for Sick Children, Toronto, ON M5G 1X8, Canada

[14]Bioinformatics and Computational Biology Graduate Program, University of Minnesota-Twin Cities, Minneapolis, MN 55455, USA

[15]RIKEN Centre for Sustainable Resource Science, Wako, Saitama 351-0106, Japan

[16]Departmento de Ciências Médicas, Universidade de Aveiro, 3810-193 Aveiro, Portugal

[17]iBiMED - Institute of Biomedicine, Universidade de Aveiro, 3810-193 Aveiro, Portugal

[18]Department of Surgery, Haukeland University Hospital, N-5021 Bergen, Norway

[19]These authors contributed equally

[20] These authors jointly supervised this work

[*]**Correspondence**: sylvia.varland@gmail.com (S.V.), rfdsilva@gmail.com (R.D.S.), rgmartinho@ua.pt (R.G.M.), and thomas.arnesen@uib.no (T.A.).

Supplementary Figures

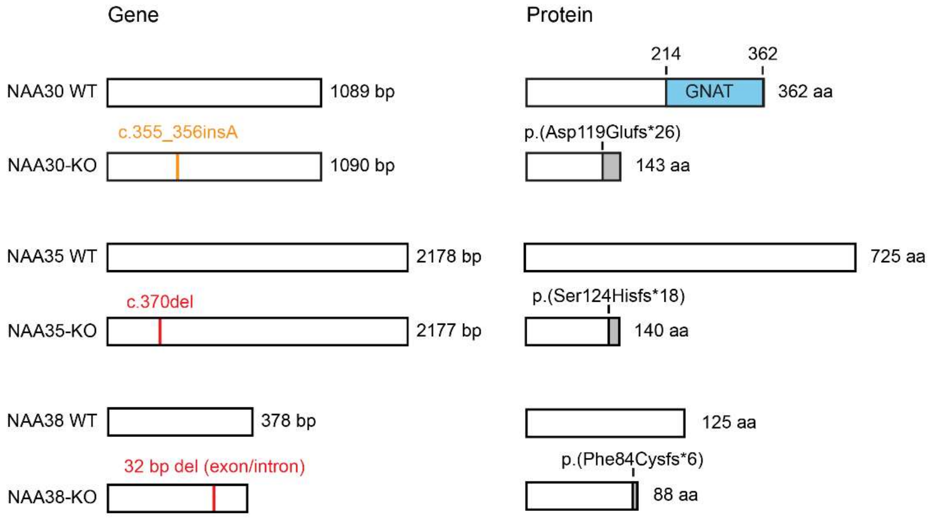

Supplementary Fig. 1: Schematic representation of HAP1 NatC KO cell lines

HAP1 *NAA30*-KO, *NAA35*-KO and *NAA38*-KO cell lines were generated using CRISPR-Cas9 mediated genome editing. *NAA30*-KO cells have a 1 bp insertion in exon 2 (c.355_356insA) of the *NAA30* gene (Gene ID: 122830; CCDS32088). *NAA30*-KO cells could produce a putative protein product of 143 aa (p.(Asp119Glufs*26)) based on UniProt ID: Q147X3-1, which would be missing the catalytic GNAT domain (blue) containing the acetyl-CoA binding motif. *NAA35*-KO cells have a 1 bp deletion in exon 6 (c.370del) of the *NAA35* gene (Gene ID: 60560; CCDS 6673) and could generate a putative protein product of 140 aa (p.(Ser124Hisfs*18)) based on UniProt ID: Q5VZE5-1. *NAA38*-KO cells have a 32 bp deletion in the exon 1/intron border (NC_000017.11:g.7857000_7857031del) of the *NAA38* gene (Gene ID 83316; CCDS 11122). *NAA38*-KO cells could produce a putative protein product of 136 aa (p.(Phe132Cysfs*6) based on UniProt ID: Q9BRA0-2. Schematic is not scaled to size. Deletion (red), insertion (orange), GNAT domain (blue), new C-terminal sequence after frameshift mutation (grey). WT: wild type; KO: knockout; bp: base pair; aa: amino acids.

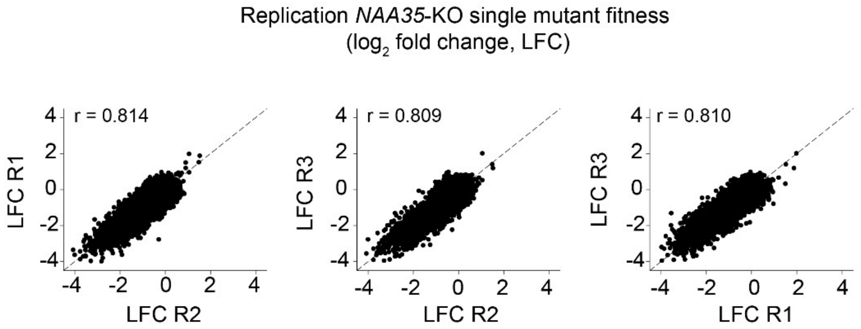

Supplementary Fig. 2: Fitness effects of independent *NAA35*-KO replicate screens are highly reproducible

Fitness effects were highly correlated across *NAA35* replicate screens. The reproducibility of the fitness effects (log_2-fold change, LFC) of three independent *NAA35* screens was determined by calculating the Pearson correlation coefficient (*r*) between all possible pairwise combinations.

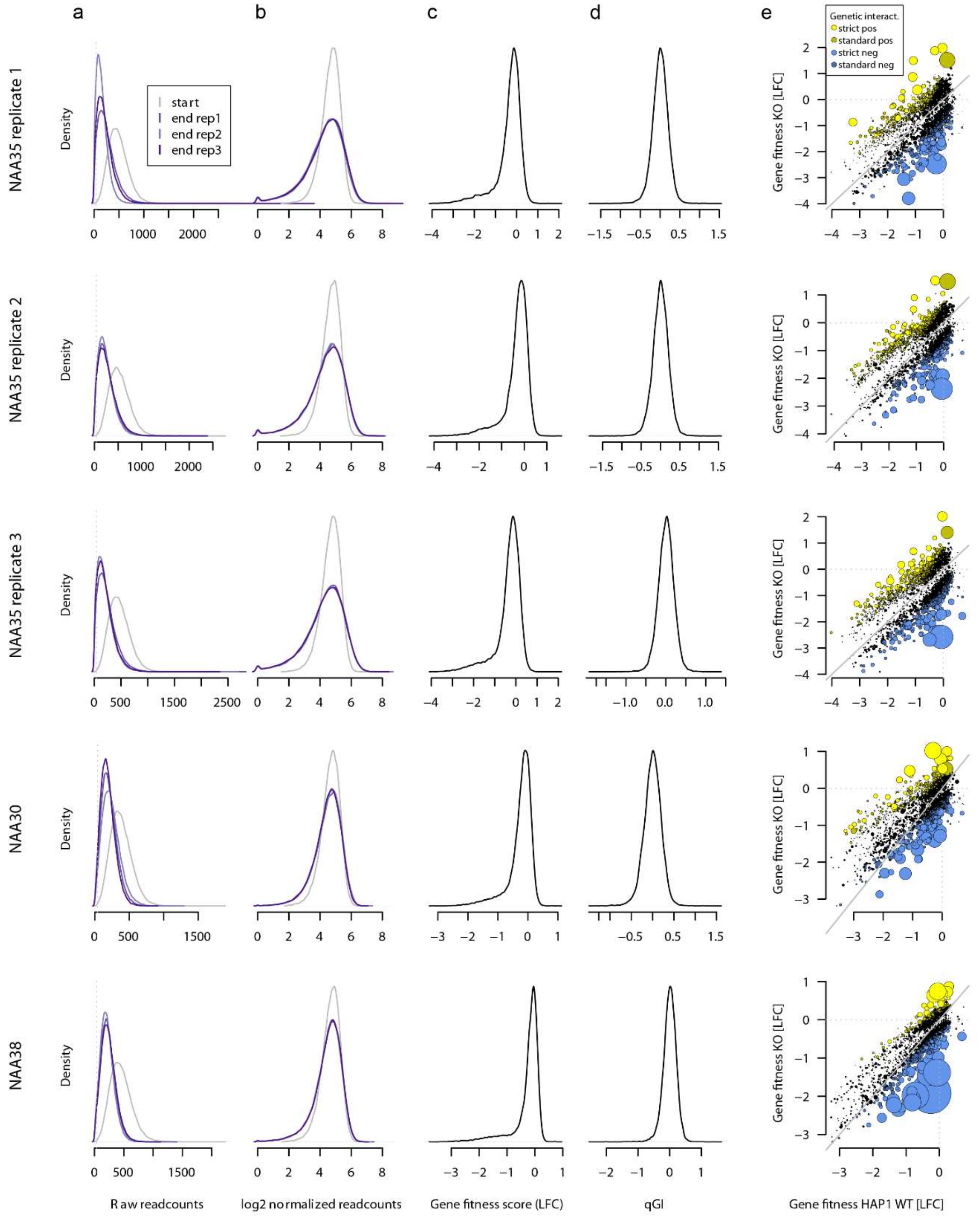

Supplementary Fig. 3: Readcount, LFC and qGI distributions for the *NAA35*-KO biological replicate screens, and *NAA30*-KO and *NAA38*-KO screens

(**a**) Guide RNA raw readcount distribution for genome-wide genetic interaction screens. Readcount distributions at screen start point (grey) and endpoint point for each technical replicate (shades of purple) are shown. The dotted line indicates minimum gRNA readcount

threshold. Guide RNAs with readcounts below this threshold at the screen start point were excluded from all analyses. (**b**) Log2-transformed and read depth normalized guide RNA readcount distribution for genome-wide genetic interaction screens. Readcount distributions at screen start point (grey) and endpoint point for each technical replicate (shades of purple) are shown. (**c**) Distribution of double mutant fitness scores (log2-fold change, LFC) derived from each genome-wide screen. (**d**) Distribution of quantitative genetic interaction (qGI) scores from each genome-wide screen. (**e**) A scatterplot illustrating the fitness effect (LFC) of genes in the indicated query mutant versus WT parental HAP1 cell line. Genes that exhibited a significant genetic interaction with the indicated query mutant at a standard confidence threshold (|qGI| > 0.3, FDR < 0.1) or a strict confidence threshold (|qGI| > 0.6, FDR < 0.01) are shown in different shades of blue (negative interactions) or yellow (positive interactions). Node size corresponds to the absolute qGI score.

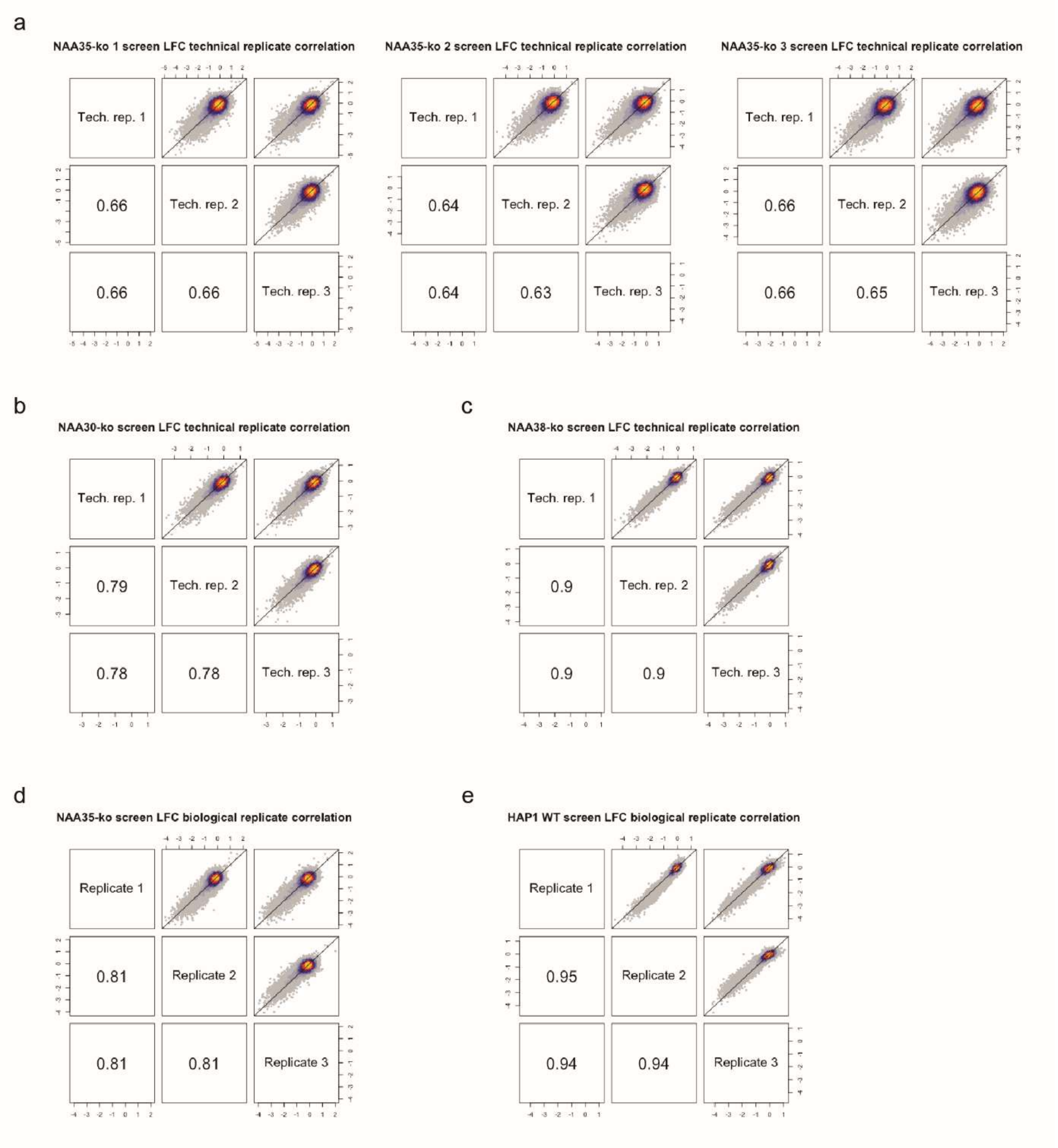

Supplementary Fig. 4: Pairwise scatterplots and correlation values for *NAA35*, *NAA30*, and *NAA38* pooled CRISPR screens

(**a**) Matrix of scatter plots illustrating the agreement (Pearson correlation coefficient, PCC) in gene level log2-fold change (LFC) values between all possible pairs of technical replicates in each of the 3 independent, genome-wide *NAA35* query mutant screens. Technical replicates refer to a single starting pool of cells that is split into 3 separate pools following lentiviral infection and puromycin selection. Biological replicates refer to three completely independent screens, and independent lentiviral infections. (**b-c**) Matrix of scatter plots illustrating the

agreement (PCC) in gene level LFC values between all possible pairs of technical replicates in the genome-wide *NAA30* query mutant and *NAA38* query mutant screen, respectively. (**d**) Matrix of scatter plots illustrating the agreement (PCC) in gene level LFC values between all possible pairs of independent, genome-wide NAA35 query mutant biological replicate screen (**e**) Matrix of scatter plots illustrating the agreement (PCC) in gene level LFC values between all possible pairs of three independent, genome-wide WT control screens.

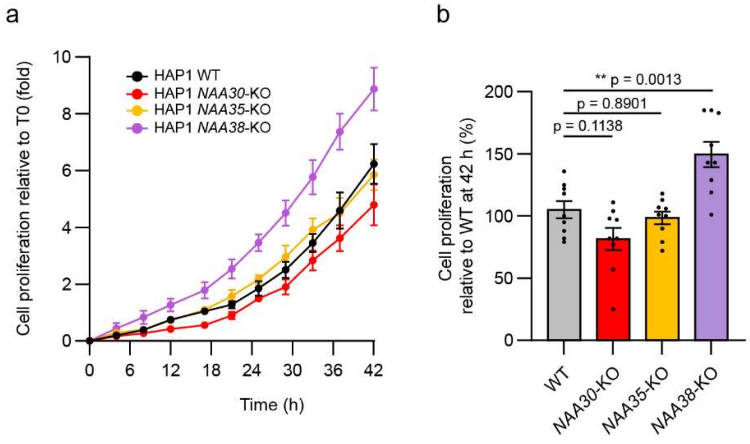

Supplementary Fig. 5: HAP1 *NAA38*-KO cells have increased cell proliferation

HAP1 WT and NatC KO cells were monitored in a HoloMonitor M4 imaging system for 42 h with image acquisition every 10 min. Single cell data in each experiment was obtained from one randomly selected field of view from three individual wells (n = 3). **(a)** Cell proliferation curves showing fold increase in cell number relative to 0 h (T0). Data are shown as mean ± SEM of three independent experiments pooled together (n = 3). **(b)** Cell proliferation was quantified by comparing relative increase in cell numbers between 0 and 42 h. Data are shown as mean ± SEM of three independent experiments expressed relative to WT. Mean cell proliferation WT = 105%, *NAA30*-KO = 82%, *NAA35*-KO = 98 %, and *NAA38*-KO = 149%. n = 9. **p = 0.0013 one-way ANOVA with Dunnett's correction. ns: not significant. Source data are provided as a Source Data file.

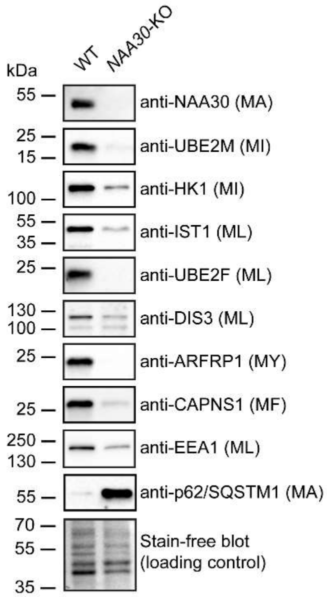

Supplementary Fig. 6: Human NAA30 protects NatC substrates from proteostasis in the breast cancer cell line MDA-MB-231

Immunoblot analysis of putative and previously confirmed NatC substrates using whole cell lysate from the human breast cancer cell line MDA-MB-231 WT and *NAA30*-KO (see **Fig. 2** for similar analyses in HAP1 cells). The endogenous proteins levels of the following NatC substrates were examined (the two first amino acids are indicated in parenthesis): UBE2M (MI), HK1 (MI), IST1 (ML), UBE2F (ML), DIS3 (ML), ARFRP1 (MY) and CAPNS1 (MF). The putative NatC substrate EEA1 (ML) and NatC-affected protein p62/SQSTM1 (MA) (referring to **Fig. 5e-f**) were also examined. Gene disruption in the *NAA30*-KO cell line was verified using anti-NAA30 antibody. WT refers to a paired unmodified control cell line that has been through the CRISPR/Cas9 procedure. n = 3 biologically independent samples. Source data are provided as a Source Data file.

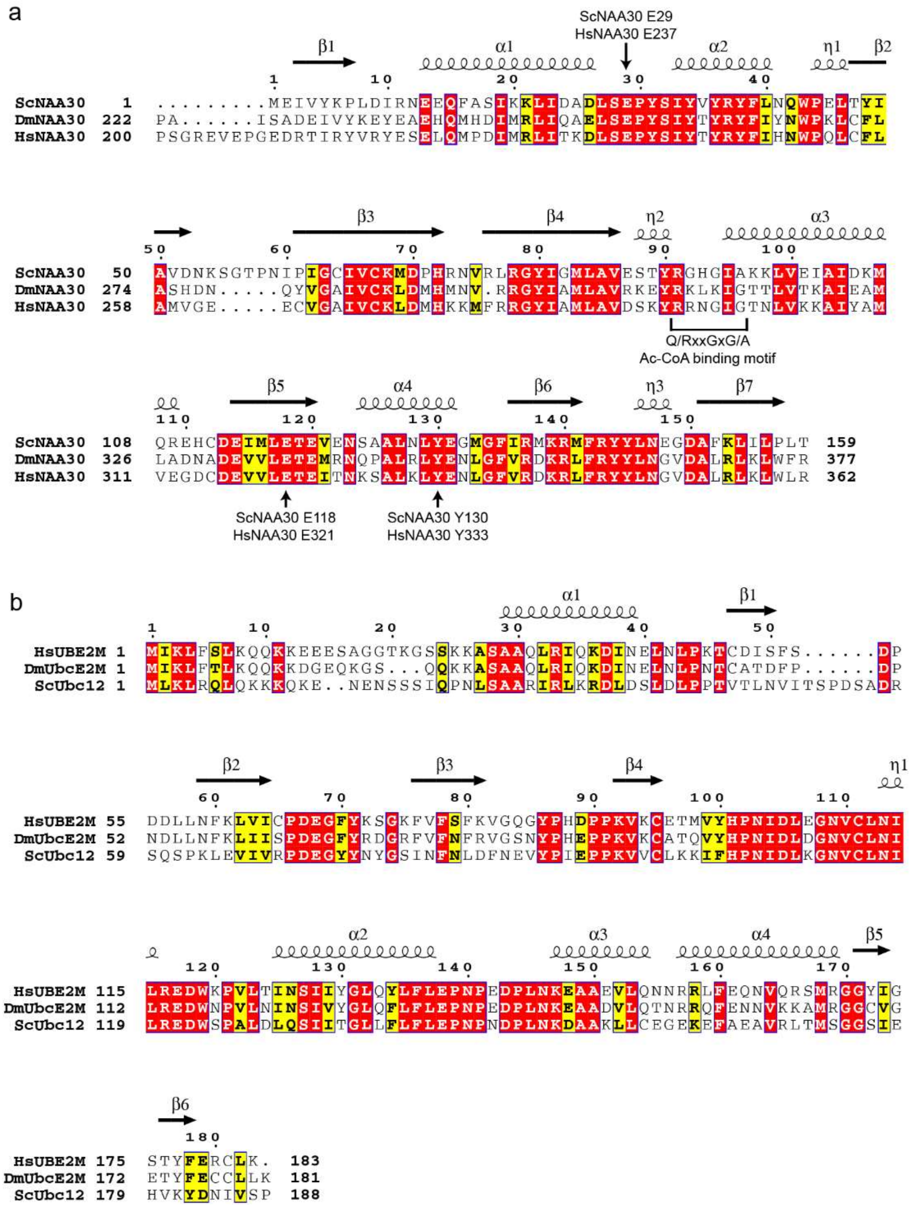

Supplementary Fig. 7: Multiple sequence alignment of NAA30 and UBE2M/UbcE2M/UBC12

Multiple sequence alignment of **(a)** NAA30 and **(b)** UBE2M/UbcE2M/UBC12 orthologs from *Homo sapiens* (Hs), *Drosophila melanogaster* (Dm) and *Saccharomyces cerevisiae* (Sc). Secondary structures were predicted from yeast NatC structure (PDB: 6YGA)[1] and human UBE2M (PDB: 1Y8X)[2]. Completely and highly conserved amino acids are shown in red and yellow boxes, respectively. Sequence alignment was performed using Clustal Omega, and secondary structures were visualized using ESPript 3.0 The acetyl-CoA binding motif

(Q/RxxGxG/A) and residues Glu29, Glu118, and Tyr130, which are crucial for *in vitro* ScNAA30 activity, are indicated. Source data are provided as a Source Data file.

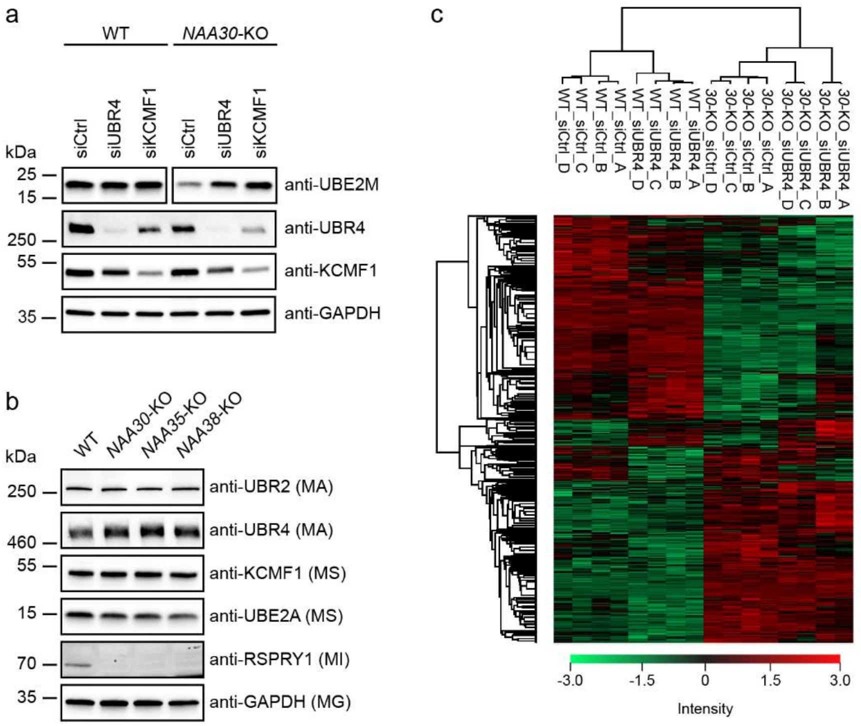

Supplementary Fig. 8: Knockdown of UBR4 partly restores UBE2M protein levels

(a) Immunoblot analysis of endogenous UBE2M, UBR4 and KCMF1 protein levels in HAP1 WT and *NAA30*-KO cells transfected with the indicated siRNAs for 72 h **(b)** Immunoblot analysis of proteins representing positive GIs of NatC using total cell extract from HAP1 WT and NatC KO cells. (**a-b**) n = 3 biologically independent experiments. **(c)** Heat map representation of proteins that were identified as significantly differentially regulated by UBR4 knockdown in at least 3 of 4 measurements and that passed an multiple sample test (one-way ANOVA, permutation-based FDR = 0.01, S0 = 0) referring to **Methods** and **Supplementary Data 5**. Source data are provided as a Source Data file.

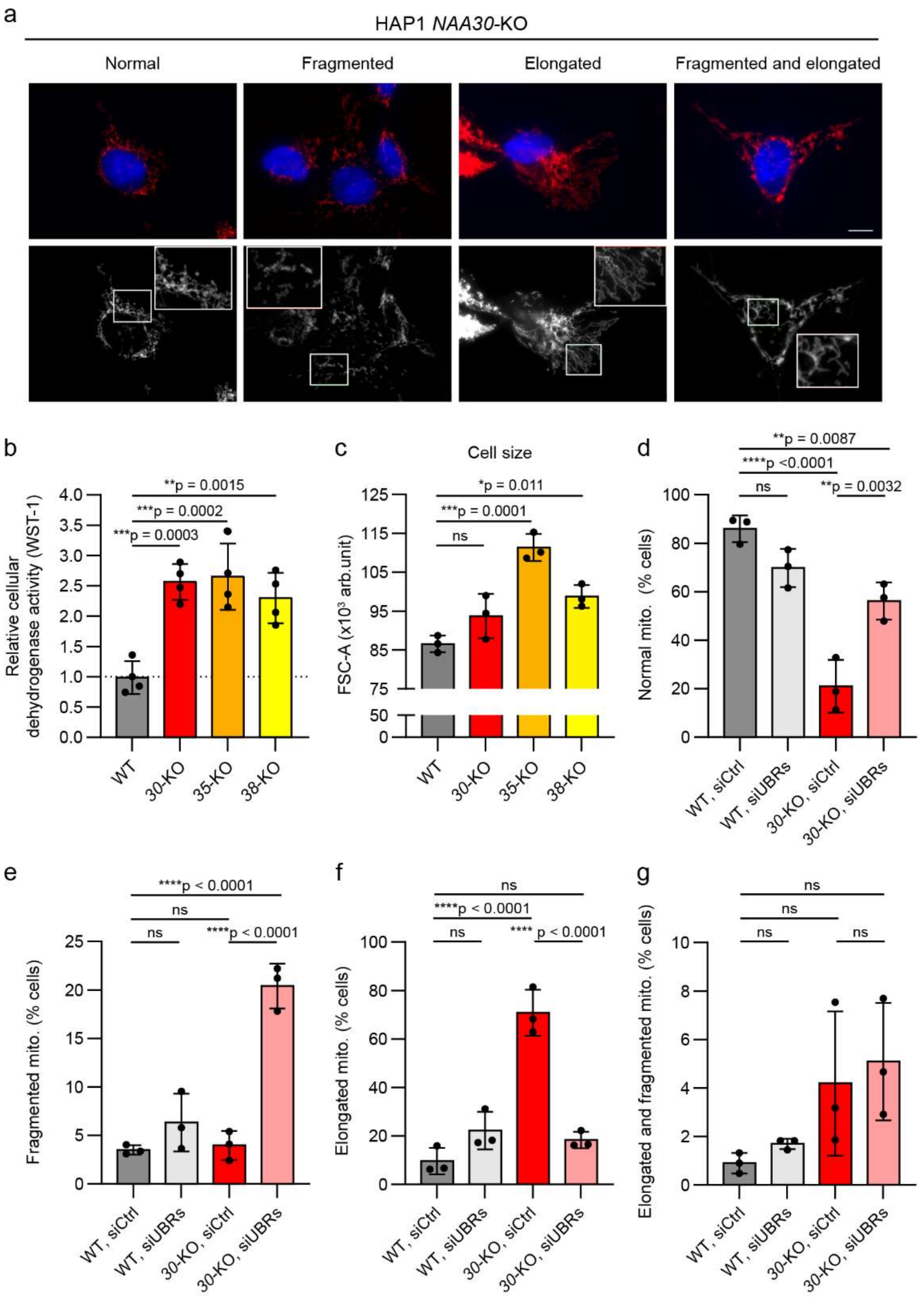

Supplementary Fig. 9: NatC is required for normal mitochondrial phenotype, dehydrogenase activity and cell size

(a) Loss of NatC leads to elongated and fragmented mitochondria. HAP1 WT and NatC KO cells were stained with the mitochondrial marker anti-COX IV (red) and the nuclear marker NucBlue (blue) and analyzed by immunofluorescence. Upper panel: Representative

micrographs showing the different mitochondrial phenotypes observed in HAP1 *NAA30*-KO cells. Lower panel: Same micrographs with zoom-in-frames of mitochondria. Cells were grouped into four bins based on mitochondrial morphology: normal, fragmented, elongated, and elongated + fragmented, see **Fig. 5a**. At least 200 cells per cell line from one experiment were examined. Scale bar, 10 µm. **(b)** NatC KO cells have increased cellular dehydrogenase activity. Cell viability of HAP1 cells was measured by WST-1 staining 24 h post-seeding. The WST-1 assay relies on cellular mitochondrial dehydrogenase activity. Data are shown as mean ± SD of four independent experiments with four technical replicates expressed relative to WT. **p = 0.0015, ***p ≤ 0.0003; one-way ANOVA with Dunnett's correction. **(c)** NatC KO cells show increased cell size. Median forward scatter area (FSC-A) indicating cell size were determined by flow cytometry. Data are shown as mean ± SD of three independent experiments. *p = 0.011, ***p < 0.0001; one-way ANOVA with Dunnett's correction. (*NAA30*-KO, p = 0.1171). **(d-g)** UBR knockdown in *NAA30*-KO cells rescues the abnormal mitochondrial phenotype. HAP1 WT and *NAA30*-KO cells were transfected with siCtrl or siUBR1, siUBR2 and siUBR4 (siUBRs), immunostained with anti-COX IV and analyzed by immunofluorescence. Cells were grouped into four bins based on mitochondrial morphology: **(d)** normal, **(e)** fragmented, **(f)** elongated, and **(g)** elongated + fragmented. Data are shown as mean ± SD of three independent experiments (~200 cells per sample). **p ≤ 0.0087, ****p < 0.0001; one-way ANOVA with'Šídák's correction. See combined data in **Fig. 5h**. ns: not significant. Source data are provided as a Source Data file.

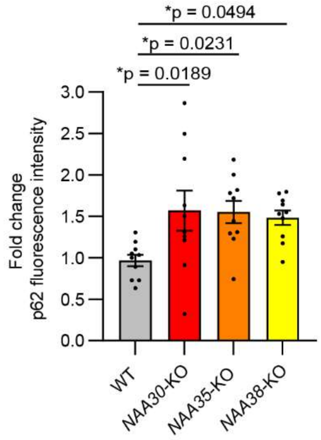

Supplementary Fig. 10: HAP1 NatC KO cells have increased p62/SQSTM1 protein levels

HAP1 cells were stained with anti-p62/SQSTM1 and examined with immunofluorescence microscopy (representative micrographs shown in **Fig. 5e**). The fluorescent intensity of p62 per cell (intensity/μm^3) where quantified in each cell line and is shown as mean ± SD of one experiment (n = 10 cells). *p ≤ 0.05; one-way ANOVA with Dunnett's multiple comparisons test. Source data are provided as a Source Data file.

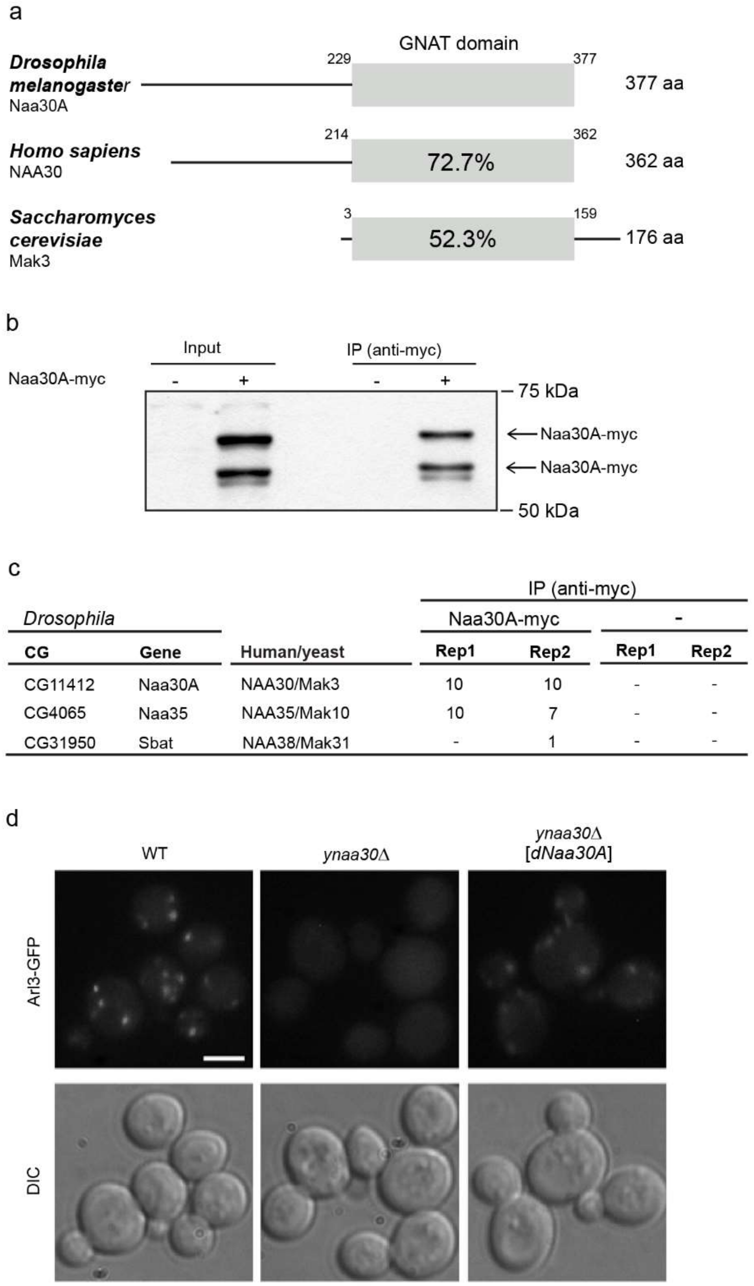

Supplementary Fig. 11: Naa30A/CG11412 is the catalytic subunit of *Drosophila* NatC

(a) Schematic representation of *Drosophila melanogaster* Naa30A (also known as CG11412), and its orthologs *Homo sapiens* NAA30 and *Saccharomyces cerevisiae* Mak3. Naa30A has a GNAT domain with an identity of 72.7% and 52.3% compared to human NAA30 and yeast Mak3, respectively. **(b)** Immunoprecipitation of Naa30A-Myc from *Drosophila* embryos total protein extracts. Immunoprecipitation using an anti-Myc antibody and embryos with 0 to 4 hours after egg laying. Embryos were collected from females expressing UAS-Naa30A-myc

under the control of the nanos-Gal4 driver. The control embryos with 0 to 4 hours after egg laying were collected from wild-type (Oregon R; OR) females. Two different protein isoforms corresponding to Naa30A-Myc could be detected by immunoblotting using anti-Myc antibody (arrows). **(c)** *Drosophila* Naa30A interacts with distinct orthologs of known auxiliary subunits of yeast and human NatC complexes. Number of unique peptide sequences identified by LC-MS/MS analysis of immunoprecipitated *Drosophila* Naa30A complexes from embryos expressing Naa30A-Myc and control wild-type embryos (no Myc-tagged Naa30A). The complete list of proteins co-immunoprecipitated with Myc-tagged Naa30A is shown in **Supplementary Data 6**. **(d)** *Drosophila* Naa30A rescues Arl3-GFP localization phenotype in yeast Naa30/Mak3 deletion strain. Arl3-GFP localization in wild-type yeast cells, *naa30Δ* yeast cells and *naa30Δ* yeast cells expressing *Drosophila* Naa30A. Scale bar, 2 µm. Source data are provided as a Source Data file.

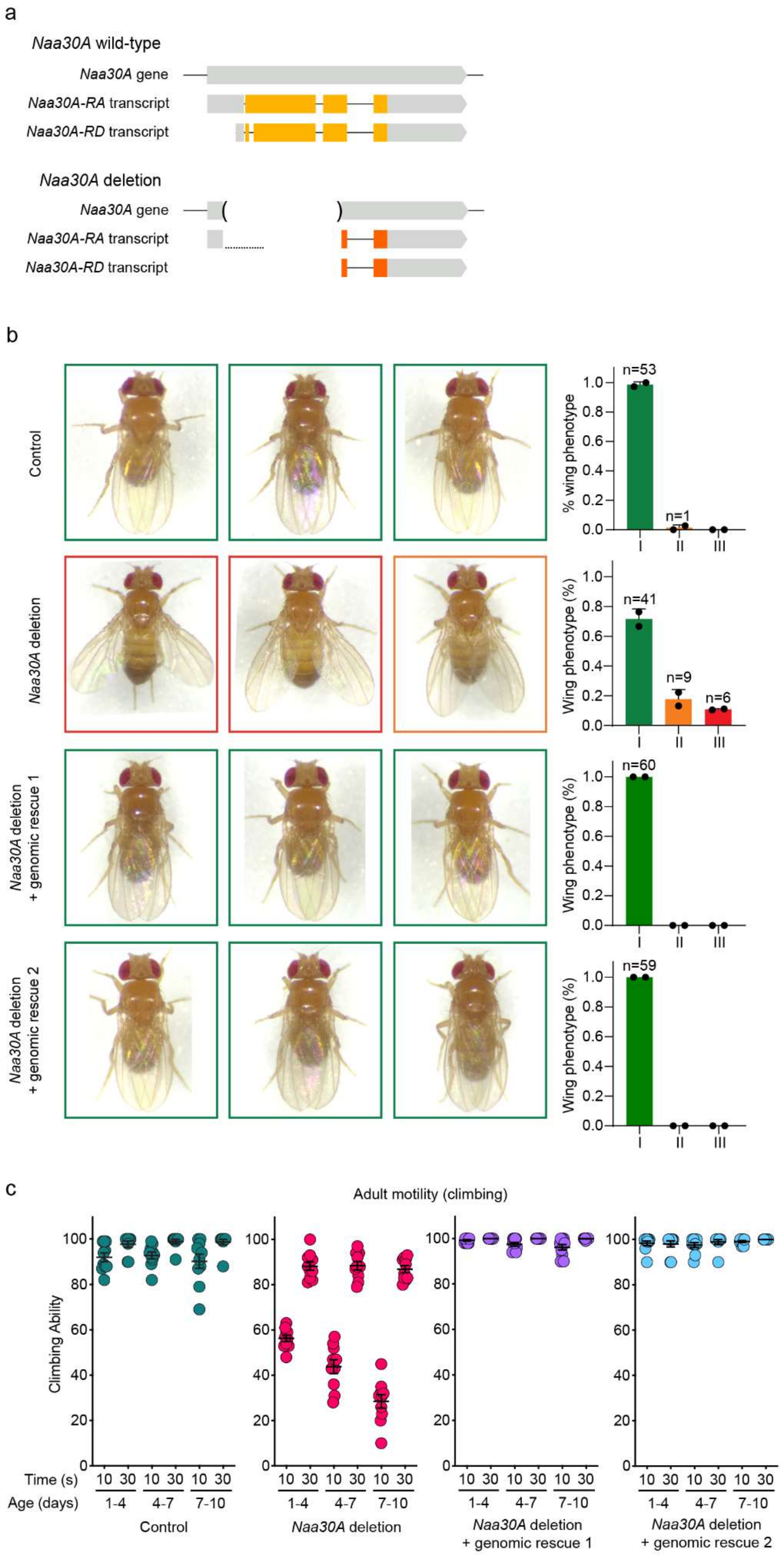

a
Naa30A wild-type
Naa30A gene
Naa30A-RA transcript
Naa30A-RD transcript
Naa30A deletion
Naa30A gene
Naa30A-RA transcript
Naa30A-RD transcript
b
Control
Naa30A deletion
Naa30A deletion + genomic rescue 1
Naa30A deletion + genomic rescue 2
% wing phenotype
Wing phenotype (%)
n=53
n=1
n=41
n=9
n=6
n=60
n=59
I
II
III
c
Adult motility (climbing)
Climbing Ability
Time (s)
Age (days)
1-4
4-7
7-10
Control
Naa30A deletion
Naa30A deletion + genomic rescue 1
Naa30A deletion + genomic rescue 2

Supplementary Fig. 12: Schematic representation and analysis of the held-out wings and mobility phenotypes of *Naa30A* deletion flies

(a) Top: Schematic representation of the wild-type *Drosophila Naa30A* gene and the two annotated unique transcripts, Naa30A-RA and Naa30A-RD. Bottom: Schematic representation of the *Naa30A* deletion of the first 302 amino acids (almost all coding sequence), with an additional frameshift mutation within the remaining coding sequence and a stop codon in the newly generated position 20. **(b)** *Drosophila Naa30A* deletion males exhibit a held-out wing phenotype. This phenotype can be rescued by a genomic *Naa30A* construct inserted at two distinct loci in the genome**.** Representative images of control, *Naa30A* deletion males, and *Naa30A* deletion males carrying a genomic rescue inserted at two distinct loci in the genome: genomic rescue 1 and genomic rescue 2. These images demonstrate the presence of a held-out wing phenotype in *Naa30A* deletion flies 7-10 days after pupae eclosion that is rescued by both genomic rescues constructs. The held-out wing phenotype was classified into three classes for scoring: class 1 representing normal wings, class 2 indicating a weak held-out wing phenotype, and class 3 representing a strong held-out wing phenotype. To illustrate the scoring criteria each image is depicted with a distinct color frame, where class 1 is green, class 2 is orange, and class 3 is red. Quantification for the different phenotypes are shown on right graph and are the average of two independent biological replicas. *n* represents the total number of flies scored in both replicas. **(c)** *Naa30A* deletion males show an accelerated age-dependent loss of motility. This phenotype is rescued by a genomic *Naa30A* construct inserted at two distinct loci in the genome (genomic rescue 1 and genomic rescue 2)**.** Climbing ability was used as a measure of mobility and represents the percentage of males that were able to climb 8 cm after 10 and 30 s. Control males, *Naa30A* deletion males, and *Naa30A* deletion males carrying a *Naa30A* genomic rescue construct at the ages of 1-4 days, 4-7 days, and 7-10 days after pupae eclosion were used. Results were obtained with males collected from two independent biological replicas. Each dot represents the average climbing ability of a group of 10 males after 10 technical replicates, mean ± SEM is also indicated. *Naa30A* deletion males exhibited a significant reduction in their climbing ability as they aged (1-4 days old males vs 4-7- or 7-10-days old males; $p<0.001$; one-way ANOVA with Šídák's multiple comparisons test). Genotypes of the males used: **(b-c)** control males (y^1/Y), *Naa30A* deletion males (y^1, Naa30A$^{\Delta 74}$/Y), *Naa30A* deletion males with the *Naa30A* genomic rescue 1 (y^1, Naa30A$^{\Delta 74}$/Y; +/+; gNaa30A-myc/+) and *Naa30A* deletion males with the *Naa30A* genomic rescue 2 (y^1, Naa30A$^{\Delta 74}$/Y; gNaa30A-myc/+; +/+). Source data are provided as a Source Data file.

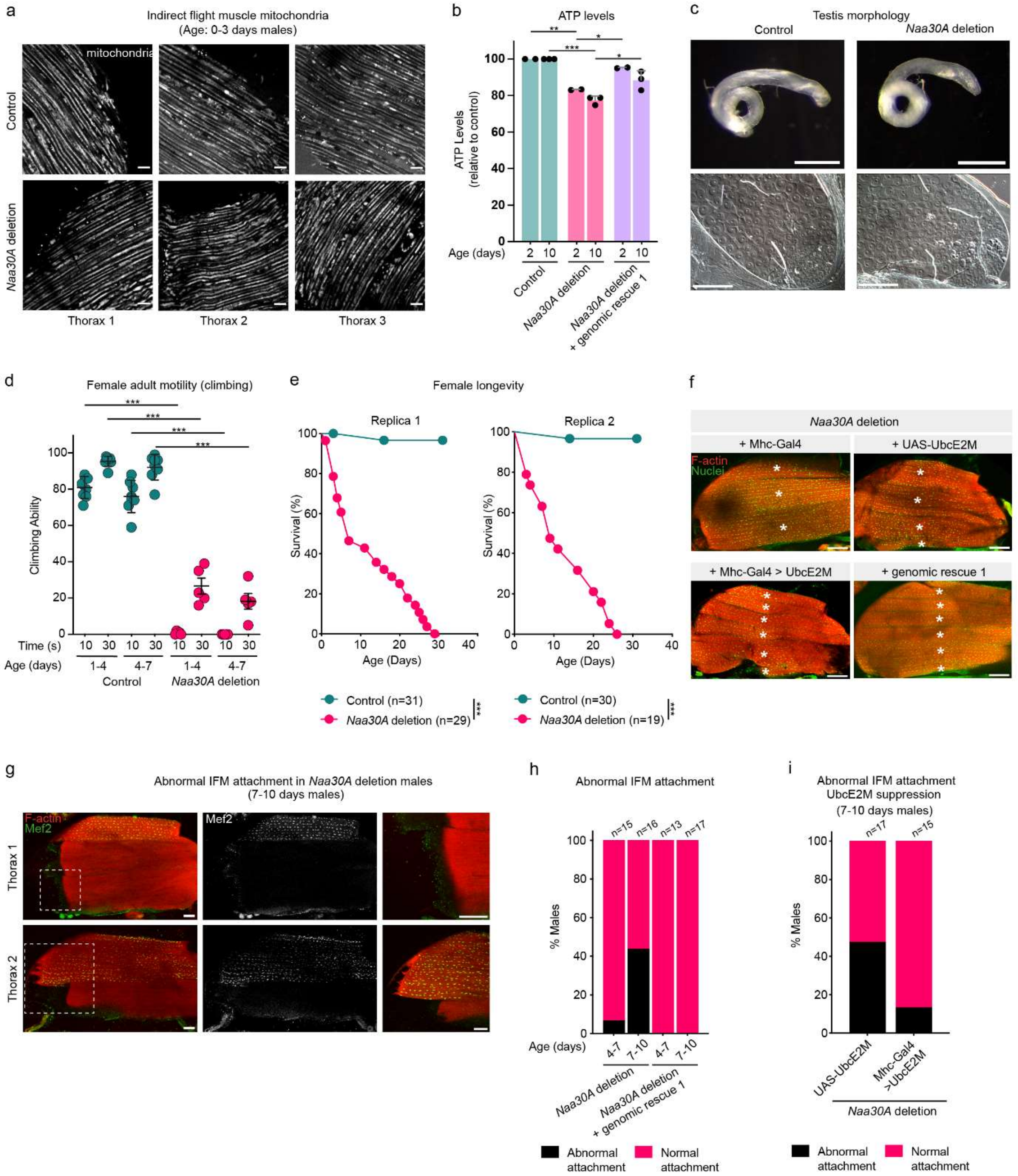

Supplementary Fig. 13: Multi-phenotypic analysis of *Naa30A* deletion: muscle mitochondrial network, ATP levels, testis morphology, sperm motility, female climbing ability, female longevity, and IFM number and attachment

(a) Muscle mitochondrial network is not obviously impaired after deletion of *Drosophila Naa30A*. Mitochondrial network in indirect flight muscles from young (1-4 days after pupae eclosion) control males or *Naa30A* deletion males labelled with UAS-mito-GFP expressed

under control of the ubiquitous daughterless-Gal4 (da-GAL4) driver. Results are shown for indirect flight muscles of three different thoraxes. Scale bar, 5 µm. **(b)** *Drosophila Naa30A* males have a reduction in ATP levels. Total ATP levels in young (2 days after pupae eclosion) and older (10 days after pupae eclosion) control males, *Naa30A* deletion males and *Naa30A* deletion males carrying the *Naa30A* genomic rescue 1. Results are mean ± SEM of two (young flies) or three (old flies) biological replicas. Statistical significance was assessed using the two-way ANOVA with Šídák's multiple comparisons test. **(c)** *Drosophila Naa30A* males have normal testis morphology. Top panel: Images of whole testis of control and *Naa30A* deletion males. Scale bar, 500 µm. Bottom panel: Differential interference contrast images of the apical tip of slightly squashed testis of control and Naa30A deletion males. Scale bar, 20 µm. **(d)** *Naa30A* deletion females also show an accelerated age-dependent loss of motility. Climbing ability was used as a measure of mobility and represents the percentage of females that were able to climb 8 cm after 10 and 30 s. Control females and *Naa30A* deletion homozygous females, at the ages of 1-4 days and 4-7 days after pupae eclosion were used. Results were obtained with females collected from two independent biological replicas. Each dot represents the average climbing ability of a group of 10 females after 10 technical replicates, mean ± SEM is also indicated. Statistical significance was assessed using the one-way ANOVA with Šídák's multiple comparisons test. **(e)** Loss of *Naa30A* significantly reduces adult female longevity. Survival curves of control females and *Naa30A* deletion homozygous females. Statistical significance was assessed using log-rank (Mantel-Cox) test, n represents the number of females used each survival curve. **(f)** Sagittal sections of adult indirect flight muscles (IFMs) stained for F-actin (Phalloidin, red) and nuclei (Mef2, green). Naa30A deletion resulted in a reduction of the number of IFMs, and this phenotype was rescued by a genomic construct containing Naa30A and by UbcE2M overexpression. Scale bar, 100 µm. * marks individual dorsal longitudinal IFM. **(g-h)** *Naa30A* deletion males have abnormal attachment of their IFM muscles. **(g)** Sagittal sections of adult indirect flight muscles (IFMs) stained for F-actin (Phalloidin, red) and nuclei (Mef2, green) representing the IFM attachment defects observed in *Naa30A* deletion males. The insets show magnifications of the outlined regions displayed in the left. Scale bar, 50 µm. The genotype of the *Naa30A* deletion males used is y^{1}, w^{67c23}, $Naa30A^{\Delta 74}$/Y; +/+; Mhc-Gal4/+. **(h)** Quantification of *Naa30A* deletion males presenting normal and abnormal IFM attachment 4-7 and 7-10 days after pupae eclosion. Frequency of abnormal attachment of IFM muscles in 4-7 days old males: 6.7% in *Naa30A* deletion males and 0% in *Naa30A* deletion males with *Naa30A* genomic rescue 1. Frequency of abnormal attachment of IFM muscles in 7-10 days old males: 43.8% in *Naa30A* deletion males and 0%

in *Naa30A* deletion males with *Naa30A* genomic rescue 1. The results represent the percentage of males displaying abnormal attachment, which were obtained from two independent crosses and *n* represents the total number of males analyzed. **(i)** Abnormal IFM attachment in *Naa30A* deletion males flight muscles are rescued by UbcE2M overexpression in muscles. Quantification of the abnormal IFM attachment phenotype in *Naa30A* deletion males and *Naa30A* deletion males overexpressing UbcE2M in muscle, at 7-10 days after pupae eclosion. Frequency of abnormal attachment of IFM muscles: 47.4% in *Naa30A* deletion males and 13.3% in *Naa30A* deletion mutants with muscle specific overexpression of UbcE2M. The results represent the percentage of males displaying abnormal attachment, which were obtained from two independent crosses and *n* represents the total number of males analyzed. Genotypes of the males used are: **(a-c)** control males (y^1/Y), *Naa30A* deletion males (y^1, Naa30A$^{\Delta 74}$/Y) and *Naa30A* deletion males with the *Naa30A* genomic rescue 1 (y^1, Naa30A$^{\Delta 74}$/Y; +/+; gNaa30A-myc/+); **(d-e)** control females (y^1, w^{67c23}/ y^1, w^{67c23}) and *Naa30A* deletion homozygous females (y^1, w^{67c23}, Naa30A$^{\Delta 74}$/ y^1, w^{67c23}, Naa30A$^{\Delta 74}$); **(f)** control males (y^1, w^{67c23}, Naa30A$^{\Delta 74}$/Y; +/+; Mhc-Gal4/+ and y^1, w^{67c23}, Naa30A$^{\Delta 74}$/Y; +/+; UAS-UbcE2M-HA/+) males overexpressing UbcE2M in muscle (y^1, w^{67c23}, Naa30A$^{\Delta 74}$; +/+; Mhc-Gal4/ UAS-UbcE2M-HA) males with the *Naa30A* genomic rescue 1 (y^1, w^{67c23}, Naa30A$^{\Delta 74}$/Y; +/+; gNaa30A-myc/+); **(h)** *Naa30A* deletion males (y^1, Naa30A$^{\Delta 74}$/Y) and *Naa30A* deletion males with the *Naa30A* genomic rescue 1 (y^1, Naa30A$^{\Delta 74}$/Y; +/+; gNaa30A-myc/+); **(i)** *Naa30A* deletion males carrying the UbcE2M construct (y^1, w^{67c23}, Naa30A$^{\Delta 74}$; +/+; UAS-UbcE2M-HA/+) *Naa30A* deletion males overexpressing UbcE2M in muscle (y^1, w^{67c23}, Naa30A$^{\Delta 74}$; +/+; Mhc-Gal4/ UAS-UbcE2M-HA). Source data are provided as a Source Data file.

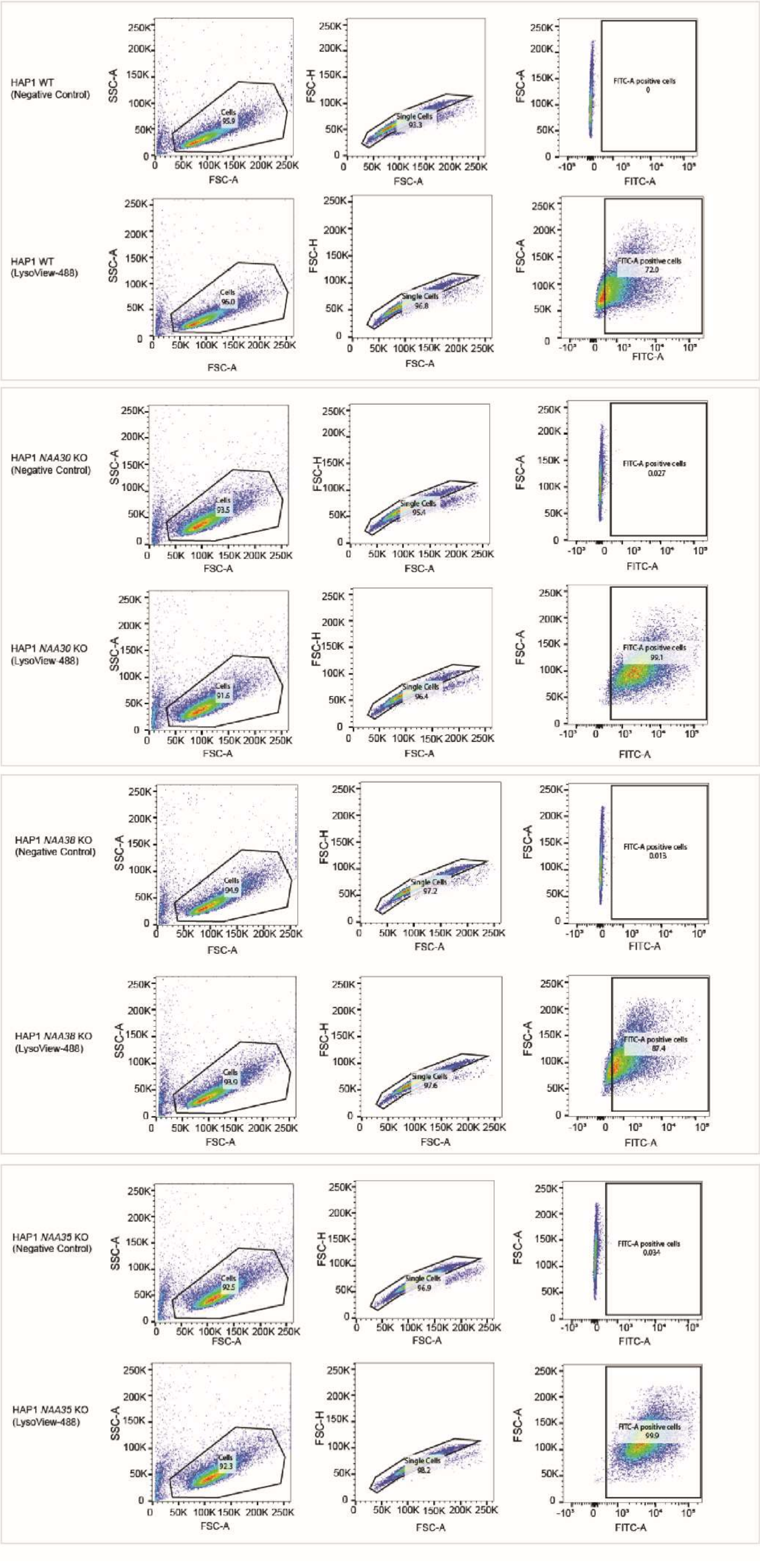
HAP1 WT
(Negative Control)
HAP1 WT
(LysoView-488)
HAP1 NAA30 KO
(Negative Control)
HAP1 NAA30 KO
(LysoView-488)
HAP1 NAA38 KO
(Negative Control)
HAP1 NAA38 KO
(LysoView-488)
HAP1 NAA35 KO
(Negative Control)
HAP1 NAA35 KO
(LysoView-488)
SSC-A
FSC-A
FSC-H
FITC-A
Cells
Single Cells
FITC-A positive cells

Supplementary Fig. 14: Gating strategies for flow cytometry experiments where lysosomes were labeled with LysoView 488.

The gating strategy included the following steps: (1) side scatter area (SSC-A) vs forward scatter area (FSC-A) were used to separate live cells from debris, (2) forward scatter height (FSC-H) vs forward scatter area (FSC-A) was used to separate single cells from aggregates, and (3) forward scatter area (FSC-A) vs LysoView 488 stain (FITC-A, fluorescence at 530/30 nm) were used to gate for live stained cells. For lysosome quantification, positively stained cells were quantified relative to unstained controls following the same gating strategy.

Supplementary Methods

Holographic live cell imaging

HAP1 cells were seeded in a 24-well plate (16,000 cells/well) in 2.5 mL filtrated IMDM medium and incubated for 20-30 min at RT to ensure cell attachment. Cells were monitored for 48 h in a HoloMonitor M4 live cell imaging system (Phase Holographic Imaging PHI AB, Lund, Sweden) acquiring holographic images every 10 min at 20× magnification. Each cell line was imaged from three different wells with one randomly chosen field of view per well (n = 3). Images were analyzed using HoloMonitor App Suite version 3.5.0.214. Single cells were automatically identified using auto minimum error with background threshold set to 120-140 and minimum object size set to 15-25. For analysis, images were manually examined to ensure correct cell number, size and morphology was identified, and out-of-focus images were removed. Cell proliferation analysis is based on cell count from images taken every 4th h in the timeframe 0-42 h. Growth curve shows mean ± SEM of three independent experiments. Cell proliferation was quantified by comparing relative increase in cell numbers at 42 h. Data are shown as mean ± SEM of three independent experiments pooled together and are expressed relative to WT (n=9). Significance was determined using one-way ANOVA with Dunnett's correction.

Saccharomyces cerevisiae strains

The *S. cerevisiae* haploid strain BY4741 Arl3-GFP was obtained from Invitrogen. Deletion of *NAA30/MAK3/YPR051W* was performed by homologous recombination and verified by PCR

as previously described[3]. Arl3-GFP *naa30Δ* strain was transformed with pBEVY-U-HA-DmNAA30, and both the Arl3-GFP WT and *naa30Δ* strains were transformed with empty pBEVY-U vector[4] to enable the same growth conditions. Yeast strains were grown in SD-Ura medium (Sunrise Science Products) at 30°C. For microscopy analysis, the cells were diluted from overnight cultures and grown to exponential growth phase OD_{600} 0.8-1.2. Cell preparation and live imaging were performed as previously described[3,5]. Images were taken using a Leica DMI6000 B widefield microscope equipped with a Leica DC500 camera and a 100 × 1.4 NA oil objective in addition to a 2 × magnification lens.

***Drosophila* immunoprecipitation**

For protein extracts, embryos with 0-4 hours expressing Naa30A-myc (CG11412) were dechorionated with 50% of bleach. Protein extraction was performed through homogenization of embryos in NB buffer (150 mM NaCl, 50 mM Tris–HCl pH 7.5, 2 mM EDTA, 0.1% NP-40, 1 mM DTT, 10 mM NaF, and EDTA-free protease inhibitor cocktail, Roche, Germany), and centrifugation at 20,000 × *g* for 3 min. Supernatant was collected and centrifuged twice. Total protein concentration was determined using Bio-Rad protein assay (Bio-Rad, Hercules, CA, USA) that is based on the Bradford dye-binding method.

For co-immunoprecipitation, proteins extracts (1.5 mg) of embryos expressing CG11412-myc were incubated with 1 µg of c-Myc antibody (Santa Cruz, sc-40, clone 9E10) for 1 h at 4°C. Subsequently, 0.9 mg of Dynabeads Protein G (Invitrogen) was added and incubated for 1 h at 4°C. After washing the beads 3 times with NB buffer, protein elution was performed with 100 µL of 100 mM Glycine pH 3.0 for 1 min and stopped with 10 µL of 1 M Tris Base pH 10.8. Proteins of the eluate were precipitated with 5 volumes of acetone at −20°C.

To determine the efficiency of Naa30A-myc immunoprecipitation, total protein extracts and eluted proteins were boiled for 5 min in SDS-PAGE sample buffer. Samples were centrifuged and loaded on a 10% SDS-gel for electrophoresis and transferred to a nitrocellulose membrane. Immunoblot analysis was performed according to standard protocols using the anti-myc antibody (Covance, PRB-150C-200, 1:2000).

Proteomic analysis of *Drosophila* proteins

Mass Spectrometry analysis was performed at the Mass Spectrometry Laboratory, Institute of Biochemistry and Biophysics, Polish Academy of Sciences, Warsaw, Poland. Briefly, peptides mixtures were analyzed by LC-MS/MS (liquid chromatography coupled to tandem mass spectrometry) using Nano-Acquity LC system (Waters, Milford, MA, USA) and Orbitrap Velos mass spectrometer (Thermo Electron Corp., San Jose, CA, USA). Prior to analysis, proteins were subjected to standard "in-solution digestion" procedure, during which proteins were reduced with 100 mM DTT (for 30 min at 56°C), alkylated with 0.5 M iodoacetamide for 45 min in the dark at room temperature, and digested overnight with trypsin (Promega, Cat # V5111). The peptide mixture was applied to a RP-18 pre-column (nanoACQUITY Symmetry C18, Waters, 186003514) using water containing 0.1% TFA as mobile phase, then transferred to nano-HPLC RP-18 column (nanoACQUITY BEH C18, Waters, 186003545) using an acetonitrile gradient (0%-35% ACN in 180 min) in the presence of 0.05% formic acid with a flow rate of 250 nL/min. The column outlet was directly coupled to the ion source of the spectrometer, operating in the regime of data dependent MS to MS/MS switch. A blank run ensuring no cross contamination from previous samples preceded each analysis.

Raw data were processed by Mascot Distiller followed by Mascot Search (Matrix Science) against the Flybase database (FB2018_05). Search parameters for precursor and product ions mass tolerance were 15 ppm and 0.4 Da, respectively, enzyme specificity: trypsin, missed cleavage sites allowed: 0, fixed modification of cysteine by carbamidomethylation, and variable modification of methionine oxidation. Peptides with Mascot Score exceeding the threshold value corresponding to <5% False Positive Rate, calculated by Mascot procedure, and with the Mascot score above 30 were positively identified. Human orthologs were determined using DSRC Integrative Ortholog Prediction Tool (DIOPT)[6]. Only scores above two were considered the best matches when there was more than one match per input.

Scoring of the held-out wing phenotype.

Live flies with 7-10 days after pupae eclosion were used for scoring the held-out wing phenotypes. Anesthesia was kept brief to minimize abnormal wing positions caused by the procedure itself, even in wild-type flies. Flies were briefly anesthetized using CO2 and photographed upon recovery. The intensity of the held-out wing phenotype was classified into

three classes for scoring: class 1 representing normal wings, class 2 indicating weak held-out wing phenotype, and class 3 representing strong held-out wing phenotype. Examples of the different classes can be observed in **Supplementary Fig. 12b**.

Mitochondrial morphology in *Drosophila*

Mitochondria morphology of the flight muscles were analyzed. To this purpose thoraxes from control and *Naa30A* deletion males expressing the mitochondrial marker mitoGFP by da-GAL4 driver were dissected and mitochondrial morphology imaged on a Zeiss LSM710 confocal microscope.

Assessment of ATP levels

ATP levels were determined as described in[7]. Briefly, for each genotype groups of 5 males were collected in Eppendorf tubes and then homogenized in PBS using a potter homogenizer. An aliquot was incubated on ice with 12% perchloric acid for 1 min and then neutralized with a solution of 3 M K_2CO_3 and 2 M Tris. After centrifugation at 13,000 rpm for 1 min, ATP content was measured at 25˚C using the ATP Bioluminescence Assay kit (Sigma-Aldrich), according to manufacturer's instruction. The luminescence was calculated as an average of three technical replicates and normalized to the total protein levels quantified using the Bradford's method (Bio-Rad). Results are from the two or three biological replicas and are expressed as a percentage of the normalized luminescence of the control.

Obtaining homozygous *Naa30A* females

To generate homozygous *Naa30A* females, we crossed heterozygous *Naa30A* deletion females (y^1, w^{67c23}, Naa30A$^{\Delta 74}$/FM0) with males carrying the *Naa30A* genomic construct in the 3rd chromosome. The *Naa30A* genomic construct has an associated mini-white eye color marker. This allowed us to produce *Naa30A* deletion males carrying the genomic rescue (y1, w^{67c23}, Naa30A$^{\Delta 74}$/Y; +/+; gNaa30A-myc/+), which were fertile. Subsequently, we crossed these males with heterozygous *Naa30A* deletion females. The resulting offspring were sorted based on eye

color to identify and select homozygous *Naa30A* deletion females that lacked the genomic rescue.

Supplementary References

1. Grunwald, S. et al. Divergent architecture of the heterotrimeric NatC complex explains N-terminal acetylation of cognate substrates. *Nature Communications* **11**(2020).

2. Huang, D.T. et al. Structural basis for recruitment of Ubc12 by an E2 binding domain in NEDD8's E1. *Mol Cell* **17**, 341-50 (2005).

3. Osberg, C., Aksnes, H., Ninzima, S., Marie, M. & Arnesen, T. Microscopy-based Saccharomyces cerevisiae complementation model reveals functional conservation and redundancy of N-terminal acetyltransferases. *Sci Rep* **6**, 31627 (2016).

4. Miller, C.A., 3rd, Martinat, M.A. & Hyman, L.E. Assessment of aryl hydrocarbon receptor complex interactions using pBEVY plasmids: expressionvectors with bi-directional promoters for use in Saccharomyces cerevisiae. *Nucleic Acids Res* **26**, 3577-83 (1998).

5. Aksnes, H., Osberg, C. & Arnesen, T. N-terminal acetylation by NatC is not a general determinant for substrate subcellular localization in Saccharomyces cerevisiae. *PLoS One* **8**, e61012 (2013).

6. Hu, Y.H. et al. An integrative approach to ortholog prediction for disease-focused and other functional studies. *Bmc Bioinformatics* **12**(2011).

7. Lovero, D. et al. Characterization of Drosophila ATPsynC mutants as a new model of mitochondrial ATP synthase disorders. *PLoS One* **13**, e0201811 (2018).
