# Supplementary material for: N-terminal acetylation shields proteins from degradation and promotes age-dependent motility and longevity
Source: Nat Commun. 2023 Oct 27;14:6774. doi: 10.1038/s41467-023-42342-y (PMC10611716; doi:10.1038/s41467-023-42342-y)
Supplement: Supplementary file 2 — Reporting Summary [file 41467_2023_42342_MOESM2_ESM.pdf]

nature portfolio

Corresponding author(s): Sylvia Varland, Rui D. Silva, Rui G. Martinho, Thomas Arnesen

Last updated by author(s): Sep 27, 2023

Reporting Summary

Nature Portfolio wishes to improve the reproducibility of the work that we publish. This form provides structure for consistency and transparency in reporting. For further information on Nature Portfolio policies, see our Editorial Policies and the Editorial Policy Checklist.

Statistics

For all statistical analyses, confirm that the following items are present in the figure legend, table legend, main text, or Methods section.

n/a	Confirmed	
☐	☒	The exact sample size (*n*) for each experimental group/condition, given as a discrete number and unit of measurement
☐	☒	A statement on whether measurements were taken from distinct samples or whether the same sample was measured repeatedly
☐	☒	The statistical test(s) used AND whether they are one- or two-sided<br>*Only common tests should be described solely by name; describe more complex techniques in the Methods section.*
☒	☐	A description of all covariates tested
☒	☐	A description of any assumptions or corrections, such as tests of normality and adjustment for multiple comparisons
☐	☒	A full description of the statistical parameters including central tendency (e.g. means) or other basic estimates (e.g. regression coefficient) AND variation (e.g. standard deviation) or associated estimates of uncertainty (e.g. confidence intervals)
☐	☒	For null hypothesis testing, the test statistic (e.g. *F*, *t*, *r*) with confidence intervals, effect sizes, degrees of freedom and *P* value noted<br>*Give P values as exact values whenever suitable.*
☒	☐	For Bayesian analysis, information on the choice of priors and Markov chain Monte Carlo settings
☒	☐	For hierarchical and complex designs, identification of the appropriate level for tests and full reporting of outcomes
☐	☒	Estimates of effect sizes (e.g. Cohen's *d*, Pearson's *r*), indicating how they were calculated

Our web collection on statistics for biologists contains articles on many of the points above.

Software and code

Policy information about availability of computer code

Data collection	Andor Dragonfly 500 confocal microscopy, Oxford instruments<br>Axiovert 200M widefield fluorescence microscope, Carl Zeiss AG<br>ChemiDoc XRS+ imaging system, Bio-Rad<br>Gel Doc EZ imaging system, Bio-Rad<br>HoloMonitor M4 system, Phase Holographic Imaging PHI AB<br>Infinite 200Pro plate reader, Tecan<br>LSRFortessa Cell Analyzer, BD Bioscience<br>Lunatic microfluidic device, Unchained Labs<br>Orbitrap Fusion Lumos mass spectrometer, Thermo Scientific<br>Orbitrap Velos mass spectrometer, Thermo<br>Sony SH800 cell sorter, Sony Biotechnology<br>Trans-Blot Turbo Transfer System, Bio-Rad<br>Tri-Carb 2900TR Liquid Scintillation Analyzer, PerkinElmer
Data analysis	Adobe Illustrator 2020, Adobe Inc (https://www.adobe.com), RRID:SCR_010279<br>Adobe Photoshop CS5, Adobe Inc (https://www.adobe.com), RRID:SCR_014199<br>Bowtie v0.12.8 (https://bowtie-bio.sourceforge.net/), RRID:SCR_005476<br>CHOPCHOP v3 (https://chopchop.cbu.uib.no/), RRID:SCR_015723<br>DSRC Integrative Ortholog Prediction Tool (DIOPT, https://www.flyrnai.org/cgi-bin/DRSC_orthologs.pl)<br>FACSDiva version 9.0.1, BD Biosciences (https://www.bdbiosciences.com), RRID:SCR_001456<br>FlowJo version 10.8.1, BD Life Sciences (https://www.flowjo.com/), RRID:SCR_008520

Flybase database (https://flybase.org/), RRID:SCR_006549
GraphPad Prism version 9.3.1, GraphPad Software (https://www.graphpad.com/), RRID:SCR_002798
HoloMonitor App Suite version 3.5.0.214, PHI AB (https://phiab.com/holomonitor/), RRID:SCR_019231
Image Lab version 6.0.1, Bio-Rad (https://www.bio-rad.com), RRID:SCR_014210
ImageJ/Fiji version 2.1.0/1.53c, (https://imagej.net/), RRID:SCR_002285
IMARIS version 9.7.2, Oxford instruments (https://imaris.oxinst.com/), RRID:SCR_00730
Mascot Daemon interface version 2.6.2, Matrix Science (https://www.matrixscience.com/), RRID:SCR_000307
Mascot Distiller version 2.7.1.0, Matrix Science (https://www.matrixscience.com/), RRID:SCR_000307
MaxQuant versions 2.0.1.0 and 1.6.17.0, (https://www.maxquant.org/), RRID:SCR_014485
Microsoft Excel, Microsoft (https://www.microsoft.com/), RRID:SCR_016137
Perseus version 1.6.15.0 (https://maxquant.org/perseus/), RRID:SCR_015753
SwissProt database (https://www.expasy.org/resources/uniprotkb-swiss-prot), RRID:SCR_021164
Tecan i-control software version 2.0.10.0, Tecan (https://www.tecan.com/), RRID:SCR_016771

For manuscripts utilizing custom algorithms or software that are central to the research but not yet described in published literature, software must be made available to editors and reviewers. We strongly encourage code deposition in a community repository (e.g. GitHub). See the Nature Portfolio guidelines for submitting code & software for further information.

Data

Policy information about availability of data

All manuscripts must include a data availability statement. This statement should provide the following information, where applicable:

- Accession codes, unique identifiers, or web links for publicly available datasets
- A description of any restrictions on data availability
- For clinical datasets or third party data, please ensure that the statement adheres to our policy

The authors confirm that data supporting the findings presented in this study are available in the article and its supplemental data files.

CRISPR screen sequencing data have been deposited in NCBI's Gene Expression Omnibus and are accessible through GEO Series accession number GSE221447 (https://www.ncbi.nlm.nih.gov/geo/query/acc.cgi?acc=GSE221447).

Mass spectrometry proteomics data have been deposited to the ProteomeXchange Consortium (http://proteomecentral.proteomexchange.org) via the PRIDE partner repository (Perez-Riverol et al., 2019) with the following dataset identifiers:
N-terminal acetylome analysis of HAP1 WT and NatC KO cells (PXD034992)
LFQ shotgun proteomic analysis of HAP1 WT and NatC KO cells (PXD034104)
TMT analysis of HAP1 WT siCtrl, WT siUBR4, NAA30-KO siCtrl, and NAA30-KO siUBR4 (PXD034410)

Human research participants

Policy information about studies involving human research participants and Sex and Gender in Research.

Reporting on sex and gender	There were no human research participants in this study
Population characteristics	There were no human research participants in this study
Recruitment	There were no human research participants in this study
Ethics oversight	There were no human research participants in this study

Note that full information on the approval of the study protocol must also be provided in the manuscript.

Field-specific reporting

Please select the one below that is the best fit for your research. If you are not sure, read the appropriate sections before making your selection.

☒ Life sciences ☐ Behavioural & social sciences ☐ Ecological, evolutionary & environmental sciences

For a reference copy of the document with all sections, see nature.com/documents/nr-reporting-summary-flat.pdf

Life sciences study design

All studies must disclose on these points even when the disclosure is negative.

Sample size	No statistical methods were used to pre-determine sample size, but our sample size is according to conventions in the field. For small-scale experiments, the number of replicates exceeds at least 3 biologically independent experiments and/or at least 3 technical replicates, as indicated each figure legend and/or in the Methods.

Data exclusions	All technically sound data were included.
Replication	Experiments were replicated for the indicated number of times, at least three times for cell culture experiments. We present no experimental results that were not reproducible.
Randomization	No randomization was performed in this study, due to small sample size.
Blinding	For mass spectrometry analysis, blinding was not done since the small sample groups have to be defined during data analysis. Determination of significance was solely based on automated procedures. For flow cytometry, microscopy, and immunoblotting blinding was not done since samples were prepared and analyzed by the same researcher in small batches.

Reporting for specific materials, systems and methods

We require information from authors about some types of materials, experimental systems and methods used in many studies. Here, indicate whether each material, system or method listed is relevant to your study. If you are not sure if a list item applies to your research, read the appropriate section before selecting a response.

Materials & experimental systems

n/a	Involved in the study	
☐	☒	Antibodies
☐	☒	Eukaryotic cell lines
☒	☐	Palaeontology and archaeology
☐	☒	Animals and other organisms
☒	☐	Clinical data
☒	☐	Dual use research of concern

Methods

n/a	Involved in the study	
☒	☐	ChIP-seq
☐	☒	Flow cytometry
☒	☐	MRI-based neuroimaging

Antibodies

Antibodies used

All antibodies used in this study are listed in Supplementary Data 8.
anti-ARFRP1 (1:1000, Sigma-Aldrich, HPA04702, Lot 000006356)
anti-BCL2 (1:500, Proteintech, 12789 1 AP, Lot 00071452)
anti-CAPNS1 (1:1000, Thermo Scientific, PA5-82266, Lot A105125)
anti-c-Myc (1:2000, Covance, PRB-150C-200)
anti-c-Myc (Santa Cruz, sc-40, Lot A2315)
anti-COX IV (1:200, Cell Signaling, 4850, Lot 10)
anti-CUL1 (1:1000, Cell Signaling, 4995, Lot 2)
anti-CUL2 (1:1000, Abcam, ab166917, Lot GR37222222.5)
anti-CUL3 (1:1000, Cell Signaling, 2759, Lot 2)
anti-CUL4A (1:1000, Cell Signaling, 2699, Lot 4)
anti-CUL4B (1:1000, Proteintech, 12916-1-AP, Lot 00019694)
anti-CUL5 (1:1000, Abcam, ab264284, GR3349206.9)
anti-DIS3 (1:1000, Thermo Scientific, PA5-78427, Lot WE3267991C)
anti-EEA1 (1:1000, Santa Cruz, sc-33585, Lot c1210)
anti-FLAG (1:3000, Sigma, clone M2, F3165, Lot SLCJ3741)
anti-GAPDH (1:10000, Santa Cruz, sc-47724, Lot g2920)
anti-GFP (1:5000, Roche, clones 7.1 and 13, 11814460001, Lot 4785906)
anti-HK1 (1:2000, Thermo Scientific, MA5-14789, Lot WC3724751)
anti-IST1 (1:1000, GeneTex, GTX101972, Lot 39596)
anti-KCMF1 (1:1000, Sigma-Aldrich, HPA030383, Lot 000027971)
anti-LC3B (1:1000, Thermo Scientific, PA1-46286, Lot WG3311175)
anti-Mef2 (1:200, Gift from Dr. Eileen Furlong)
anti-NAA30 (1:1000, Sigma-Aldrich, HPA057824, Lot R80962)
anti-p62/SQSTM1 (1:200-1:1000, Santa Cruz, sc-28359, Lot k1319)
anti-RBX1 (1:1000, Abcam, ab221548, Lot GR3480235-3)
anti-RBX2/RNF7 (1:1000, Abcam, ab181986, Lot GR149431-14)
anti-RGS10 (1:1000, Abcam, ab154172, Lot GR117258-3)
anti-RSPRY1 (1:1000, Thermo Scientific, PA5-32048, Lot WC3231244C)
anti-SLC10A7 (1:1000, Sigma-Aldrich, SAB2102163, Lot QC14024)
anti-UBE2A/UBE2B (1:2000, Abcam, ab31917, Lot GR78358-1)
anti-UBE2F (1:1000, Abcam, ab185234, Lot GR160302-11)
anti-UBE2M (1:1000, Abcam, ab109507, Lot GR90673-10)
anti-Ubiquitin (mono and poly) (1:250, Enzo Life Sciences, BML-PW8810, Lot 07281715)
anti-UBR1 (1:1000, Bethyl Laboratories, A302-988A-M, Lot 1)
anti-UBR2 (1:2000, Abcam, ab217069, GR3174472-22)
anti-UBR4 (1:1000, Abcam, ab86738, GR214363-47)
anti-UBR5 (1:1000, Cell Signaling, 65344, Lot 1)
anti-V5 (1:5000-20,000, Invitrogen, R960CUS, Lot 2382869)
anti-vinculin (1:10000, Abcam, ab129009, Lot GR3395452)

anti-Rabbit HRP-linked (1:1000-1:10000, Cytiva, NA934, Lot 17187089)
anti-Mouse HRP-linked (1:3000-1:20000, Cytiva, NA931, Lot 17162269)
Alexa Fluor 488 goat anti-mouse (1:1000, Thermo Fisher, A-11029, 56649A)
Alexa Fluor 488 goat anti-mouse (1:100, Jackson Immuno Research, 115-546-003, Lot 147924)
Alexa Fluor 555 donkey anti-rabbit (1:200, Thermo Fisher, A31572, Lot 2482963)
Alexa Fluor 594 goat anti-rabbit (1:100, Jackson Immuno Research, 111.586-003, Lot 138323)

Validation

All antibodies, except for anti-Mef2, are commercially available and tested by the manufacturers. In addition, anti-NAA30 was validated by immunoblotting using HAP1 NAA30 knockout cells.

Eukaryotic cell lines

Policy information about cell lines and Sex and Gender in Research

Cell line source(s)

The near haploid human HAP1 WT cells (clone C631; sex: male; RRID:CVCL_Y019) and the HAP1 gene-KO cell lines NAA30-KO (HZGHC006637c010) and NAA35-KO (HZGHC006636c011) were obtained from Horizon Genomics GmbH (Vienna, Austria). HAP1 NAA38-KO (KO2 in original ref) and an unmodified HAP1 control cell line that underwent CRISPR treatment (herein referred to as WT2) were a generous gift from Dr. Scott Dixon, Stanford University (Cao et al., 2019). The human cervix carcinoma cell line HeLa (clone CCL-2; sex: female; RRID:CVCL_0030) and human breast cancer cell line MDA-MB-231 (sex: female; RRID:CVCL_0062) were obtained from ATCC.

Authentication

Gene disruption in the HAP1 KO cell lines were confirmed by Sanger sequencing of PCR products of the edited genomic region. NAA30 disuption in the MDA-MB-231 cell line were confirmed by Sanger sequencing and immunoblotting. No further authentication was performed.

Mycoplasma contamination

Cells were routinely tested for mycoplasma using the MycoAlert Detection Kit (Lonza) or by NucBlue DAPI staining (Invitrogen). All cell lines tested negative for mycoplasma contamination.

Commonly misidentified lines
(See ICLAC register)

The HeLa cell line has not been authenticated and was only used for transient transfection of NAA30-V5 for immunoprecipitation and acetylation assays and UBR4-V5 for peptide pulldown assays.

Animals and other research organisms

Policy information about studies involving animals; ARRIVE guidelines recommended for reporting animal research, and Sex and Gender in Research

Laboratory animals

Drosophila melanogaster and Saccharomyces cerevisiae. All strains and stocks are described in Supplementary Data 8. For each figure, the used Drosophila stocks, along with their corresponding ages, are always indicated in the respective legends and the Materials and Methods section.
Drosophila strains used:
Oregon R, Lab collection
y1, Bloomington Drosophila Stock Center, RRID:BDSC_169
y1, Naa30AΔ74/FM0, This study
w, y1, Naa30AΔ74/FM0, This study
w, y1, Naa30AΔ74/FM0/DP (1;Y) y+, This study
w, y1, Naa30AΔ74/FM0;;UAS-UbcE2M/TM3,sb, This study
w;; Naa30A-genomic-8myc, This study
w; Naa30A-genomic-8myc, This study
w;; Da-Gal4, Wodarz et al., 1995
y1 P{EPgy2}Naa30AEY10202 w67c23, Bloomington Drosophila Stock Center, RRID:BDSC_16976
w;; Mhc-Gal4, Bloomington Drosophila Stock Center, RRID:BDSC_55133
w;; UAS-UcbE2M3xH, Zurich ORFeome Project (FlyOrf), Cat # F003044

Wild animals

The study did not involve wild animals.

Reporting on sex

All Drosophila strains are described in Supplementary Data 8. Male/female status is indicated in all experiments in the article. Some phenotype experiments have been performed with both sexes (indicated in the article).

Field-collected samples

The study did not involve field-collected samples.

Ethics oversight

No ethical approval is required for Drosophila melanogaster studies.

Note that full information on the approval of the study protocol must also be provided in the manuscript.

Flow Cytometry

Plots

Confirm that:

☒ The axis labels state the marker and fluorochrome used (e.g. CD4-FITC).

☒ The axis scales are clearly visible. Include numbers along axes only for bottom left plot of group (a 'group' is an analysis of identical markers).

☒ All plots are contour plots with outliers or pseudocolor plots.

☒ A numerical value for number of cells or percentage (with statistics) is provided.

Methodology

Sample preparation	HAP1 cells were seeded in 6-well plates with a density of 150,000 cells/well or 60,000 cells/well for siRNA treatment and incubated for 24 h or 96 h, respectively. For estimation of cell granularity, cells were detached using TrypLE Express (Gibco), washed with PBS, and resuspended in FACS buffer (PBS with 5% FBS). For lysosomal analysis, cells were stained with LysoView 488 (Biotium) for 1 h at 37°C according to the manufacturer's protocol and processed as above. Cells were analyzed using BD LSR Fortessa flow cytometer.<br>MDA-MB-231 cells were seeded in 6 cm dishes, to reach 80% confluency the next day. Then the cells were transfected with the CRISPR-plasmid (coding for guideRNA, Cas9 and EGFP). The next day (24 h post transfection), cells were detached using trypsin, washed with PBS and resuspended in cell medium. Single fluorescent cells were sorted into a 96-well plate prepared with 200 uL conditioned medium, using SONY SH800 flow cytometer.
Instrument	Cell analysis: LSR Fortessa flow cytometer (BD Bioscience)<br>Cell sorting: SH800 cell sorter (SONY)
Software	FACSDiva software version 9.0.1 for data collection and FlowJo version 10.8.1 for data analysis (both from BD Bioscience).<br>For single-cell sorting, SONY SH800 software was used.
Cell population abundance	Due to the use of commercial HAP1 and MDA-MB-231 cell lines, the abundance of the relevant cell population was 100%.
Gating strategy	Gating strategy was used for (a) estimating cell granularity, (b) estimating lysosome content by LysoView 488 staining (Biotium), and (c) sorting MDA-MB-231 cells transfected with various pSpCas9(BB)-2A-GFP plasmids. In all cases the following steps were taken: (1) side scatter area (SSC-A) vs forward scatter area (FSC-A) were used to separate live cells from cell debris and dead cells, and (2) forward scatter area (FSC-A) vs forward scatter height (FSC-H) was used to separate single cells from doublets or aggregates. For fluorescent cells, the gating strategy included using (3) forward scatter area (FSC-A) vs fluorescence at 530/30 nm (FITC). Fluorescent cells included (b) cells stained with LysoView 488 (see Supplementary Fig. 14) or (c) transfected with various CRISPR-Cas9-GFP plasmids. The fluorescent gating strategy included a negative control to ensure precise fluorescence-based cell selection, excluding negative control cells.

☒ Tick this box to confirm that a figure exemplifying the gating strategy is provided in the Supplementary Information.
