# Supplementary material for: N-terminal acetylation shields proteins from degradation and promotes age-dependent motility and longevity
Source: Nat Commun. 2023 Oct 27;14:6774. doi: 10.1038/s41467-023-42342-y (PMC10611716; doi:10.1038/s41467-023-42342-y)
Supplement: Supplementary file 3 — Description of Additional Supplementary Files [file 41467_2023_42342_MOESM3_ESM.pdf]

Description of Additional Supplementary Files

File name: Supplementary Data 1
Description: Genetic interactions scores (qGI) from genome-wide CRISPR knockout screens in HAP1 *NAA30*-KO, *NAA35*-KO, and *NAA38*-KO cells.

File name: Supplementary Data 2
Description: Gene ontology (GO) biological process (BP) enrichment analysis of negative and positive genetic interactions of *NAA35*, *NAA30*, and *NAA38*.

File name: Supplementary Data 3
Description: N-terminal acetylome analysis of HAP1 WT, *NAA30*-KO, *NAA35*-KO, and *NAA38*-KO cells.

File name: Supplementary Data 4
Description: Label-free quantitative (LFQ) shotgun proteomic analysis of HAP1 WT, *NAA30*-KO, *NAA35*-KO, and *NAA38*-KO cells.

File name: Supplementary Data 5
Description: Knockdown of UBR4 rescues *NAA30*-KO induced changes in protein abundance. Tandem mass tag (TMT) analysis of HAP1 WT and *NAA30*-KO cells treated with siCtrl or siUBR1/UBR2/UBR4 (triple knockdown).

File name: Supplementary Data 6
Description: List of proteins co-immunoprecipitated with Myc-tagged *Drosophila* Naa30A from embryonic protein extracts.

File name: Supplementary Data 7
Description: List of all primers and peptides used in this study.

File name: Supplementary Data 8
Description: Key Resource Table (Antibodies, cell lines, fly strains, yeast strains, plasmids, siRNA, etc.).

File name: Supplementary Movie 1
Description: *Drosophila Naa30A* deletion males show significantly slower motility (climbing).

File name: Supplementary Movie 2
Description: Moving sperm from control (y1/Y) males.

File name: Supplementary Movie 3
Description: Moving sperm from *Naa30A* deletion (y1, Naa30Δ74/Y) males.
