# Supplementary material for: N-terminal acetylation shields proteins from degradation and promotes age-dependent motility and longevity
Source: Nat Commun. 2023 Oct 27;14:6774. doi: 10.1038/s41467-023-42342-y (PMC10611716; doi:10.1038/s41467-023-42342-y)
Supplement: Supplementary file 10 — Supplementary Data 7 [file 41467_2023_42342_MOESM10_ESM.pdf]

Supplementary Data 7

Primers used in this study.

Primer name	Sequence (5'-3')	Usage
**Cell line knockout verification**		
NAA30-KO gDNA F	CAACGGATTGATTAGCCCCGAA	HAP1 *NAA30*-KO, quality control, PCR, sequencing
NAA30-KO gDNA R	GACATATCGTATCGTCCGATCCTC	HAP1 *NAA30*-KO, quality control, PCR
NAA35-KO gDNA F	CGACAATGATTCCCATAGACACTTG	HAP1 *NAA35*-KO, quality control, PCR, sequencing
NAA35-KO gDNA R	ATCTTACCTCTTCAAAAACAGCAGC	HAP1 *NAA35*-KO, quality control, PCR
NAA38-KO gDNA F	GCATCCCAGCTACACACAGA	HAP1 *NAA38*-KO, quality control, PCR, sequencing
NAA38-KO gDNA R	GTACGCTTCAGTGAGCCACA	HAP1 *NAA38*-KO, quality control, PCR, sequencing
**CRISPR/Cas9-mediated *NAA30* gene disruption in human MDA-MB-231 cell line**		
NAA30 sgRNA F	CACC**g**AGCGGCGGCGAGCGATCCCG	Underlined: flanks matching the BsbI restriction site. Bold: inserted nucleotide to increase RNA Pol III initiation. Capital: align with *NAA30* or non-targeting sequence
NAA30 sgRNA R	AAACCGGGATCGCTCGCCGCCGCT**c**	
Non-targeting sgRNA F	CACC**g**TATTACTGATATTGGTGGG	
Non-targeting sgRNA F	AAACCCCACCAATATCAGTAATA**c**	
hU6 sequencing F	CGATACAAGGCTGTTAGAGAG	Human U6 forward sequencing primer.
**Illumina sequencing**		
PCR1 F	GAGGGCCTATTTCCCATGATTC	CRISPR library prep, PCR1
PCR1 R	GTTGCGAAAAAGAACGTTCACGG	CRISPR library prep, PCR1
PCR2 pLCV2 U6 amplicon F	AATGATACGGCGACCACCGAGATCTACACnnnnnnnnACACTCTTTCCCTACACGACGCTCTTCCGATCTTTGTGGAAAGGACGAAACACCG	CRISPR library prep, PCR2<br><br>pLVC2: lentiCRISPR v2<br>nnnnnnnn: denotes i5 or i7 index<br>underlined: denotes annealing sequence
PCR2 pLCV2 U6 amplicon R	CAAGCAGAAGACGGCATACGAGATnnnnnnnnGTGACTGGAGTTCAGACGTGTGCTCTTCCGATCTACTTGCTATTTCTAGCTCTAAAAC	
PCR2 pLCV2 tracr amplicon F	AATGATACGGCGACCACCGAGATCTACACnnnnnnnnACACTCTTTCCCTACACGACGCTCTTCCGATCTACTTGCTATTTCTAGCTCTAAAAC	
PCR2 pLCV2 tracr amplicon R	CAAGCAGAAGACGGCATACGAGATnnnnnnnnGTGACTGGAGTTCAGACGTGTGCTCTTCCGATCTTTGTGGAAAGGACGAAACACCG	
**Plasmid cloning, mutagenesis, and sequencing**		
pCMV6-UBE2M I2G F	GATCGCCATGggCAAGCTGTTCTC	UBE2M mutagenesis
pCMV6-UBE2M I2A F	GATCGCCATGgcCAAGCTGTTCTC	UBE2M mutagenesis
pCMV6-UBE2M I2D F	GATCGCCATGgaCAAGCTGTTCTCGCTG	UBE2M mutagenesis
pCMV6-UBE2M I2E F	GATCGCCATGgagAAGCTGTTCTC	UBE2M mutagenesis
pCMV6-UBE2M I2L F	GATCGCCATGcTCAAGCTGTTCTCGCTG	UBE2M mutagenesis
pCMV6-UBE2M I2F F	GATCGCCATGtTCAAGCTGTTCTCGCTG	UBE2M mutagenesis
pCMV6-UBE2M I2Y F	GATCGCCATGtaCAAGCTGTTCTC	UBE2M mutagenesis
pCMV6-UBE2M I2P F	GATCGCCATGccCAAGCTGTTCTCGCTG	UBE2M mutagenesis
pCMV6-UBE2M Mut R	GCGGCGGCAGATCTCCTC	UBE2M mutagenesis

pCMV6-UBE2M del F	ACGCGTACGCGGCCGCTC	UBE2M deletion mutagenesis
pCMV6-UBE2M del R	GGCGATCGCGGCGGCAGATC	UBE2M deletion mutagenesis
NAA30 E321A F	GGTTGTTTTGGcAACCGAAATAAC	NAA30 E321A mutagenesis
NAA30 E321A R	TCATCACAGTCTCCCTCAAC	NAA30 E321A mutagenesis
T7 promoter F	TAATACGACTCACTATAGGG	Plasmid sequencing
CMV promoter F	GAGGTCTATATAAGCAGAGC	Plasmid sequencing
BGH R	TAGAAGGCACAGTCGAGG	Plasmid sequencing
M13 R	CAGGAAACAGCTATGAC	Plasmid sequencing
ADH1 promoter F	CCTCGTCATTGTTCTCGTTCC	Plasmid sequencing (yeast)
***Drosophila* knockout confirmation**		
CG11412/Naa30A F	CAAGGAAAGTGGAGGAAGTGC	Confirming *Naa30A* deletion
CG11412/Naa30A R	GGTATGTATCCCTCGCCAATG	Confirming *Naa30A* deletion

Peptides used in this study.

Peptide name	Sequence	Derived from	UniProt KB	Supplier	Usage
MIKLFSL (UBE2M)	MIKLFSL RWGRPVGRRRRPVRVYP	UBE2M	P61081	Innovagen AB	Acetylation assay
MYTLLSG (ARFRP1)	MYTLLSG RWGRPVGRRRRPVRVYP	ARFRP1	Q13795		
MLPSTSV (UNC50)	MLPSTSV RWGRPVGRRRRPVRVYP	UNC50	Q53HI1		
DDDIAAL (β-actin)	DDDIAAL RWGRPVGRRRRPVRVYP	β-actin	P60709	Biogenes	
Ac-MI-UBE2M-biotin	Ac-MIKLFSLKQQKK-biotin	UBE2M	P61081	Innovagen AB	Peptide pulldown
MI-UBE2M-biotin	MIKLFSLKQQKK-biotin	UBE2M	P61081		
ML-UBE2M-biotin	MLKLFSLKQQKK-biotin	UBE2M	P61081		
MY-UBE2M-biotin	MYKLFSLKQQKK-biotin	UBE2M	P61081		
MF-UBE2M-biotin	MFKLFSLKQQKK-biotin	UBE2M	P61081		
A-UBE2M-biotin	AIKLFSLKQQKK-biotin	UBE2M	P61081		
ME-UBE2M-biotin	MEKLFSLKQQKK-biotin	UBE2M	P61081		
R-UBE2M-biotin	RIKLFSLKQQKK-biotin	UBE2M	P61081		
F-UBE2M-biotin	FIKLFSLKQQKK-biotin	UBE2M	P61081		
R-nsP4-biotin	RIFSTIEGRTYK-biotin	Sindbis virus nsP4	-		
F-nsP4-biotin	FIFSTIEGRTYK-biotin	Sindbis virus nsP4	-		
G-nsP4-biotin	GIFSTIEGRTYK-biotin	Sindbis virus nsP4	-		
