# Supplementary material for: N-terminal acetylation shields proteins from degradation and promotes age-dependent motility and longevity
Source: Nat Commun. 2023 Oct 27;14:6774. doi: 10.1038/s41467-023-42342-y (PMC10611716; doi:10.1038/s41467-023-42342-y)
Supplement: Supplementary file 11 — Supplementary Data 8 [file 41467_2023_42342_MOESM11_ESM.pdf]

Key Resource Table

REAGENT or RESOURCE	SOURCE	IDENTIFIER
**Antibodies**		
ARFRP1	Sigma-Aldrich	Cat # HPA047028, RRID:AB_2679908
BCL2	Proteintech	Cat # 12789-1-AP, RRID:AB_2227948
CAPNS1, Calpain S1	Thermo Fisher Scientific	Cat # PA5-82266, RRID:AB_2789426
c-Myc	Covance	Cat # PRB-150C-200, RRID:AB_10063561
c-Myc (9E10)	Santa Cruz Biotechnology	Cat # sc-40, RRID:AB_627268
COX IV (3E11)	Cell Signaling Technology	Cat # 4850, RRID:AB_2085424
CUL1	Cell Signaling Technology	Cat # 4995, RRID:AB_2261133
CUL2 (EPR3104(2))	Abcam	Cat # ab166917
CUL3	Cell Signaling Technology	Cat # 2759, RRID:AB_2086432
CUL4A	Cell Signaling Technology	Cat # 2699, RRID:AB_2086563
CUL4B	Proteintech	Cat # 12916-1-AP, RRID:AB_208669
CUL5	Abcam	Cat # ab264284
DIS3	Thermo Fisher Scientific	Cat # PA5-78427, RRID:AB_2735722
EEA1 (H-300)	Santa Cruz Biotechnology	Cat # sc-33585, RRID:AB_2277714
FLAG (M2)	Sigma-Aldrich	Cat # F3165, RRID:AB_259529
GAPDH (0411)	Santa Cruz Biotechnology	Cat # sc-47724, RRID:AB_627678
GFP (clones 7.1 and 13.1)	Roche	Cat # 11814460001, RRID:AB_390913
HK1 (R.457.3)	Thermo Fisher Scientific	Cat # MA5-14789, RRID:AB_10987238
IST1	GeneTex	Cat # GTX101972, RRID:AB_1241005
KCMF1	Sigma-Aldrich	Cat # HPA030383, RRID:AB_10599977
LC3B	Thermo Fisher Scientific	Cat # PA1-46286, RRID:AB_2234770
Mef2	Gift from Dr. Eileen Furlong	N/A
Mono- and polyubiquitinylated conjugates (FK2)	Enzo Life Sciences	Cat # BML-PW8810, RRID:AB_10541840
NAA30	Sigma-Aldrich	Cat # HPA057824, RRID:AB_2683539
p62/SQSTM1 (D-3)	Santa Cruz Biotechnology	Cat # sc-28359, RRID:AB_628279
RBX1 (EPR20185)	Abcam	Cat # ab221548
RBX2/RNF7 (EPR12001)	Abcam	Cat # ab181986
RGS10 (EPR10657(B))	Abcam	Cat # ab154172
RSPRY1	Thermo Fisher Scientific	Cat # PA5-32048, RRID:AB_2549521

SLC10A7	Sigma-Aldrich	Cat # SAB2102163, RRID:AB_10607609
UBE2A and UBE2B, known as RAD6	Abcam	Cat # ab31917, RRID:AB_777604
UBE2F/NCE1 (EPR12932)	Abcam	Cat # ab185234
UBE2M (EPR5333)	Abcam	Cat # ab109507, RRID:AB_10892148
UBR1	Bethyl Laboratories	Cat # A302-988A-M, RRID:AB_2780899
UBR2	Abcam	Cat # ab217069
UBR4/P600	Abcam	Cat # ab86738, RRID:AB_1952666
UBR5/EDD (D6O8Z)	Cell Signaling Technology	Cat # 65344, RRID:AB_2799679
V5	Thermo Fisher Scientific	Cat # R960CUS, RRID:AB_2792973
Vinculin (EPR8185)	Abcam	Cat # ab129002, RRID:AB_11144129
anti-Mouse HRP-linked (sheep)	Cytiva	Cat # NA931, RRID:AB_772210
anti-Rabbit HRP-linked (donkey)	Cytiva	Cat # NA934, RRID:AB_772206
anti-Mouse Alexa Fluor 488 (goat)	Jackson ImmunoResearch	Cat # 115-547-003, RRID:AB_2338869
anti-Rabbit Alexa Fluor 594 (goat)	Jackson ImmunoResearch	Cat # 111-586-003 RRID:AB_2338066
anti-Mouse Alexa Fluor 488 (goat)	Thermo Fisher Scientific	Cat # A-11029, RRID:AB_2534088
anti-Rabbit Alexa Fluor 555 (donkey)	Thermo Fisher Scientific	Cat # A-31572, RRID:AB_ 162543
**Bacterial and virus strains**		
One Shot TOP10 Chemically Competent *E. coli*	Invitrogen	Cat # C404006
One Shot Stbl3 Chemically Competent *E. coli*	Invitrogen	Cat # C737303
Lentivirus for plasmid transduction	Generated by standard methods using packaging plasmids pMD2.G and psPAX2 (see Aregger et al., 2020)	N/A
**Chemicals, peptides, and recombinant proteins**		
Acetyl Coenzyme A, [acetyl-1-14C]	PerkinElmer	Cat # NEC313050UC
Ammonium chloride	Sigma-Aldrich	Cat # 213330
Aprotinin	Roche	Cat # 10981532001
Bafilomycin A1	MedChem Express	Cat # HY-100558
Bortezomib	Calbiochem	Cat # 5.04314
Bovine Serum Albumin (BSA)	Sigma-Aldrich	Cat # A7906
BbsI-HF	NEB	Cat # R3539S
cOmplete EDTA-free protease inhibitor cocktail	Roche	Cat # 5056489001
DharmaFECT 1 Transfection Reagent	Horizon Discovery	Cat # T-2001-02
DMEM high glucose	Sigma-Aldrich	Cat # D6546
DMSO	Sigma-Aldrich	Cat # D8418
DPBS, no calcium, no magnesium	Gibco	Cat # 14190144
Dynabeads Protein G	Invitrogen	Cat # 10003D
EcoRI-HF	New England Biolabs	Cat # R3101S
Fetal Bovine Serum	Sigma-Aldrich	Cat # F7524

Fetal Bovine Serum (CRISPR screens)	Life Technologies	Cat # 12483-020
Formaldehyde 16% (w/v), Methanol-free	Thermo Fisher Scientific	Cat # 28908
ibidi Mounting Medium With DAPI	ibidi GmbH	Cat # 50011
IMDM (Iscove's Modified Dulbecco's Medium)	Gibco	Cat # 12440061
Laemmeli SDS-PAGE sample buffer, 2X	Sigma-Aldrich	Cat # S3401
Laemmli SDS sample buffer, reducing (6X)	Alfa Aesar	Cat # J61337
Leupeptin	Sigma-Aldrich	Cat # L2884
L-Glutamine	Sigma-Aldrich	Cat # G7513
Lipofectamine 2000 transfection reagent	Invitrogen	Cat # 11668019
LysoView 488	Biotium	Cat # 70067
Lysyl Endopeptidase, endoLysC	Wako	Cat # 121-05063
MG132	Calbiochem	Cat # 474791
NEBNext Ultra II Q5 Master Mix	New England Biolabs	Cat # M0544L
Nonfat dried milk powder	Applichem	Cat # A0830
Opti-MEM I Reduced Serum Medium	Gibco	Cat # 31985070
PBS tablets	Gibco	Cat # 18912014
Penicillin-Streptomycin	Sigma-Aldrich	Cat # P0781
Penicillin-Streptomycin (CRISPR screens)	Life Technologies	Cat # 15140122
Hexadimethrine bromide (polybrene)	Sigma-Aldrich	Cat # H9268
Rhodamine Phalloidin	Thermo Fisher Scientific	Cat # R415
Alexa Fluor 488 Phalloidin	Thermo Fisher Scientific	Cat # A12379
TRITC-conjugated Phalloidin	Sigma-Aldrich	Cat # P1951
Pierce Streptavidin Magnetic Beads	Thermo Fisher Scientific	Cat # 88817
ProLong antifade mountant with NucBlue	Invitrogen	Cat # P36983
PureLink RNase A	Invitrogen	Cat # 12091021
Puromycin	Sigma-Aldrich	Cat # P8833
SD-Ura medium	Sunrise Science Products	Cat # 1703-500
siRNA Buffer, 5X	Horizon Discovery	Cat # B-002000-UB-100
T4 DNA ligase	NEB	Cat # M0202S
TBS Buffer, 20X	Thermo Fisher Scientific	Cat # 28358
Tris/Glycine Buffer for Western Blots and Native Gels, 10x	Bio-Rad	Cat # 1610771
Tris/Glycine/SDS, 10x	Bio-Rad	Cat # 1610772
Triton X-100	Sigma-Aldrich	Cat # T8787
TrypLE Express Enzyme	Gibco	Cat # 12605010
Trypsin, sequencing grade	Promega	Cat # V5111
Trypsin-EDTA (0.25%) (CRISPR screens)	Gibco	Cat # 25200056
Trypsin-EDTA, 10X	Gibco	Cat # 15400-054
Ultima Gold F scintillation mixture	PerkinElmer	Cat # 6013179
UltraPure DNase/RNase-Free Distilled Water	Invitrogen	Cat # 10977023
Vectashield antifade mounting medium	Vector Labs	Cat # H-1000
WST-1 reagent	Roche	Cat # 5015944001
XmaI	New England Biolabs	Cat # R0180S
X-tremeGENE 9 DNA Transfection Reagent	Roche	Cat # 6365809001
**Critical commercial assays**		
ATP Bioluminescence Assay Kit	Sigma-Aldrich	Cat # FLAA-1KT
Bio-Rad Bradford Protein Assay Dye Reagent	Bio-Rad	Cat # 5000006

GeneJET Gel Extraction Kit	Thermo Fisher Scientific	Cat # K0692
GeneJET Plasmid Miniprep Kit	Thermo Fisher Scientific	Cat # K0503
MycoAlert Mycoplasma Detection Kit	Lonza	Cat # LT07-318
NucleoBond Xtra Midi Kit	Macherey-Nagel	Cat # 740410.100
Pierce BCA protein assay kit	Thermo Scientific	Cat # 23225
Pierce SuperSignal West Pico PLUS Chemiluminescent Substrate	Thermo Fisher Scientific	Cat # 34578
PureLink Quick Gel Extraction Kit	Invitrogen	Cat # K210025
Q5 Site-Directed Mutagenesis Kit	New England Biolabs	Cat # E0552S
Qubit dsDNA BR Assay Kit	Invitrogen	Cat # Q32853
TAGZyme Qcyclase/pGAPase Enzymes	Qiagen	Cat # 34342
TMTpro 16plex Label Reagent Set	Thermo Fisher Scientific	Cat # A44521
Trans-Blot Turbo RTA Mini 0.2 µm Nitrocellulose Transfer Kit	Bio-Rad	Cat # 1704270
Wizard Genomic DNA Purification Kit	Promega	Cat # A1120
**Deposited data**		
CRISPR screen sequencing data	This paper	GEO database: GSE221447
N-terminal acetylation proteomics of HAP1 WT and NatC KO cells	This paper	ProteomeXchange: PXD034992
LFQ shotgun proteomics of HAP1 WT and NatC KO cells	This paper	ProteomeXchange: PXD034104
TMT proteomics of HAP1 WT and *NAA30*-KO treated with siCtrl or siUBR4	This paper	ProteomeXchange: PXD034410
Additional raw data including uncropped immunoblot images	This paper	Source Data file
**Experimental models: Cell lines**		
Human: HAP1 WT	Horizon Discovery	Cat # C361; RRID:CVCL_Y019
Human: HAP1 *NAA30*-KO	Horizon Discovery	Cat # HZGHC006637c010; RRID:CVCL_SZ74
Human: HAP1 *NAA35*-KO	Horizon Discovery	Cat # HZGHC006636c011; RRID:CVCL_SZ75
Human: HAP1 *NAA38*-KO (referred to as KO2 in original ref)	Dr. Scott Dixon (Cao et al., 2019)	N/A
Human: HAP1 CtrlB	Dr. Scott Dixon (Cao et al., 2019)	N/A
Human: HeLa	ATCC	Cat # CCL-2; RRID:CVCL_0030
Human: MDA-MB-231 (parental cell line)	ATCC	Cat # HTB-26 RRID:CVCL_0062
Human: MDA-MB-231 (monoclonal parental cell line)	This paper	N/A
Human: MDA-MB-231 *NAA30*-KO	This paper	N/A
Human: MDA-MB-231 WT underwent CRISPR procedure with sg*NAA30*, but is unmodified	This paper	N/A
Human: MDA-MB-231 WT underwent CRISPR procedure with non-targeting control sgRNA	This paper	N/A
**Experimental models: *Drosophila melanogaster* strains**		
Oregon R	Lab collection	N/A
y^1	Bloomington Drosophila Stock Center	RRID:BDSC_169
y^1, Naa30A$^{\Delta 74}$/FM0	This paper	N/A

w, y^1, Naa30A$^{\Delta74}$/FM0	This paper	N/A
w, y^1, Naa30A$^{\Delta74}$/FM0/DP (1;Y) y^+	This paper	N/A
w, y^1, Naa30A$^{\Delta74}$/FM0;;UAS-UbcE2M/TM3,sb	This paper	N/A
w;; Naa30A-genomic-8myc	This paper	N/A
w; Naa30A-genomic-8myc	This paper	N/A
w;; Da-Gal4	Wodarz et al., 1995	N/A
y^1 P{EPgy2}Naa30A^{EY10202} w^{67c23}	Bloomington Drosophila Stock Center	RRID:BDSC_16976
w;; Mhc-Gal4	Bloomington Drosophila Stock Center	RRID:BDSC_55133
w;; UAS-UcbE2M3xH	Zurich ORFeome Project (FlyOrf)	Cat # F003044
**Experimental models:** ***Saccharomyces cerevisiae strains***		
BY4742; mak3::kanMX4	EUROSCARF	Cat # Y15470
BY4741; ARL3::GFP-HISMX6	Invitrogen	Cat # 95700
BY4741; ARL3::GFP-HISMX6; mak3::kanMX4	Aksnes et al., 2013	N/A
BY4741; ARL3::GFP-HISMX6; pBEVY-U-empty	Osberg et al., 2016	N/A
BY4741; ARL3::GFP-HISMX6; mak3::kanMX4; pBEVY-U-empty	Osberg et al., 2016	N/A
BY4741; ARL3::GFP-HISMX6; mak3::kanMX4; pBEVY-U-DmNAA30	This paper	N/A
**Recombinant DNA**		
pMD2.G	Addgene	Cat # 12259; RRID:Addgene_12259
psPAX2	Addgene	Cat # 12260; RRID:Addgene_12260
TKOv3 library (71,090 gRNAs)	Hart et al., 2017	Cat # 90294; RRID:Addgene_90294
pCMV6-MI-UBE2M-Myc-DDK (native)	OriGene	Cat # RC208946
pCMV6-MG-UBE2M-Myc-DDK	This paper	N/A
pCMV6-MA-UBE2M-Myc-DDK	This paper	N/A
pCMV6-MD-UBE2M-Myc-DDK	This paper	N/A
pCMV6-ME-UBE2M-Myc-DDK	This paper	N/A
pCMV6-ML-UBE2M-Myc-DDK	This paper	N/A
pCMV6-MF-UBE2M-Myc-DDK	This paper	N/A
pCMV6-MY-UBE2M-Myc-DDK	This paper	N/A
pCMV6-MP-UBE2M-Myc-DDK	This paper	N/A
pCMV6-Empty	This paper	
pUC-UBE2M-V5-P2A-GST-GFP	VectorBuilder, custom-made	See Source Data file for complete sequence
pcDNA3.1-NAA30-WT-V5	Starheim et al., 2009	N/A
pcDNA3.1-NAA30-E321A-V5	This paper	N/A
pcDNA3.1-LacZ-V5-His	Invitrogen	N/A
pcDNA3.1-Empty	Invitrogen	N/A
pcDNA6.2-hUBR4-V5-Lumio	Tasaki et al., 2009	N/A
pCDH-EF1-MCS-T2A-GFP	SBI	Cat # CD527A-1
pCDH-EF1-UBR4N-FLAG-T2A-GFP	Hunt et al., 2019	N/A
pCDH-EF1-UBR4C-FLAG-T2A-GFP	Hunt et al., 2019	N/A
pBEVY-U-empty	Miller et al., 1998	N/A
pBEVY-U-HA-DmNAA30	This paper	N/A
pBEVY-U-HA-hNAA30	Osberg et al., 2016	N/A
pSpCas9(BB)-2A-GFP	Addgene	Cat #48138; RRID:Addgene_48138

pSpCas9(BB)-2A-GFP-NAA30	This study	N/A
pSpCas9(BB)-2A-GFP-non-targeting control	This study	N/A
ON-TARGETplus Human KCMF1 (56888) siRNA – SMARTpool	Horizon Discovery	Cat # L-017900-01-0050
ON-TARGETplus Human UBE2A (7319) siRNA – SMARTpool	Horizon Discovery	Cat # L-009424-00-0005
ON-TARGETplus Human UBE2B (7320) siRNA – SMARTpool	Horizon Discovery	Cat # L-009930-00-0005
ON-TARGETplus Human UBR1 (197131) siRNA – SMARTpool	Horizon Discovery	Cat # L-010691-00-0005
ON-TARGETplus Human UBR2 (23304) siRNA – SMARTpool	Horizon Discovery	Cat # L-006954-00-0005
ON-TARGETplus Human UBR4 (23352) siRNA – SMARTpool	Horizon Discovery	Cat # L-014021-01-0005
ON-TARGETplus Human UBR5 (51366) siRNA – SMARTpool	Horizon Discovery	Cat # L-007189-00-0005
ON-TARGETplus Non-targeting Pool	Horizon Discovery	Cat # D-001810-10-05
**Software and algorithms**		
Adobe Illustrator 2020	Adobe Inc https://www.adobe.com	RRID:SCR_010279
Adobe Photoshop CS5	Adobe Inc https://www.adobe.com	RRID:SCR_014199
BAGEL	Hart et al., 2017 https://github.com/hart-lab/bagel	N/A
Bowtie v0.12.8,	https://bowtie-bio.sourceforge.net	RRID:SCR_005476
CHOPCHOP v3	Labun et al., 2019 https://chopchop.cbu.uib.no/	RRID:SCR_015723
DSRC Integrative Ortholog Prediction Tool (DIOPT)	Hu et al., 2011 https://www.flyrnai.org/cgi-bin/DRSC_orthologs.pl	
FACSDiva 9.0.1	BD Biosciences https://www.bdbiosciences.com	RRID:SCR_001456
FlowJo version 10.8.1	BD Biosciences https://www.flowjo.com/	RRID:SCR_008520
Flybase database	https://flybase.org/	RRID:SCR_006549
GraphPad Prism version 9.3.1	GraphPad Software https://www.graphpad.com/	RRID:SCR_002798
HoloMonitor App Suite version 3.5.0.214	PHI AB https://phiab.com/holomonitor/	RRID:SCR_019231
Image Lab version 6.0.1	Bio-Rad https://www.bio-rad.com	RRID:SCR_014210
ImageJ/Fiji version 2.1.0/1.53c	Schindelin et al., 2012 https://imagej.net/	RRID:SCR_002285
IMARIS version 9.7.2	Oxford instruments https://imaris.oxinst.com/	RRID:SCR_00730
Mascot Distiller version 2.7.1.0	Matrix Science Ltd https://www.matrixscience.com	RRID:SCR_000307
MaxQuant versions 2.0.1.0 and 1.6.17.0	Cox and Mann, 2008 https://www.maxquant.org/	RRID:SCR_014485
Microsoft Excel	Microsoft https://www.microsoft.com/	RRID:SCR_016137
Perseus version 1.6.15.0	Tyanova et al., 2016 https://maxquant.net/perseus/	RRID:SCR_015753
SwissProt datase	https://www.expasy.org/resources/uniprotkb-swiss-prot	RRID:SCR_021164
Tecan i-control software version 2.0.10.0	Tecan https://www.tecan.com/	RRID:SCR_016771

Other		
Amersham Protran Western blotting membrane, nitrocellulose, 0.2 µm	Cytiva	Cat #GE10600001
Andor Dragonfly 500 confocal microscopy	Oxford instruments	N/A
Axiovert 200M widefield fluorescence microscope	Carl Zeiss AG	N/A
ChemiDoc XRS+ imaging system	Bio-Rad	RRID:SCR_019690
Coverslips, 12 mm Ø	Paul Marienfeld GmbH	Cat # 0117520
Gel Doc EZ imaging system	Bio-Rad	N/A
HoloMonitor M4 system	Phase Holographic Imaging PHI	RRID:SCR_019231
ibiTreat µ-Slide 4-well	ibidi GmbH	Cat # 80426
Illustra NAP-10 Columns	Cytiva	Cat # 17-0854-01
Infinite 200Pro plate reader	Tecan	N/A
LSRFortessa Cell Analyzer	BD Bioscience	RRID:SCR_018655
Lunatic microfluidic device	Unchained Labs	
OMIX C18 tips	Agilent Technologies	Cat # A57003100
Orbitrap Fusion Lumos mass spectrometer	Thermo Scientific	RRID:SCR_020562
Orbitrap Velos mass spectrometer	Thermo	RRID:SCR_020550
P81 phosphocellulose paper	Millipore	Cat # 3698-915
ReproSil-Pur Basic-C18-HD, 5 µm	Dr. Maisch HPLC GmbH	Cat # R15.b9h
SampliQ SPE C18 cartridge	Agilent Technologies	Cat # 5982-1135
Sony SH800 cell sorter	Sony Biotechnology	RRID:SCR_018066
S-Trap mini columns	ProtiFi	Cat # C02-mini-40
TC20 automated cell counter	Bio-Rad	N/A
Trans-Blot Turbo transfer System	Bio-Rad	N/A
Tri-Carb 2900TR Liquid Scintillation Analyzer	PerkinElmer	N/A
µPAC column	PharmaFluidics	N/A
Whatman Grade 3MM Chr Cellulose Chromatography Paper	Cytiva	Cat # 3030-672
